# Canadian Network for Mood and Anxiety Treatments (CANMAT) 2023 Update on Clinical Guidelines for Management of Major Depressive Disorder in Adults: Réseau canadien pour les traitements de l'humeur et de l'anxiété (CANMAT) 2023 : Mise à jour des lignes directrices cliniques pour la prise en charge du trouble dépressif majeur chez les adultes

**DOI:** 10.1177/07067437241245384

**Published:** 2024-05-06

**Authors:** Raymond W. Lam, Sidney H. Kennedy, Camelia Adams, Anees Bahji, Serge Beaulieu, Venkat Bhat, Pierre Blier, Daniel M. Blumberger, Elisa Brietzke, Trisha Chakrabarty, André Do, Benicio N. Frey, Peter Giacobbe, David Gratzer, Sophie Grigoriadis, Jeffrey Habert, M. Ishrat Husain, Zahinoor Ismail, Alexander McGirr, Roger S. McIntyre, Erin E. Michalak, Daniel J. Müller, Sagar V. Parikh, Lena C. Quilty, Arun V. Ravindran, Nisha Ravindran, Johanne Renaud, Joshua D. Rosenblat, Zainab Samaan, Gayatri Saraf, Kathryn Schade, Ayal Schaffer, Mark Sinyor, Claudio N. Soares, Jennifer Swainson, Valerie H. Taylor, Smadar V. Tourjman, Rudolf Uher, Michael van Ameringen, Gustavo Vazquez, Simone Vigod, Daphne Voineskos, Lakshmi N. Yatham, Roumen V. Milev

**Affiliations:** 1 Department of Psychiatry, 8166 University of British Columbia, Vancouver, BC, Canada; 2 Department of Psychiatry, 7938 University of Toronto, Toronto, ON, Canada; 3 Department of Psychiatry, 7235 University of Saskatchewan, Saskatoon, SK, Canada; 4 Department of Psychiatry, 2129 University of Calgary, Calgary, AB, Canada; 5 Department of Psychiatry, 5620 McGill University, Montréal, QC, Canada; 6 Department of Psychiatry, 6363 University of Ottawa, Ottawa, ON, Canada; 7 Department of Psychiatry, 4257 Queen's University, Kingston, ON, Canada; 8 Department of Psychiatry, 5622 Université de Montréal, Montréal, QC, Canada; 9 Department of Psychiatry and Behavioural Neurosciences, 3710 McMaster University, Hamilton, ON, Canada; 10 Department of Family and Community Medicine, 7938 University of Toronto, Toronto, ON, Canada; 11 Department of Psychiatry, 1259 University of Michigan, Ann Arbour, MI, USA; 12 Office of Research Services, 4614 Huron University, London, ON, Canada; 13 Department of Psychiatry, 3158 University of Alberta, Edmonton, AB, Canada; 14 Department of Psychiatry, 3688 Dalhousie University, Halifax, NS, Canada

**Keywords:** depressive disorders, major depressive disorder, clinical practice guidelines, adult psychiatry, pharmacotherapy, psychotherapy, electroconvulsive therapy, systematic reviews, evidence-based medicine

## Abstract

**Background:**

The Canadian Network for Mood and Anxiety Treatments (CANMAT) last published clinical guidelines for the management of major depressive disorder (MDD) in 2016. Owing to advances in the field, an update was needed to incorporate new evidence and provide new and revised recommendations for the assessment and management of MDD in adults.

**Methods:**

CANMAT convened a guidelines editorial group comprised of academic clinicians and patient partners. A systematic literature review was conducted, focusing on systematic reviews and meta-analyses published since the 2016 guidelines. Recommendations were organized by lines of treatment, which were informed by CANMAT-defined levels of evidence and supplemented by clinical support (consisting of expert consensus on safety, tolerability, and feasibility). Drafts were revised based on review by patient partners, expert peer review, and a defined expert consensus process.

**Results:**

The updated guidelines comprise eight primary topics, in a question-and-answer format, that map a patient care journey from assessment to selection of evidence-based treatments, prevention of recurrence, and strategies for inadequate response. The guidelines adopt a personalized care approach that emphasizes shared decision-making that reflects the values, preferences, and treatment history of the patient with MDD. Tables provide new and updated recommendations for psychological, pharmacological, lifestyle, complementary and alternative medicine, digital health, and neuromodulation treatments. Caveats and limitations of the evidence are highlighted.

**Conclusions:**

The CANMAT 2023 updated guidelines provide evidence-informed recommendations for the management of MDD, in a clinician-friendly format. These updated guidelines emphasize a collaborative, personalized, and systematic management approach that will help optimize outcomes for adults with MDD.

## Résumé

**Contexte:**

Le Réseau canadien pour les traitements de l’humeur et de l’anxiété (CANMAT) a publié les dernières lignes directrices cliniques pour le traitement du trouble dépressif majeur (TDM) en 2016. Grâce aux avancées dans le domaine, une mise à jour était nécessaire afin d’incorporer les nouvelles données probantes et de fournir des recommandations nouvelles et révisées pour l’évaluation et le traitement du TDM chez les adultes.

**Méthodes:**

CANMAT a convoqué un groupe de rédaction des lignes directrices composé de cliniciens universitaires et de patients partenaires. Une revue systématique de la littérature a été menée, qui était axée sur les revues systématiques et les méta-analyses publiées depuis les lignes directrices de 2016. Les recommandations ont été organisées par lignes de traitement, qui ont été éclairées par les niveaux de preuve définis par CANMAT et complétées par un soutien clinique (constitué d'un consensus d'experts sur la sécurité, la tolérabilité et la faisabilité). Les ébauches ont été révisées sur la base d'un examen par des patients partenaires, d'un examen par des pairs experts et d'un processus de consensus d'experts défini.

**Résultats:**

Les lignes directrices mises à jour comprennent huit sujets principaux, sous forme de questions et réponses, qui cartographient le parcours de soins du patient, de l'évaluation à la sélection de traitements fondés sur des données probantes, en passant par la prévention des récidives et les stratégies en cas de réponse inadéquate. Les lignes directrices adoptent une approche personnalisée des soins qui met l’accent sur la prise de décisions partagée, qui reflète les valeurs, les préférences et les antécédents de traitement du patient souffrant de TDM. Les tableaux offrent de nouvelles recommandations mises à jour pour les traitements psychologiques, pharmacologiques, de style de vie, de médecine complémentaire et al.ternative, de santé numérique et de neuromodulation. Les mises en garde et les limites des preuves sont mises en évidence.

**Conclusions:**

Les lignes directrices mises à jour de CANMAT 2023 fournissent des recommandations fondées sur des données probantes pour la prise en charge du TDM, dans un format convivial pour les cliniciens. Ces lignes directrices mises à jour mettent l'accent sur une approche de gestion collaborative, personnalisée et systématique qui contribuera à optimiser les résultats pour les adultes souffrant de TDM.

## Introduction

Depressive illnessessuch as major depressive disorder (MDD) are common mental health conditions that significantly impact a person's quality of life and increase their risk of developing other health problems. Despite international calls to prioritize the recognition and treatment of MDD, only 20% of individuals with MDD will receive adequate treatment. Clinical guidelines are one tool for standardizing treatments to improve clinical care.

The Canadian Network for Mood and Anxiety Treatments (CANMAT) last published evidence-based guidelines for managing MDD in 2016 (previous iterations were published in 2001 and 2009). Since then, new research has emerged and new and updated treatment options for MDD are available. This 2023 update provides healthcare professionals with the latest evidence-informed recommendations for assessing and managing MDD. The updated guidelines focus on personalized care for individuals with MDD, considering their needs, preferences, and treatment history. They also highlight the importance of collaborating with patients in their care decisions and choice of evidence-based treatment options. To this end, patient partners are included in the core editorial group and as authors and reviewers of these guidelines.

The guidelines development process is similar to previous CANMAT guidelines, with some exceptions. The familiar question–answer format is retained because clinicians continue to affirm its clinical practicality and ease of use. Questions were developed by a core editorial group with feedback from patient partners and organized into eight primary topics representing a patient care journey from assessment to acute treatment and maintenance, including treatment options for difficult-to-treat depression (DTD). We also incorporated lifestyle interventions such as exercise, nutrition, and sleep hygiene into treatment plans. The evidence review process focused on meta-reviews of systematic reviews and meta-analyses published since 2015 (the literature search end date for the CANMAT 2016 guidelines), supplemented by other studies when those were not available. To enhance readability, only selected references are included in this publication; a complete citation list and summary of evidence reviews are available in the online supplemental materials (https://osf.io/8tfkp/).

Throughout these guidelines, all recommendations are accompanied by the level of evidence available to support each graded line of treatment. In addition, clinical support is used to rank recommendations, based on expert consensus from the CANMAT editorial group on factors including safety, tolerability, and feasibility, where possible. A rigorous method ensures consensus among the editorial group, and new or controversial topics are highlighted to clarify the rationale for recommendations.

The scope of these guidelines is the management of MDD in adults, with a target audience of community-based psychiatrists and mental health providers. CANMAT previously published guidelines for bipolar disorder, in collaboration with the International Society for Bipolar Disorders (ISBD), and clinical guidelines for perinatal mood and anxiety-related disorders are in development; guidelines for other populations such as geriatric and pediatric depression are in planning. As done with previous versions, CANMAT will produce briefer summaries of the guidelines for clinicians, such as the CANMAT Pocket Guide to Depression. Additionally, the CHOICE-D Patient and Family Guide to Depression Treatment will be revised to reflect the content of the updated guidelines for patient and public knowledge dissemination.

## Methods

### Evidence Review

CANMAT convened a guidelines editorial group, comprised of academic clinicians (*n* = 43, representing diversity in seniority, region, and expertise) and a patient partner (KS), to direct and manage the 2023 update. Methods, search strategies, and evidence tables are detailed on the project website on the Open Science Framework (OSF) (https://osf.io/8tfkp/). High-quality, large-sample randomized controlled trials (RCTs) remain the gold standard for evidence. However, owing to the sheer volume of available RCTs, we prioritized systematic reviews and meta-analyses that synthesize many RCTs, for much of the evidence used and cited in these guidelines. We also recognize the limitations of meta-analyses; hence, we complemented them with results from large RCTs when making recommendations.

We conducted comprehensive literature searches using appropriate keywords to identify systematic reviews and meta-analyses published between 1 January 2015 and 31 May 2023. In addition, we searched for RCTs and other studies when systematic reviews/meta-analyses were unavailable. Cross-referencing bibliographies, reviews of other major reports and guidelines, and expert feedback identified additional studies. Two independent reviewers selected relevant studies, with consensus adjudication by a third reviewer in cases of disagreement (Supplemental Figure e1). Data from the included studies were extracted by research staff in tabular format. The summary of evidence tables is provided online (https://osf.io/8tfkp/).

### Grading of Recommendations

The strength of evidence was evaluated based on the level of evidence criteria used in previous CANMAT guidelines (Table A). We assessed the systematic reviews and meta-analyses for quality and risk of bias using modified global ratings from Grading of Recommendations Assessment, Development, and Evaluation (GRADE). The risk of bias for each study was assessed for methodology elements and rated as “Low” (few missing elements that are unlikely to affect the outcome), “Medium” (some missing elements that may possibly affect outcomes), and “High” (many missing or unclear elements, or clear bias in one element that is likely to affect the outcome). RCTs and other studies were considered when systematic reviews and meta-analyses were unavailable, with small-sample RCTs defined as those involving less than 30 participants per randomized condition. We use symbols to denote these levels of evidence (Table A) when referencing in the text (see conventions used in this document).

**Table A.**
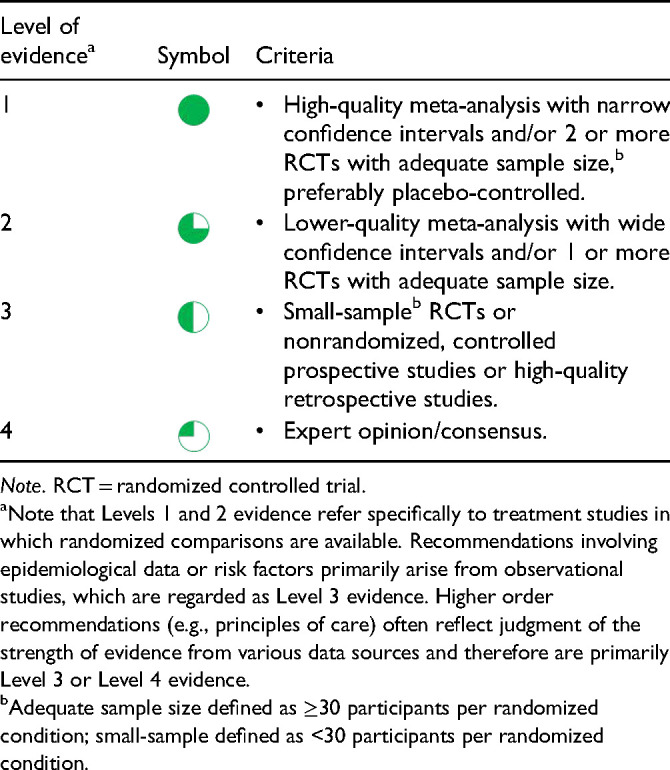
CANMAT Criteria for Level of Evidence.

Treatment recommendations are then organized along the lines of treatment, based on both strength of the evidence and clinical support (Table B). Clinical support reflects expert consensus by the CANMAT editorial group on tolerability, safety, and feasibility. First-line treatments require higher-quality evidence (Level 1 or Level 2 evidence) and generally should be considered first in decision-making. Second- and third-line treatments should usually be considered only when first-line treatments are ineffective or unavailable. Treatments with Level 1 evidence for efficacy may be downgraded to second-line or third-line recommendations due to clinical concerns such as safety and side effect profile. Note that recommendations for principles of care (e.g. in Q.1, Q.2 and Q.5) are based primarily on Level 3 or Level 4 evidence, such as expert consensus.

**Table B.** CANMAT Criteria for Line of Treatment.

Line of treatment	Criteria
First line	Level 1 or Level 2 evidence, plus clinical support^a^
Second line	Level 3 evidence or higher, plus clinical support^a^
Third line	Level 4 evidence or higher, plus clinical support^a^

a Clinical support refers to the application of expert consensus by the Canadian Network for Mood and Anxiety Treatments (CANMAT) editorial group to ensure that evidence-supported interventions are feasible and relevant to clinical practice. Therefore, treatments with higher levels of evidence may be downgraded to lower lines of treatment due to clinical issues such as side effects or safety profiles.

### Feedback and Consensus Process

Drafts of these guidelines were reviewed by patient partners (see Acknowledgements) who received a small stipend for their feedback. A formal iterative process was used to achieve consensus among the CANMAT authors for the final recommendations, with full details available online (https://osf.io/8tfkp/). In summary, consensus was sought after each level of review, initially within the authors of each section, then by the core editorial group (consisting of the co-editors and the section editors), and finally by all co-authors. Co-authors were also invited to write a dissenting opinion if there was disagreement on a recommendation, to be published in the supplemental materials; however, no dissenting opinions were identified. External peer review was conducted via the *Canadian Journal of Psychiatry* review process and the final version incorporates revisions based on those reviews.

### Funding and Conflict of Interest

The guidelines process and publication were funded by internal CANMAT funds; no external support was sought or received. No honoraria were paid to authors and no professional editorial assistance was used. All authors disclosed potential conflicts of interest. CANMAT is a project-driven academic organization governed by a volunteer, unpaid Advisory Board with no permanent staff or dedicated offices. CANMAT activities involve research, knowledge translation (e.g., guidelines dissemination, national and international conferences, and publications) and continuing professional development (CPD). CANMAT has a Conflict-of-Interest policy that includes disclosures by all participants, and academic institutions accredit all CPD projects. CANMAT activities are funded from various sources: academic projects from peer review or philanthropic agencies, and conferences and CPD funded by universities and industry sponsors. CANMAT research studies are independently funded by agencies such as the Canadian Institutes for Health Research (CIHR) and are administrated by the academic institutions of the principal investigators. In the past 5 years (2019–2023), sources of CANMAT revenue (excluding CIHR and research funding) included government funding agencies (52% of revenue), national scientific conferences (20% of revenue), and publications (28%); no pharmaceutical or industry funding was received.

### Caveats and Limitations

While systematic reviews and meta-analyses are useful for summarizing data and are considered at the top of the evidence hierarchy, they reflect the quality of the original studies they synthesize. Their reliability can be limited by methodological factors, including the scope of the review, the criteria used for study selection, and the heterogeneity, quality, and generalizability of included studies. In addition, most meta-analyses assess the “risk of bias” to consider how findings could be influenced by factors such as author allegiance and funding source and whether adequate measures have been taken to minimize potential sources of bias. Yet, even meta-analyses with a low risk of bias have limitations, as their results may still be biased by including lower-quality studies, differ depending on methodological decisions, and be inconsistent with high-quality trials. Consequently, selecting evidence to include in clinical guidelines involves quality assessment. Sometimes, a single large high-quality RCT may provide a better guide for evidence than a meta-analysis that includes studies of lower quality, higher bias, or greater heterogeneity.

Evaluation of many treatments and complex interventions also pose challenges due to lack of blinding and choice of comparison conditions. For example, studies comparing psychological treatments to minimal control conditions (such as waitlist) may not be as clinically relevant as studies that compare novel treatments to standard or usual care. Further, RCTs may have limited generalizability due to restrictive inclusion criteria. Accordingly, evidence from observational studies can complement controlled trials and is used where appropriate. Finally, while meta-analyses can track the evolution of evidence over time, it is recognized that early investigations of novel treatments tend to show larger effect sizes that decrease over subsequent years, particularly when the novel treatment becomes standard care. Given the rapid pace of research, CANMAT will review these guidelines again in 7 years (2030).

In summary, owing to the variability of individual patient cases and limitations of the evidence base, few definitive or universally preferred treatments apply to all patients. Therefore, these CANMAT recommendations should be viewed as guidance for clinicians to consider within the context of each patient, rather than as established standards of care. Finally, since the CANMAT guidelines have a global reach and the evidence base is not country-specific, we note that some recommended treatments are not available in Canada and that regulatory requirements for medications vary from country to country.

### Conventions Used in This Document

Several conventions are used in these guidelines. In the summary tables, recommendations are ordered by line of treatment, followed by level of evidence, and then by alphabetical order. The exception is when treatments are grouped according to a class, such as medication mechanism of action. The symbols for the level of evidence presented in Table A (Level 1 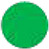, Level 2 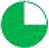, Level 3 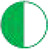, and Level 4 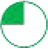) are used throughout the text to show the strength of evidence for statements and recommendations. To improve readability, we provide a selection of key references for each of the 8 primary questions instead of citing every statement. A full set of references is available online (https://osf.io/8tfkp/). Table C lists the abbreviations used in these guidelines.

**Table C.** List of Abbreviations.

Abbreviation	Definition
ACT	Acceptance and commitment therapy
ADHD	Attention-deficit hyperactivity disorder
AI	Artificial intelligence
BA	Behavioural activation
CAM	Complementary and alternative medicine
CANMAT	Canadian Network for Mood and Anxiety Treatments
CBASP	Cognitive behavioural analysis system of psychotherapy
CBT	Cognitive-behavioural therapy
CPD	Continuing professional development
CYP	Cytochrome P450
DBS	Deep brain stimulation
DHI	Digital health intervention
DLPFC	Dorsolateral prefrontal cortex
DSM-5-TR	Diagnostic and Statistical Manual, 5th edition, Text Revision
DSM-IV-TR	Diagnostic and Statistical Manual, 4th edition, Text Revision
DTD	Difficult-to-treat depression
ECG	Electrocardiography
ECT	Electroconvulsive therapy
EEG	Electroencephalography
GRADE	Grading of Recommendations Assessment, Development, and Evaluation
ICD	International Classification of Diseases
IPT	Interpersonal therapy
MAOI	Monoamine oxidase inhibitor
MBC	Measurement-based care
MBCT	Mindfulness-based cognitive therapy
MCT	Metacognitive therapy
MDD	Major depressive disorder
MDE	Major depressive episode
MI	Motivational interviewing
MST	Magnetic seizure therapy
NbN	Neuroscience-based nomenclature
NDRI	Norepinephrine-dopamine reuptake inhibitor
NMDA	N-methyl-D-aspartate
NSAID	Nonsteroidal anti-inflammatory drug
PDD	Persistent depressive disorder
PDT	Psychodynamic psychotherapy
PHQ	Patient health questionnaire
PST	Problem-solving therapy
RCT	Randomized controlled trial
rTMS	Repetitive transcranial magnetic stimulation
SDM	Shared decision-making
SNRI	Serotonin-norepinephrine reuptake inhibitor
SSRI	Selective serotonin reuptake inhibitor
STPP	Short-term psychodynamic psychotherapy
TBS	Theta burst stimulation
TCA	Tricyclic antidepressants
tDCS	Transcranial direct current stimulation
TMS	Transcranial magnetic stimulation
TRD	Treatment-resistant depression
USA	United States of America
VNS	Vagus nerve stimulation
WHO	World Health Organization

While various terms are used for lived experience, in these guidelines, the term *patient* refers to individuals with MDD in clinical treatment. We use the terms *mild*, *moderate*, and *severe* to describe the severity of a major depressive episode (MDE), based on the rating of symptom severity and degree of functional impairment, whichever is higher. We recommend the use of a validated rating scale to quantify these (see Q.5.a). As an example, an MDE of moderate severity could represent either moderately severe symptoms with mild–moderate functional impairment, or mild–moderate symptom severity with moderate impairment.

Due to a lack of empirical differentiation between the terms *relapse* and *recurrence*, we use them interchangeably. We use the term *adjunctive* to refer to adding a second medication or treatment to an initial one (thereby replacing the term *augmentation*), whereas *combination* refers to starting two medications or treatments at the same time. *Sex* is used to denote biological sex at birth and *gender* is used to denote socially constructed roles, behaviours, and identities. Given that psychiatric conditions have somatic symptoms and represent a subset of all medical conditions, we use the term *nonpsychiatric medical conditions* to denote such as heart disease, neurological disorders, diabetes mellitus, etc. Finally, we use text boxes to highlight new or controversial topics. For example, Box A discusses the use of a newer nomenclature for medications.

Box A. Neuroscience-Based Nomenclature for Medications.

The prevailing nomenclature for psychotropic medications is not based on mechanism of action but instead is based primarily on indication (e.g., antidepressants, anxiolytics, antipsychotics). Given that medications are often effective across a broad range of diagnostic categories, more scientific nomenclatures have emerged, including the Neuroscience-Based Nomenclature (NbN). In these guidelines, we will often use newer NbN terms to better describe medications according to mechanism of action, e.g., *serotonin-dopamine activity modulator* instead of atypical antipsychotic, and *medication indicated for anxiety* instead of anxiolytic. However, we will still use the older terms, such as *antidepressant*, because clinicians are still more familiar with these terms.

## Question 1. What are Important Issues for Assessment and Diagnosis?

According to the World Health Organization (WHO) statistics from 2020, over 300 million individuals worldwide are estimated to be living with MDD. Within Canada, MDD constitutes a significant public health concern. The Canadian Community Health Survey found that ∼12% of Canadians aged 12 and older reported a lifetime episode of MDD; 4.7% of Canadians experienced an MDE within the past year. Not only is MDD highly prevalent, it results in social and occupational impairment, diminished quality of life, and an elevated risk of death by suicide. The Global Burden of Disease 2019 study indicated that depression is the second-ranked medical cause of disability worldwide.

### Q.1.a. What are the Risk Factors for MDD?

Numerous risk factors for MDD have been identified, with varying strength of evidence (Table 1.1). Some are static and nonmodifiable historical factors such as a family history of depression and a history of adverse childhood experiences. Others are dynamic in that they may emerge and recede through the life span, such as psychiatric and nonpsychiatric medical illness, interpersonal or occupational stress, and bereavement. Clinicians should be aware of both static and dynamic factors to better address the full range of biological, psychological and social issues in case management (Table 1.2).

**Table 1.1.** Examples of Risk Factors for Major Depressive Disorder (MDD).

Static, nonmodifiable risk factors	Dynamic, potentially modifiable risk factors
Female sex Family history of mood disorders History of adverse childhood events/maltreatment Death of spouse	Chronic and nonpsychiatric medical illnesses Psychiatric comorbidities, especially anxiety disorders Alcohol and substance use disorders Insomnia, night shift work Periods of hormonal changes (e.g., puberty, pregnancy, postpartum, and perimenopause) Recent stressful life events Job strain/income inequality Bereavement Peer victimization/bullying/cyberbullying Gender dysphoria Sedentary lifestyle/screen time

**Table 1.2.**
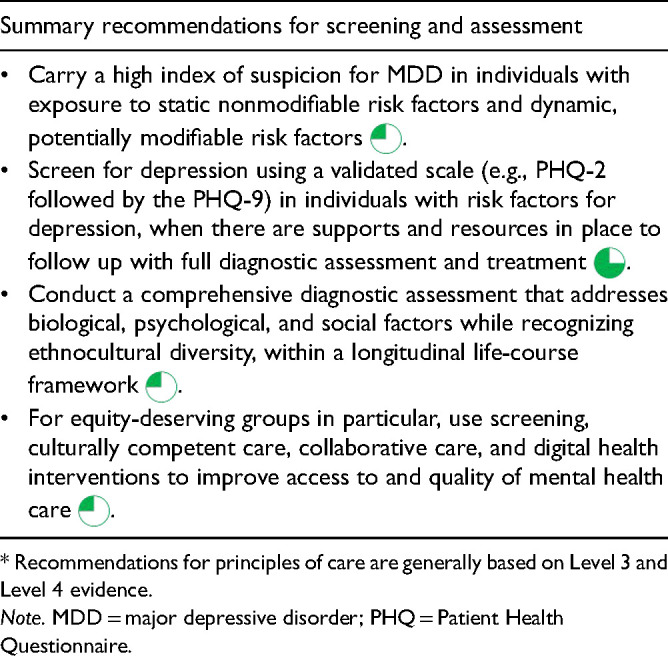
Recommendations* for Screening and Assessment.

#### Static, Nonmodifiable Risk Factors

Female sex is associated with a higher risk and prevalence of MDD than male, especially in youth and middle age; there is no sex difference in prevalence of MDD in older age. A family history of mood disorders substantially increases the overall risk for MDD. A history of adverse childhood events or maltreatment, especially emotional abuse, bullying, physical neglect, and parental loss, is also strongly associated with a higher risk of MDD (and other psychiatric conditions) in adulthood. Death of a spouse is another risk factor, independent of age and sex.

#### Dynamic, Potentially Modifiable Risk Factors

Many nonpsychiatric medical conditions, including chronic pain, cardiovascular illness, cancer, inflammatory bowel diseases, diabetes mellitus, sleep disorders, obesity, and acne, are associated with increased risk for MDD, with most conditions having a bidirectional relationship. Periods of hormonal changes in women, e.g., perinatal and perimenopausal periods, are also high-risk times for MDD.

There is a well-known association between alcohol use disorders and MDD, and there is evidence of a higher risk for MDD in other substance use disorders. For example, systematic reviews and meta-analyses show an association between opioid misuse/use disorders and depressive symptoms/disorders. Cannabis use during adolescence is also linked to an increased risk for depression in adulthood.

Stressful life events and circumstances, including interpersonal stress, job strain and insecurity, income inequality, and challenging living environment, are associated with an increased risk of developing MDD even when correcting for pre-existing depressive symptoms and comorbid other medical conditions. Bereavement is a normal process, but severe or prolonged grief also can increase the risk of an MDE. Other modifiable factors associated with a greater risk of MDD include a sedentary lifestyle, increased screen time watching television or using mobile devices, and shift work.

### Q.1.b. How and Who Should Be Screened for Depression?

Up to 60% of individuals with MDD are unrecognized or misidentified in primary care settings, and rates of under-detection are even higher in low- and middle-income countries. However, there is a lack of high-quality evidence to inform optimal strategies to improve detection and treatment uptake. Routine screening refers to having all patients (or at-risk groups) regularly complete screening procedures at prespecified times/intervals.

Major extant guidelines provide conflicting recommendations regarding routine screening for depression in adults who appear at average risk (i.e., with no apparent symptoms of depression or risk factors), in part because of inconsistent evidence to support the benefits of screening and the challenges and costs of implementation. For example, the 2013 Canadian Task Force for Preventative Health recommended against routine depression screening for adults with average risk, although this was graded as a weak recommendation because of low-quality evidence. The CANMAT 2016 guidelines recommended screening for individuals with risk factors when resources were available for further assessment and treatment. The United States Preventative Services Task Force recently updated its evidence review and concluded that routine depression screening is associated with moderate net benefit and improved outcomes after 6 to 12 months, particularly in settings that provide additional interventions for those who screen positive 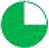. There are potential risks to routine screening, e.g., stigmatization, impact on occupational or insurance status, and unnecessary psychological and/or pharmacological treatment for false-positive screens, but the prevalence and impact of these risks have not been rigorously evaluated.

Given the balance of evidence for benefits and minimal evidence for harms, CANMAT continues to recommend depression screening, using a validated scale, in primary and secondary care settings for patients who have risk factors (Table 1.1), provided there are resources and systems available for subsequent diagnostic assessment and treatment for those who screen positive 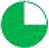 (Table 1.2).

### Q.1.c. What Screening Tools can be Used?

There are many validated scales that can be used for depression screening. The 2-item Patient Health Questionnaire (PHQ-2), comprised of two items from the PHQ-9, is particularly useful in primary care because of its brevity. The PHQ-2 asks about feeling depressed/down/hopeless and experiencing anhedonia/lack of interest over the preceding 2 weeks, rating each answer on a scale of 0 to 3. A positive PHQ-2 (score ≥2) followed by a positive PHQ-9 (score ≥10) has similar sensitivity and better specificity compared to the PHQ-9 alone.

The PHQ is available in many languages and digital formats and can be used for subsequent monitoring during measurement-based care (MBC; see Q.5.a). CANMAT therefore recommends using the PHQ-2 followed, if positive, by the PHQ-9 for depression screening 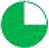 (Table 1.2). While the use of screening questionnaires can help detect clinically significant depressive symptoms, they are not diagnostic and positive screens should be followed by a full diagnostic assessment.

### Q.1.d. What Factors are Important in Assessment and Diagnosis?

**Figure 1.1.**
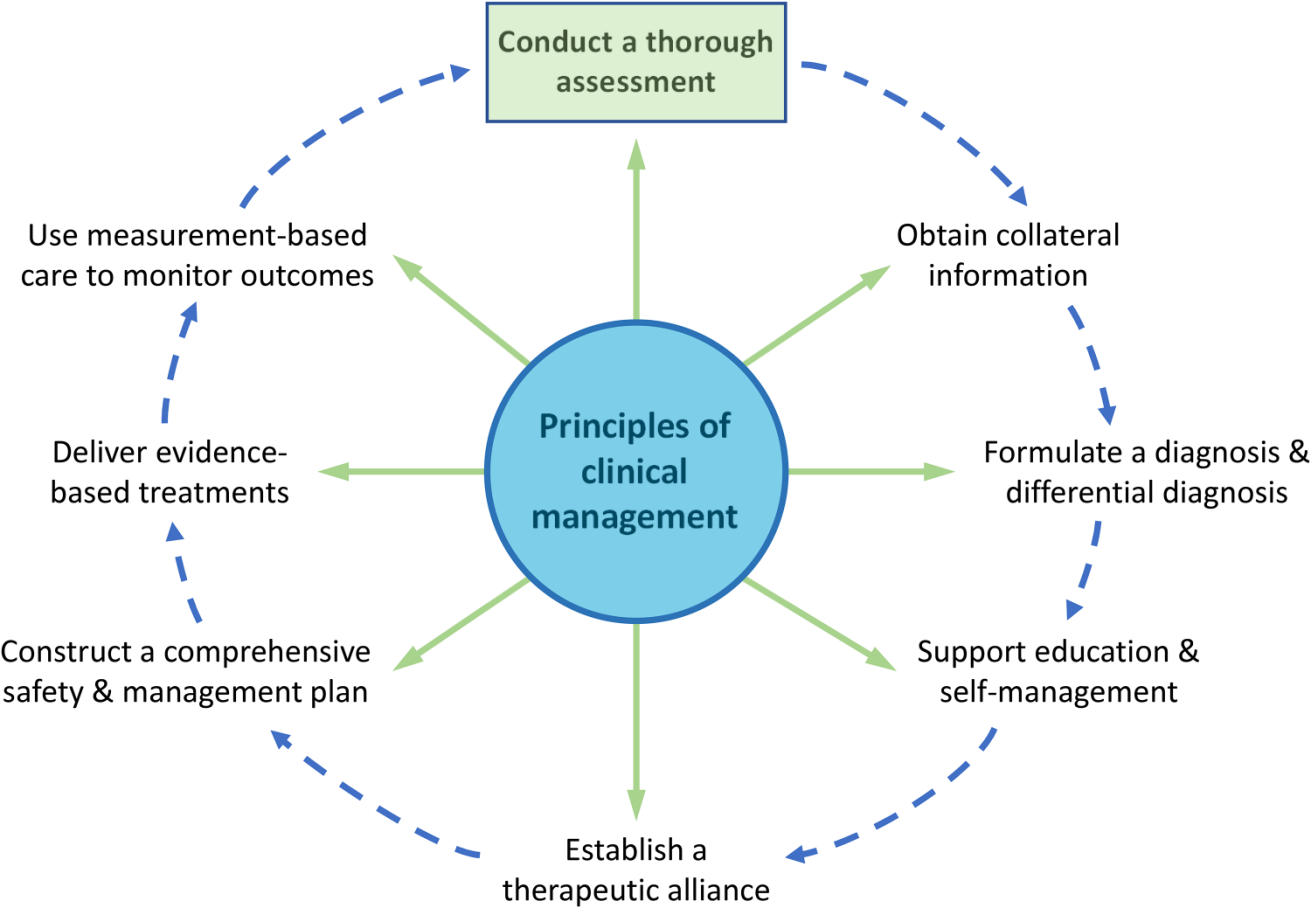
Principles of clinical assessment and management of major depressive disorder.

A comprehensive diagnostic assessment is the first step in the clinical management of MDD 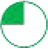
(Figure 1.1). Since MDD is a heterogeneous condition with a myriad of symptoms and presentations, a personalized assessment incorporating a diagnostic framework, such as DSM-5 or International Classification of Diseases-11 (ICD-11), allows exploration of individual differences. A comprehensive assessment should assess emotional, cognitive, and somatic symptoms, as well as vulnerability and resiliency factors across the life span, supplemented by collateral information whenever possible, and recognize ethnocultural diversity (Table 1.2). A history of childhood maltreatment and/or stressful/traumatic experiences is not always volunteered, so these should be explored sensitively during an assessment. The differential diagnosis should include psychiatric and nonpsychiatric medical conditions that present with depressive symptoms, while recognizing that these conditions may also be comorbid with MDD.

The first step is to conduct a thorough assessment of biological, psychological, and social factors, using collateral information when available, to formulate a diagnosis and differential diagnosis. Other management principles are discussed in subsequent questions (Q.2, Q.3, Q.5). Recommendations for principles of care are generally based on Level 3 and Level 4 evidence.

Given the close relationships between MDD and other medical illnesses, a medical workup (including a detailed medical history and targeted physical examination) is important to rule out nonpsychiatric medical conditions. Although there is little direct evidence to support routine bloodwork, a complete blood count (CBC) and thyroid stimulating hormone (TSH) are low-cost blood tests that can help rule out anaemia and thyroid dysfunction as treatable causes of low mood and energy 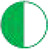. Other laboratory tests should be considered only when history and examination suggest other medical conditions in the differential diagnosis (e.g., testosterone or vitamin D deficiency) or in patients with other medical comorbidities, substance use disorders, older adults, or individuals with self-neglect and impaired judgment or communication 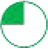.

Similarly, there is no supportive evidence for routine electrocardiography (ECG), electroencephalography (EEG), or neuroimaging in patients presenting with MDD. These diagnostic tests should only be ordered when there are clinical indications 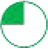. For instance, ECG should be considered in patients with cardiovascular diseases when using medications that potentially prolong QTc interval 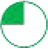. Neuroimaging should be considered in the presence of neurologic signs, new onset or persistent cognitive impairment, or sudden changes in mood, behaviour, or personality 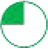. In addition, neuroimaging may be recommended for patients with a new onset of late-life depression to rule out cerebral events or the onset of other structural abnormalities (e.g., tumour, metastasis) 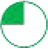.

### Q.1.e. What are Diagnostic Specifiers and Symptom Dimensions?

The Diagnostic and Statistical Manual, 5th edition, Text Revision (DSM-5-TR) includes subtypes within an MDE. These are classified as episode specifiers, based on cross-sectional symptoms of an MDE, and course specifiers, based on the longitudinal pattern of MDEs. Several episode specifiers (melancholic, atypical, psychotic, and catatonic features) are held over from the Diagnostic and Statistical Manual, 4th edition, Text Revision (DSM-IV-TR), while others (anxious distress and mixed features) are new in DSM-5-TR. Course specifiers include seasonal pattern (with primarily a winter pattern of MDEs) and peripartum onset. Specifiers are primarily useful for describing symptom and course patterns, with some utility for prognosis. However, they generally have limited value for selecting treatment (except for psychotic features and seasonal pattern, see Q.3.g and Q.3.m). Other clinical dimensions not identified in DSM-5-TR may be more useful for guiding treatment selection, including cognitive dysfunction, sleep disturbance, somatic symptoms (e.g., pain, energy, and fatigue), and anhedonia (see Q.3.f).

Finally, about 20% to 30% of individuals with MDEs have a chronic course lasting more than 2 years. In DSM-5-TR, a chronic MDE (with full criteria) is classified as a specifier of persistent depressive disorder (PDD), which is characterized by 2 or more depressive symptoms that are present for more than 2 years. MDE in partial remission after treatment is a separate PDD specifier. PDD specifiers also include dysthymia, which is a chronic course of subthreshold depressive symptoms that do not meet criteria for MDE. This conflation of chronic MDE, partial remission MDE, and dysthymia makes it difficult to differentiate specific treatments for PDD (see Q.3.f). Regardless, compared to individuals with episodic MDEs, those with PDD show lower response rates to standard treatments and less likelihood of achieving remission. Management for PDD should employ the DTD framework, with the focus of treatment centred on improving quality of life and functioning instead of symptom remission 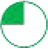 (see Q.7.b).

### Q.1.f. How can Access for Equity Deserving Groups be Improved?

Compared to the general population, the prevalence of MDD is higher in racialized, indigenous, gender minority, and certain religious populations. There is also a recognized lack of access and underutilization of mental health services for equity-deserving groups. The most common barriers to care include costs of treatment, cultural beliefs, stigma, language barriers, fear of being judged by the care provider, unequal geographic distribution of services, and fragmented health services. Some approaches to help overcome these barriers and reduce the existing gap in access to services include screening programs, incorporating culturally competent and collaborative care, and using digital health interventions (DHIs) tailored for equity-deserving groups (Table 1.2).

Screening is underutilized in equity-deserving groups despite the availability of validated screening tools in many different languages and formats. Encouraging and training primary care providers to routinely screen for depression in equity-deserving groups using validated scales (e.g., the PHQ, available in many languages, and Q.1.c) increases access to mental health services, referrals, and treatment. Culturally competent care, in which a patient's sociocultural and language needs are explicitly addressed, is shown to improve psychiatric care and outcomes. Trauma-informed approaches should be used where indicated, e.g., for patients who are Indigenous, to account for the influence of transgenerational and other traumas on assessment and management 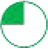. Training mental health professionals in cultural competency, trauma-informed care, healthcare disparities and personal bias also improves care and access for equity-deserving groups. Collaborative care, a healthcare delivery system comprised of case managers alongside primary care providers and mental health specialists (see Q.2.g), may be particularly effective for patients from equity-deserving groups and those from low socioeconomic backgrounds 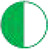.

Finally, telehealth and DHIs (see Q.4) may also provide benefits for equity-deserving groups, as they can address language, stigma, and geographic disparities in service. Systematic reviews have shown that telehealth, delivered in the patient's preferred language with cultural adaptation and cultural matching of patients and providers, improves health outcomes and access to services 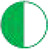. Other studies show that DHIs are promising resources to enhance screening and service delivery. However, challenges with digital literacy and technology costs are barriers to the scale-up of digital services for many patients (see Q.4.b).

## Question 2. What are the Principles for Depression Management?

### Q.2.a. What are the Phases and Objectives of Treatment?

The general objectives of MDD treatment are to achieve symptomatic remission, recover full functioning, restore quality of life, and prevent recurrence, while ensuring patient safety and acceptability of treatments. The specific goals of treatment should be individualized using a patient-centred approach whereby clinicians understand a patient's unique goals and needs, to promote shared decision-making (SDM) and collaboration in determining relevant outcomes (see Q.2.d).

These guidelines retain the 2-phase treatment model (acute and maintenance phases) from the CANMAT 2016 guidelines (Table 2.1). The main objectives of the acute phase of treatment are to address patient safety and select evidence-based treatments to improve symptom severity and functioning. Remission of symptoms, defined as the resolution or near-resolution of symptoms (operationalized using validated rating scales, see Q.5.b), is an important target of acute treatment (see Q.2.b). Specific actions and activities of the acute phase are listed in Table 2.1. Psychoeducation and self-management are integral to MDD management 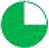. Psychoeducation refers to knowledge and information about the symptoms, coping strategies and treatment options for depression, whereas self-management uses techniques (e.g., problem-solving, lifestyle changes, and behavioural activation [BA]) to empower patients to actively manage their illness and live well. Many resources are available, both in print and online, to support effective self-management for MDD, including the CHOICE-D patient guide.

**Table 2.1.** Summary of Phases of Treatment, Objectives, and Actions.

Phase of treatment	Duration of phase	Objectives	Actions
Acute	Approximately 8–16 weeks, until symptom remission.	Address patient safety.	Assess suicide and safety risks. Define treatment setting (inpatient vs. outpatient). Develop a safety plan.
		Treat to symptom remission and functional improvement.	Establish rapport and therapeutic alliance. Use psychoeducation and self-management. Select and implement evidence-based treatment(s). Monitor tolerability, adherence, response, and side effects.
Maintenance	Approximately 6–24 months following the acute phase (or longer if clinically indicated).	Maintain symptomatic remission.	Make evidence-based adjustments to treatment(s). Address residual symptoms.
		Restore functioning and quality of life to premorbid levels.	Use psychoeducation and self-management. Treat comorbidities. Consider additional psychosocial interventions.
		Prevent recurrence.	Use psychoeducation to identify early symptoms for early intervention. Monitor for long-term side effects and adherence issues. Address barriers to care. Use interventions to promote resilience.
		Consolidate gains during treatment discontinuation.	Discontinue treatments when clinically indicated. Use evidence-based approaches when stopping treatment. Continue treatment when discontinuation is not indicated.

The main objectives of the maintenance phase (Table 2.1) are to consolidate symptom remission, restore both functioning and quality of life to premorbid levels, and prevent recurrence of symptoms (see Q.6). When clinically indicated, acute phase treatments may be tapered and discontinued. Finally, interventions to promote resilience can be used during the maintenance phase to help prevent recurrence.

### Q.2.b. Why is Symptom Remission Important?

Symptom remission remains a crucial clinical target for acute treatment, as substantial evidence supports a lower risk of relapse in those who achieve remission compared with those who do not 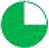. In contrast, failure to achieve remission is associated with continuing symptom burden and poor functional outcomes. Evidence supports symptomatic remission as a realistic goal of acute phase treatment for most patients both in psychiatric and primary care settings. However, in defining remission and recovery, patients often regard aspects of functioning and quality of life as more important than the absence of symptoms. Hence, symptom remission, while important, should be considered only an intermediate objective for optimal treatment outcomes.

It is also worth noting that persistent depressive symptoms are common even in individuals who otherwise achieve symptom remission. These “residual” symptoms should be identified and treated because persisting symptoms represent a major risk factor for recurrence (see Q.6.c). Isolated, mild, and intermittent symptoms can be treated using a symptom-targeted approach with a focus on patient comfort and functionality (e.g., using a short-term medication for insomnia).

Finally, some patients, such as those with PDD, may not achieve symptom remission despite treatment with several evidence-based approaches. For these patients with DTD, the therapeutic focus should shift away from symptom remission towards prioritizing the best possible improvement in functioning and quality of life (see Q.7.b).

### Q.2.c. How are Suicide and Safety Risks Managed?

The risk of suicide attempts in individuals with MDD is 5-fold higher than in the general population. In Canada, there are about 15 deaths by suicide for every 100,000 males, compared to about 5 deaths for every 100,000 females, representing over 4000 suicide deaths per year. Of those who die by suicide, about half suffer from MDD, the most prevalent mental illness in these individuals. Hence, assessing suicide risk and developing and implementing a tailored safety plan are priorities for risk management in MDD.

Suicide risk assessment should be a routine part of psychiatric interviews, especially in emergency room settings, with the aim of making patients feel genuinely listened to and validated. The focus of the risk assessment in the psychiatric interview and subsequent risk formulation should be to understand the basis for suicidal ideation/behaviour, personal strengths and protective factors that can be leveraged for safety planning, and foreseeable changes (Table 2.2). The assessment should identify potentially modifiable risk factors (Table 2.2) because historical and demographic factors which are associated with overrepresentation in deaths by suicide (e.g., male sex, older age, family history of suicide, etc.) are not helpful for risk assessment. Validated suicide risk scales are available but they have low sensitivity and predictive power for suicide behaviours and should only be used alongside clinician judgment (see Q.5.a).

**Table 2.2.** Potentially Modifiable Risk Factors for Suicide in Major Depressive Disorder (MDD).

Potentially modifiable factors associated with higher suicide risk
*Symptoms and life events* Suicidal ideation with a well-developed plan and/or intent to act Hopelessness Anxiety Impulsivity Psychotic symptoms Stressful life events (e.g., financial stress and victimization)
*Comorbid conditions* Posttraumatic stress disorder Substance use disorders (especially alcohol use disorder) Comorbid personality disorders (especially cluster B personality disorders) Sleep disorders Chronic painful medical conditions (e.g., migraine headaches and arthritis)

Management of suicide risk should focus on individualized care, therapeutic risk management, and evidence-based safety planning 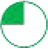 (Table 2.3). For patients at high risk (e.g., experiencing suicidal ideation with intent to act and/or psychotic symptoms), treatment in an inpatient setting is often warranted. The goals of care should include monitoring the severity of suicidal ideation, timely pharmacological and nonpharmacological treatments, modifying the patient's environment and circumstances (e.g., removing lethal means from the home and bolstering support in the community) and ensuring regular follow-up. The month after both initiating and stopping an antidepressant are periods of increased risk for suicide, which require enhanced surveillance and safety planning (see Q.3.g). In Canada and the United States, the 9-8-8 telephone number allows rapid access to help by phone or text for people who are suicidal or their family members, or for clinicians seeking guidance to help a patient at risk for suicide.

**Table 2.3.** Suicide Safety Plan (Adapted from Hawton et al., 2022).

**Step 1.** Identify warning signs – e.g., thoughts, feelings, and circumstances. **Step 2.** Enlist personalized coping strategies – e.g., self-management. **Step 3.** Enable distraction and connect with people – e.g., go for a walk with a friend, go to a cafe or movie, use support groups or online forums. **Step 4.** Engage with social and community supports – e.g., involve personal contacts in contingency plans. **Step 5.** Identify professional contacts – e.g., crisis lines (9-8-8 in Canada and USA), mental health providers, urgent care clinics, and hospital emergency rooms. **Step 6.** Make the environment safe – e.g., remove excess medications, firearms, sharp objects, or ropes; avoid busy streets and heights.

### Q.2.d. What is the Role of Patient Preference?

Patient preference plays a key role in depression management. SDM helps establish a strong therapeutic alliance which facilitates psychoeducation about critical aspects of the treatment, including achieving symptom remission and promoting long-term mental wellness. Clinicians should ensure that patients are aware of the broad range of treatment options available for MDD, including lifestyle changes and self-management, psychotherapy, pharmacotherapy, neuromodulation, and complementary and alternative medicine (CAM) treatments. Clinicians also should explain how treatments work and dispel myths and stigma (e.g., related to medications or role of stress). CANMAT has created a patient and family guide to depression treatment (CHOICE-D) as a psychoeducational aid, available for free download at www.CANMAT.org.

Discussions with patients should also acknowledge the local realities of the healthcare ecosystem, including any limitations to accessing evidence-based psychological and neuromodulation treatments. Moreover, every effort should be made to provide treatments preferred by patients. Given the many evidence-based options, it is advisable for clinicians and patients to agree upon a shared treatment plan with an explanation as to why a specific treatment is recommended, and to offer one or more alternative options so that patients can select and adhere to the care plan that aligns with their values and needs.

In the SDM model, the clinician and patient contribute mutually to the decision-making process. Meta-analyses find that, while SDM may not significantly improve treatment adherence or symptom change, it increases patient knowledge, communication, and satisfaction. CANMAT recommends introducing elements of SDM to improve the care experience for people with depression 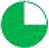. Patient preference should especially be considered with other clinical factors when selecting treatment options, including psychological and pharmacological interventions.

### Q.2.e. How Does Treatment Cost and Access Influence Management?

Treatment costs and health insurance coverage may significantly influence treatment decisions for patients. Publicly funded health care systems may offer only limited or no access to certain evidence-based treatments for depression, especially psychological, neuromodulation, and infusion treatments. Access to these treatments may be available in private health care settings, but cost may be a significant barrier for many patients. Cost may also play a role in medication treatments, given that, in general, newer medications are more expensive than those for which generic formulations are available. When formulating a treatment plan with patients, clinicians should be familiar with the costs of treatments and their patient's insurance coverage and ability to pay, if needed 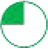.

### Q.2.f. What Lifestyle Interventions are Effective?

Different lifestyle factors have been implicated in the risk of both developing MDD and worsening symptom severity. For instance, insomnia can raise the likelihood of depressive episodes and is also a common residual symptom. Similarly, the relationship between cigarette smoking and depressive symptoms may be bidirectional, with individuals using smoking to alleviate depressive symptoms (self-medication) while smoking, in turn, may increase susceptibility to depressive symptoms. Lifestyle modifications such as exercise or an increase in physical activity, a healthier diet, smoking cessation, and sleep hygiene are beneficial in MDEs (Table 2.4).

**Table 2.4.**
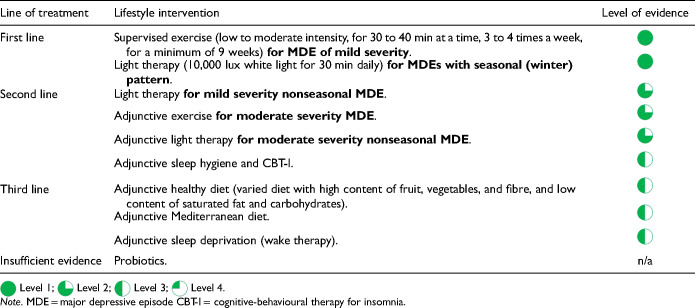
Summary Recommendations for Lifestyle Interventions.

The CANMAT 2016 guidelines noted substantial evidence for the efficacy of exercise as a treatment for MDD 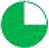. While recognizing heterogeneity in RCT methods, recent meta-analyses continue to show that adjunctive exercise is superior to no treatment and treatment as usual conditions in MDD, with medium to large effects 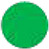. Exercise can also reduce suicidal ideation and has good effects when combined with antidepressants. Meta-analyses show benefits in reducing depressive symptoms with the supervised aerobic activity of moderate to high intensity in adolescents and young adults, with low to moderate intensity exercise in midlife and older women, and with high-intensity interval training in adults. Exercise also provides positive health benefits beyond its effects on depression. Taken together, these findings continue to support the use of supervised exercise (i.e., low to moderate intensity, for 30 to 40 min at a time, 3 to 4 times a week, for a minimum of 9 weeks) as a first-line monotherapy for mild depression and a second-line adjunctive treatment for moderate severity illness 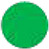 (Tables 2.4 and 3.7).

Maintaining healthy sleep and circadian rhythms are important lifestyle issues. There is a bidirectional relationship between sleep and depression, whereby sleep disturbances (insomnia and hypersomnia) are common symptoms of MDEs but disturbed sleep also increases the risk of developing MDD. Hence, restoring restful sleep is an important objective in MDD treatment and prevention. Although there is limited clinical trial evidence, instructing patients on sleep hygiene is likely beneficial 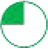. Small-sample RCTs also find that a variant of cognitive-behavioural therapy (CBT) tailored to address insomnia (CBT-I) shows promising effects in MDD 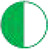.

In contrast to improving sleep, sleep deprivation (also called wake therapy) has long been described as having a rapid but transient antidepressant effect in MDD. While open-label studies show the benefit of sleep deprivation (both total and partial) as an adjunctive treatment, a recent meta-analysis found no benefit for studies where treatments that included sleep deprivation were compared to the same treatment without sleep deprivation 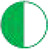. Hence, sleep deprivation remains a third-line recommendation for MDD (Table 2.4). One caution with overnight sleep deprivation is that it may precipitate hypomanic episodes in some susceptible individuals not previously diagnosed with bipolar disorder.

Exposure to natural light is important for maintaining circadian rhythmicity. Light therapy is a chronobiological intervention in which a device delivers timed exposure to supplemental bright light. A typical protocol for light therapy uses 10,000 lux fluorescent white light for 30 min daily in the morning, shortly after awakening. Light therapy continues to be recommended as first-line monotherapy for seasonal (winter) depression (seasonal pattern course specifier in DSM-5-TR) with several consistent supportive meta-analyses 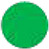. Despite some heterogeneity, recent meta-analyses also find efficacy for light therapy in nonseasonal MDD 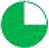, with larger effects when light therapy is combined with antidepressants. This evidence places bright light therapy as a second-line treatment for mild severity nonseasonal MDD and as adjunctive treatment with medications for moderate severity nonseasonal MDD (Tables 2.4 and 3.7).

Regarding diet, many observational studies support the association of an unhealthy diet (high content of refined carbohydrates and saturated fat, and low content of fruits and vegetables, commonly called the Western diet) with increased prevalence and severity of depressive symptoms 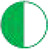. In contrast, there are few interventional studies of diet manipulation. There is only 1 RCT showing a modest effect of a Mediterranean-type diet in adults with MDD, supporting a third-line recommendation 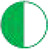 (Table 2.4). With regard to newer interventions such as probiotics, current evidence is equivocal, neither supporting nor refuting beneficial effects. Hence, there is insufficient evidence to recommend probiotics in the treatment of MDD.

### Q.2.g. Who Delivers and Monitors Care?

Several service delivery models have evidence of benefit for the management of MDD, including stepped care, stratified care, and collaborative care. Each of these models emphasizes evidence-based treatments, but the mode of treatment selection and delivery differs for each framework. Regardless of the model, when 2 or more clinicians are involved in a patient's care, ensuring timely and effective communication is essential to maintain continuity of care.

Stepped-care delivery refers to a care model in which patients access sequential treatments of increasing intensity as needed, according to prespecified criteria such as severity of illness. Patients usually enter at the least intensive “step” shown to be effective for their illness and move up or down depending on symptom progression or response. For example, people with mild severity MDEs may be appropriate for lower-intensity interventions while those with more severe and high-risk episodes are streamed to start with higher-intensity programs. In contrast to stepped care, stratified care refers to models in which patients are initially matched to receive treatments customized to both the severity and complexity of their needs. Both models are efficacious and cost-effective in managing depression 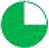.

Collaborative care models are primary care frameworks that emphasize multidisciplinary involvement with 2 or more health professionals. Collaborative care (also variously called shared care and integrated care) is effective in reducing depressive symptoms and suicidal ideation in primary care settings, especially in patients with comorbid medical conditions and from equity-deserving groups, including those from low socioeconomic backgrounds 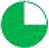.

## Question 3. How are Treatments Selected?

### Q.3.a. How is the Initial Treatment Selected?

The choice of initial treatment for MDD is an important and consequential decision that should be made collaboratively between clinician and patient. Information sharing should include the range of potential treatments, the evidence supporting each, the nature and severity of depression, and the personal situation, expectations, and preferences of the patient. CANMAT recommendations are based primarily on the severity of depression (see *Conventions* section for definitions of mild, moderate, and severe), but other factors, including treatment response in past episodes, patient preference and treatment availability, should also be considered (Table 3.1).

**Table 3.1.**
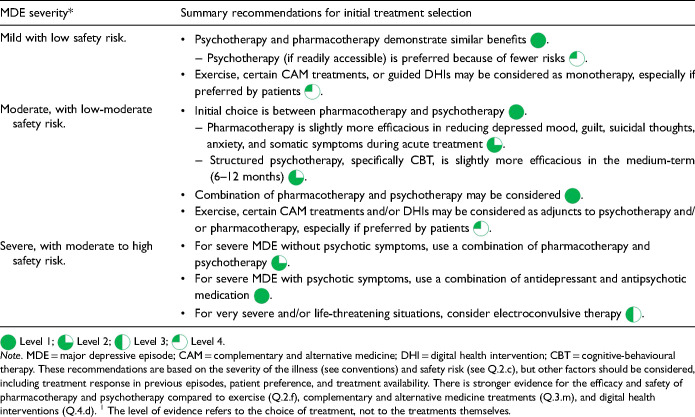
Summary Recommendations for Selecting the Initial Treatment.^1^

For MDE with mild severity and low safety risk (see Q.2c), evidence-based psychotherapy and pharmacotherapy have similar efficacy, but the balance of benefits and risks suggests a first-line psychotherapy, if readily accessible, as the preferred treatment. At this lower level of severity, monotherapy treatment with exercise, CAM (Q.3.m) treatments and guided DHIs (Q.4.d) may be considered, especially when preferred by patients. Note that there is stronger evidence for efficacy and safety of pharmacotherapy and psychotherapy, compared to these treatments.

For MDE of moderate severity with a low–moderate safety risk, the initial choice is between structured psychotherapy and antidepressant medication, the 2 treatment modalities with the most robust and consistent evidence for safety and efficacy 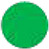. The choice between the two is less straightforward and depends on context, availability, and patient preference. Structured psychotherapies and first-line antidepressant medications are equally effective in treating MDD in the short term. Antidepressants may be slightly more efficacious in reducing symptoms of depressed mood, guilt, suicidal thoughts, anxiety, and somatic symptoms during the acute treatment phase, but structured psychotherapy (specifically, CBT), is more efficacious than antidepressants at 6- to 12-month follow-up. The combination of antidepressant medication and psychotherapy should also be considered 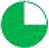. Finally, exercise, certain CAM treatments, and DHIs can also be used as adjunctive treatments to pharmacotherapy and/or psychotherapy, especially when preferred by the patient.

In severe cases of MDE with high safety risk and without psychotic features, CANMAT recommends the combination of antidepressant medication and psychotherapy, either started at the same time or in a staggered fashion as planned sequential treatment 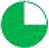 (see Q.3.b). For severe MDE with psychotic symptoms, the combination of an antidepressant and an atypical antipsychotic (serotonin-dopamine activity modulator) is recommended; structured psychotherapy should not be considered until psychotic symptoms subside 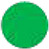. In the most severe MDEs and/or in life-threatening situations (e.g., severe suicide risk and physical deterioration), electroconvulsive therapy (ECT) should be considered as the first-choice option 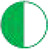.

### Q.3.b. When Should Pharmacotherapy and Psychotherapy be Combined?

Combining psychological treatment with pharmacotherapy is more effective than either alone for acute treatment; combined treatment also is associated with a reduced risk of recurrence. The strongest evidence supports in-person CBT (including mindfulness-based cognitive therapy [MBCT]) initiated sequentially after treatment with an antidepressant is established 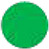. Psychological treatments may also address residual symptoms of depression that remain after pharmacotherapy treatment. A smaller body of evidence supports adding psychodynamic psychotherapy to an antidepressant 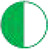. Adding psychological treatment is effective whether the antidepressant is continued or not, but the highest chances of sustained recovery are seen when an antidepressant is continued with added psychological treatment 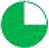. Hence, for patients with mild or moderate severity episodes treated with sequential psychotherapy, the risks and benefits of continuing or tapering medications should be considered (see Q.6.d).

Planned sequential treatment (adding psychotherapy after initial response to pharmacotherapy) may be especially useful for individuals with recurrent and severe forms of depression who carry a high risk of relapse following a monotherapy treatment 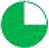 (see Q.6.b). In these cases, pharmacotherapy should be continued during psychological treatment.

### Q.3.c. How is a Psychological Treatment Selected?

Several factors should be considered when choosing an initial psychotherapy, including efficacy, availability, and patient preference. Notably, the following recommendations reflect the evidence from controlled studies, where trained and supervised therapists ensure high treatment fidelity; the evidence does not support the eclectic use of components of the different psychotherapies outlined below.

Consistent with the CANMAT 2016 guidelines, recent evidence continues to support CBT, interpersonal therapy (IPT), and BA as first-line recommended psychological treatments for the acute treatment of MDD 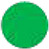 (Table 3.2). Notably, CBT appears to be efficacious across formats including individual, group, and telephone/digital delivery 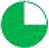.

**Table 3.2.**
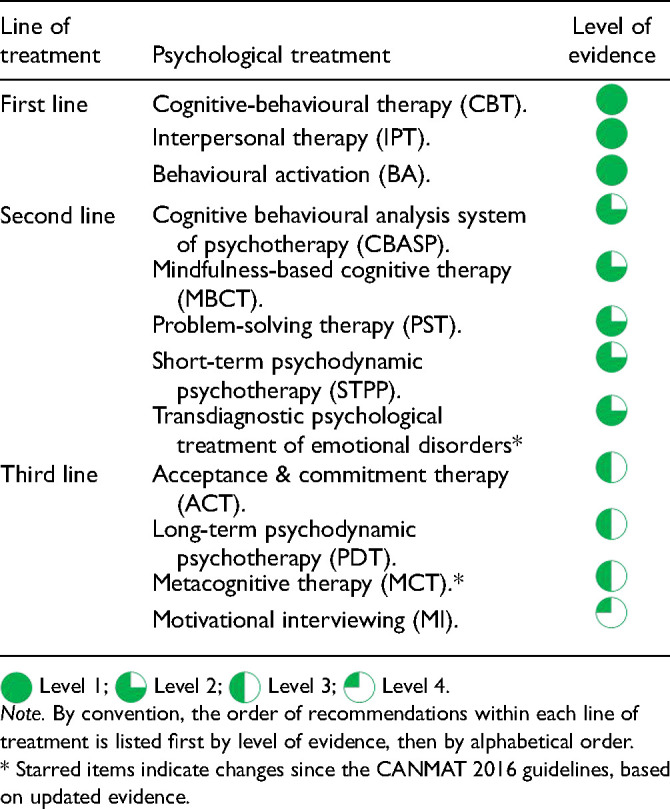
Summary Recommendations for Psychological Treatments.

Meta-analytic comparisons of different psychotherapies are difficult to interpret due to methodological challenges, including lack of blinding and the effects of allegiance to a particular model. A network meta-analysis ranked CBT, IPT, and BA as the top psychotherapies based on outcome measures reflecting treatment response and acceptability. Other psychotherapies were supported by less consistent evidence and are recommended as second-line and third-line treatments (see Q.3.f).

Psychotherapy is similarly effective across most demographic factors, including sex, age, level of education, culture, and ethnicity, hence these patient factors are not informative for choosing the initial psychotherapy. As for clinical factors, illness severity primarily affects the urgency with which the treatment must begin; the more severe the illness, the more imperative it is to start treatment as soon as possible to avert the risk of harm. The severity of MDE does not predict outcomes in CBT versus pharmacotherapy; however, the combination of psychological and pharmacological treatment is more effective than either alone and should be considered in severe cases (see Q.3.b). Importantly, meta-analytic evidence suggests that CBT reduces suicide attempts by half in those who attempted suicide in the previous 6 months; therefore, the addition or initiation of CBT may be considered for people with a recent history of a suicide attempt 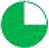.

### Q.3.d. How Many Sessions of Psychological Treatment are Required?

Most improvement occurs during the early sessions of psychological treatments, with diminishing gains thereafter. However, the relationship between the number of treatment sessions attended and the degree of therapeutic improvement remains unclear. Therefore, optimal doses of psychotherapy have been proposed based on the number of sessions in which at least 50% of patients respond to treatment, owing to the decreasing probability of improvement after this cut-off. The strongest evidence suggests that an optimal dose for a first-line psychotherapy, for most patients, is 12 to 16 sessions 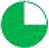.

The frequency of treatment sessions is also related to patient benefit, with increased frequency of psychological treatment sessions producing better outcomes. Twice-weekly sessions for CBT and IPT for depression show improved outcomes compared to once-weekly sessions 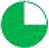. There is little support for the efficacy of psychological treatment delivered less than once per week.

### Q.3.e. How is a Pharmacological Treatment Selected?

When starting pharmacotherapy for MDD, the prescriber has a choice of 31 antidepressants, including 17 that are considered first-line treatments because of robust evidence supporting their safety and efficacy in placebo-controlled RCTs 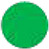 (Table 3.3). A network meta-analysis of placebo-controlled and active comparator trials confirmed that each of these antidepressants was more effective than placebo (Cipriani et al. 2018, in the Key References). The first choice of an antidepressant can be any of the first-line antidepressants, taking into account efficacy, potential for adverse effects, clinical presentation, cost, and patient preference. Clinicians should use psychoeducation when prescribing medications, including discussing risks and benefits, the time course of effect, and dispelling myths and misbeliefs about medication use.

**Table 3.3.**
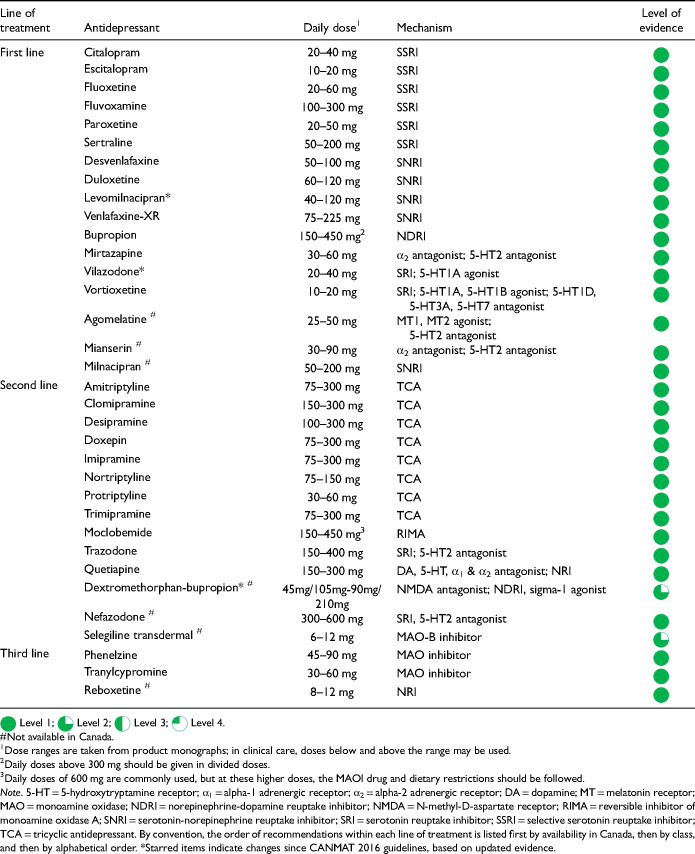
Summary Recommendations for Antidepressants.

All antidepressants have the potential to cause adverse effects. The side effect profiles vary across individual antidepressants, with some differences between classes of antidepressants. For example, frequently reported adverse effects for selective serotonin reuptake inhibitors (SSRIs) and serotonin-norepinephrine reuptake inhibitors (SNRIs) are gastrointestinal and sexual, while for tricyclic antidepressants (TCAs) they are anticholinergic effects such as dry mouth and constipation. Table 3.4 lists rates of adverse events for first-line antidepressants, according to product monographs that summarize results from clinical trial data (note that the rates are not adjusted for placebo rates of adverse effects). Certain important side effects (sedation, weight gain, and sexual dysfunction) are not included in Table 3.4 because they are not adequately assessed in short-term clinical trials, hence, these are separately considered in Table 3.5. CANMAT recommends that clinicians inform patients about potential side effects before prescribing and actively inquire about side effects within 2 weeks of starting an antidepressant 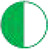.

**Table 3.4.**
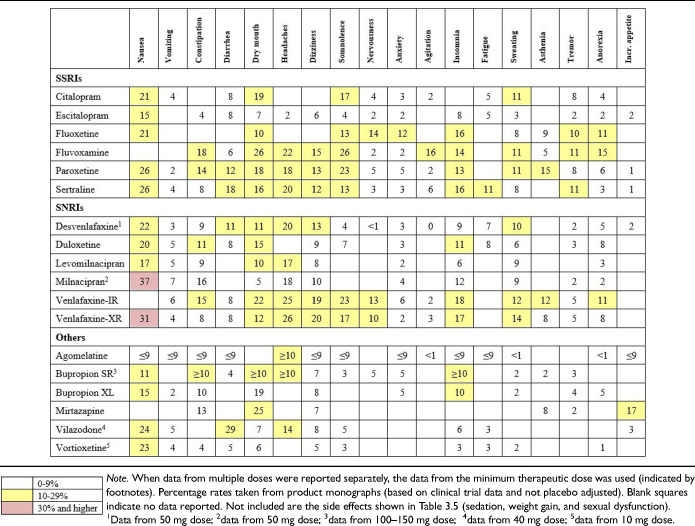
Frequency of Adverse Effects of First-Line Antidepressants.

**Table 3.5.**
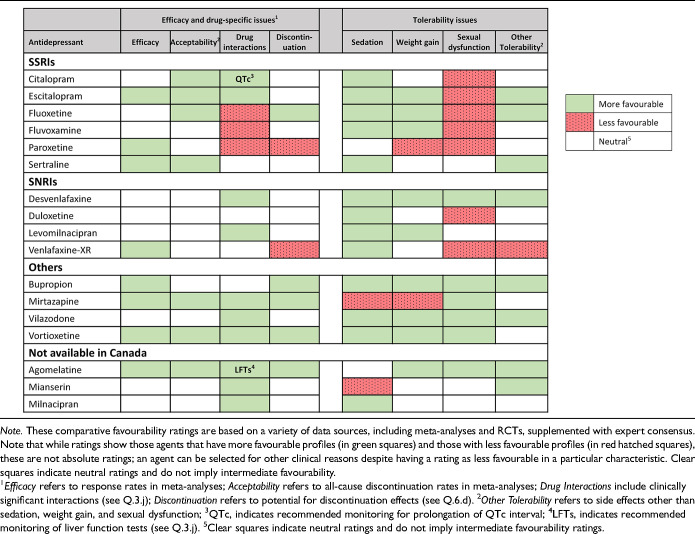
Summary of Comparative Favourability Ratings for First-Line Antidepressants: Efficacy, Acceptability, Drug Interactions, Discontinuation Effects, and Tolerability Issues.

For most adults with MDD, the choice of the first antidepressant is based on the balance of efficacy and tolerability. The previously cited network meta-analysis allows a comparison of differences in efficacy (as defined by response rate) and acceptability (a proxy for tolerability, defined as discontinuation from the study for all causes) among many treatments (Cipriani et al. 2018, in the Key References). In this network meta-analysis, agomelatine, escitalopram, and vortioxetine showed advantages over other antidepressants when considering both efficacy and acceptability 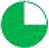.

However, these comparative differences among antidepressants are small and other data sources should be included when comparing efficacy, tolerability and other important characteristics. Table 3.5 summarizes comparative ratings for the first-line antidepressants in categories of efficacy, acceptability, tolerability, drug interactions (see Q.3.j), and potential discontinuation effects (see Q.6.d). These comparative ratings are based on various data sources including meta-analyses and RCTs, supplemented by expert consensus from the CANMAT authors. Note that while ratings show those agents that have more favourable profiles and those with less favourable profiles, these are not absolute ratings; an agent can be selected for other clinical reasons despite having a rating as less favourable on a particular characteristic.

Regarding efficacy, 8 antidepressant medications (bupropion, escitalopram, mirtazapine, paroxetine, sertraline, venlafaxine-XR, vortioxetine, and agomelatine) have evidence for superior response, although these are in the range of 5 to 10 percentage points difference versus comparator medications. Acceptability (all-cause discontinuation rates in meta-analyses) is also more favourable for several medications. For drug interactions (see Q.3.j), fluoxetine, fluvoxamine and paroxetine have clinically relevant potential interactions with other medications. Citalopram carries a risk of QTc prolongation; however, the clinical risk is low, and citalopram has been used safely in large trials of MDD with comorbid cardiovascular disease (note that escitalopram does not carry a clinically relevant higher risk of QTc prolongation). Most antidepressants have a risk of elevation of liver function tests (LFTs, see Q.3.j), but agomelatine includes the requirement for regular monitoring of LFTs in its product monograph.

Table 3.5 also summarizes comparative ratings for side effects of particular importance to patients (sedation, weight gain, and sexual dysfunction) alongside a category of other tolerability (side effects other than those 3). For sedation and weight gain, results from longer-term observational studies are included in the comparative assessment. Of note is that some treatment-emergent weight gain may be normative, e.g., in patients whose depressive symptoms include weight loss. Treatment-emergent sexual dysfunction, which can affect all phases of sexual response (desire, arousal, and orgasm) is also commonly experienced with antidepressants, particularly in those with serotonergic effects. Based on studies where sexual dysfunction is systematically assessed, several antidepressants (desvenlafaxine, bupropion, mirtazapine, vilazodone, vortioxetine, and agomelatine) are associated with lower rates of sexual side effects. Because sexual dysfunction can also be a symptom of depression, an assessment of baseline sexual functioning is important to identify emergent sexual side effects.

In addition to efficacy and tolerability, patient and clinical factors may also be considered in selecting antidepressant medication in some cases, including episode specifiers and symptom dimensions (see Q.3.g). While most antidepressants appear to be effective irrespective of sex, gender, race, ethnicity, and baseline severity, age may be a relevant factor in antidepressant selection. For patients over age 65, SSRIs may be less effective and SNRIs, such as duloxetine, may be more effective 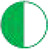. For young patients up to age 25, the balance of efficacy, adverse reactions and risk of discontinuation symptoms may favour using fluoxetine or agomelatine over other antidepressants 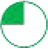.

### Q.3.f. Which Treatments Have New Evidence?

#### Psychological Treatments

First-line psychotherapies (CBT, IPT, and BA) for the acute treatment of depression are supported by new evidence since the CANMAT 2016 guidelines. Most previous second-line psychological treatments continue to build their evidence base, including cognitive behavioural analysis system of psychotherapy (CBASP), mindfulness-based interventions including MBCT, problem-solving therapy (PST), and short-term psychodynamic psychotherapy (STPP) 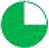 (Table 3.2). Recent meta-analyses support the efficacy of these second-line treatments, while also highlighting limitations such as heterogeneity and risk of bias. Meta-analyses also show that acceptance and commitment therapy (ACT) has efficacy when compared to inactive controls and in those with mild depression, hence, ACT remains a third-line recommendation 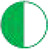.

Two new psychological treatments have accrued enough evidence to be included as second-line or third-line treatments for MDD. First, transdiagnostic or “unified” psychological treatment of emotional disorders, including anxiety and depression, incorporates motivational enhancement, psychoeducation, training in emotional awareness, cognitive restructuring, emotion-driven behaviours (including avoidance), tolerance of physical sensations (including exposure), and relapse prevention. A positive meta-analysis supports its inclusion as a second-line treatment for MDD, which may be particularly useful in the context of concurrent anxiety issues 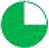 (Table 3.2). Second, metacognitive therapy (MCT) for depression focuses on the awareness and understanding of the thoughts and feelings of oneself as well as others; this treatment is typically goal-directed and enhances metacognitive capacities to gain more flexibility in the attention, monitoring, control, and regulation of cognitive processes. The efficacy evidence in MDD supports MCT as a third-line recommendation 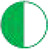.

#### Pharmacological Treatments

A new combination medication, dextromethorphan with low-dose bupropion, was approved in 2022 in the United States of America (USA; not available in Canada), based on clinical trials showing an advantage over placebo and low-dose bupropion alone. Dextromethorphan is a glutamate N-methyl-D-aspartate (NMDA) receptor antagonist, opioid sigma-1 receptor agonist, and SNRI, that is used as a cough suppressant and a common ingredient of cough and cold medications. The addition of bupropion increases the bioavailability of dextromethorphan through inhibition of the cytochrome P450 (CYP) 2D6 liver enzyme; bupropion also is a reuptake inhibitor/releaser of dopamine and norepinephrine. Given the limited evidence, the potential for misuse, and the lack of long-term safety data, the dextromethorphan-bupropion combination is recommended as a second-line treatment 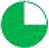 (Table 3.3).

Brexanolone and zuranolone are allopregnanolone agonists that have been approved in the United States for the treatment of postpartum depression. They are not reviewed here because they will be included in the CANMAT guidelines for perinatal mood and anxiety disorders, which are currently in process.

### Q.3.g. How do DSM-5-TR Specifiers and Symptom Dimensions Influence Medication Selection?

In the CANMAT 2016 guidelines, the DSM-5-TR episode specifiers, melancholic features and atypical features, were not associated with specific treatment recommendations. Other specifiers had limited evidence for specific treatments, such as recommendations for benzodiazepines or ECT for catatonic features, and combination antidepressant and antipsychotic treatment, or ECT, for psychotic features. There is no new evidence available to change these recommendations (Table 3.6). Similarly, the DSM-5-TR specifier, with anxious distress, has a prognostic value in that MDE with anxiety is associated with poorer response to standard treatments. However, there is no evidence for better responses with any specific medication, hence all first-line antidepressants are recommended for anxious distress.

**Table 3.6.**
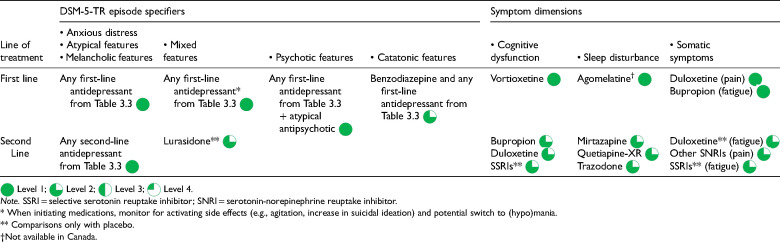
Summary Medication Recommendations for DSM-5-TR Episode Specifiers and Symptom Dimensions.

The DSM-5-TR mixed features specifier describes an MDE presenting with both depressive symptoms and subsyndromal manic symptoms. It may be challenging to differentiate MDD with mixed features from mixed states found in bipolar disorders, hence, patients manifesting mixed symptoms should be managed in consultation with a psychiatrist 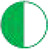. Owing to the short history of the mixed features specifier for MDD, there is minimal RCT evidence for treatment. Given this paucity of data, first-line antidepressants are still recommended for MDE with mixed features (Table 3.6). However, close monitoring for activating side effects (e.g., agitation and increase in suicidal ideation), and potential manic/hypomanic switch, is recommended when initiating antidepressants 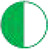. The serotonin-dopamine activity modulator, lurasidone, is recommended as a second-line agent for patients with MDD and mixed features because of an RCT showing efficacy compared to placebo 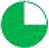.

The 2016 CANMAT guidelines also identified some symptom dimensions not included in DSM-5-TR that may have treatment specificity (Table 3.6). Cognitive symptoms (problems with concentration, memory, and executive functioning) are common in MDD and medications such as vortioxetine, bupropion, and SNRIs may have superior efficacy for cognitive dysfunction compared to SSRIs 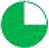. Recent meta-analyses support these findings. Duloxetine and other SNRIs show more benefits for comorbid pain conditions compared to SSRIs. Energy, fatigue, and motivation symptoms may preferentially respond to SNRIs compared to SSRIs 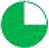.

Other symptom dimensions may be indicators of underlying pathophysiology and relevant for treatment choices. Anhedonia, the reduced ability to experience pleasure, is often clinically expressed as reduced motivational drive and decreased consummatory pleasure. Although there is, as yet, no evidence that any specific agent has superior anti-anhedonia properties compared to other medications, anhedonia is a promising symptom dimension for future clinical trials (Box 3.1).

Box 3.1. Anhedonia as a Treatment Target.

There is significant clinical and research interest in anhedonia as a primary and persisting symptom of MDD (as well as other conditions) and specific treatment approaches. Anhedonia is reported by up to 70% of patients with MDD and its presence is associated with a reduced likelihood of remission with SSRI treatment, possibly because anhedonia may be an indicator of dopaminergic dysfunction. A meta-analysis suggests that monoaminergic antidepressants, ketamine, methylphenidate and psilocybin are associated, in varying degrees, with improvement in the severity of anhedonia. In contrast, escitalopram and riluzole were not as effective in improving anhedonia in MDD. Several investigational medications are in early phase trials to target anhedonia as a primary outcome.

### Q.3.h. Are Antidepressants Associated With an Increased Risk of Suicide?

Collectively, evidence supports the conclusion that antidepressants reduce suicidal thoughts and behaviours, with a greater effect on suicidal ideation than on prevention of suicide attempts and death 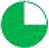. This conclusion, however, does not exclude the possibility that antidepressants may also increase suicidality in a subset of patients. Increased suicide risk may be associated with patient age and the time period of antidepressant use. When examining the risk of suicidal behaviour (death by suicide, attempted suicide, or preparatory acts), a meta-analysis of RCTs found that, compared with placebo, acute treatment with antidepressant medication is associated with increased risk among young adults below age 25, but with a decreased risk of suicidal behaviours among those aged 65 and older; in adults aged 25 to 65, there is no effect 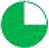. In contrast to these results, a meta-analysis of observational studies found a modest increase in suicidal behaviour associated with antidepressants that were not age-dependent (although there was no increase in a subanalysis of SSRIs only) 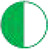. Additionally, suicidal behaviour is noted to be highest in the month before antidepressant initiation (indicating that suicidality predates medication use), remains high in the month after antidepressant initiation, and then declines with continued use of antidepressants 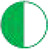. The month after discontinuing an antidepressant also is associated with an elevated risk of suicidal behaviour 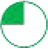.

In summary, the initial few weeks before and after starting any treatment is a higher risk period for suicide. While antidepressants generally reduce suicidal ideation, a small proportion of patients may experience increased suicidal ideation when initiating antidepressants, especially young people. CANMAT recommends routine monitoring of suicide risk for all patients during antidepressant treatment, with enhanced attention during the first 4 weeks following a new antidepressant prescription and after stopping the medication 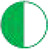. With adolescent and young adult patients, in particular, clinicians should ensure that they are aware of the risk of increased suicidal thoughts and behaviours before initiating an antidepressant. Clinicians also should educate patients that if they experience an increase in suicidal thoughts when starting a new medication, they should (a) understand them as a medication side effect; (b) be aware that increasing suicidal thoughts indicate a need for urgent action to address distress and other symptoms; (c) implement a safety plan (see Q.2.c); and (d) reach out to their care provider, crisis line (e.g., call 9-8-8 in Canada and the USA), or an emergency department 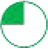.

### Q.3.i. Are There Differences in Formulations of Specific Antidepressants?

Several antidepressants are available in immediate and extended-release preparations. For example, venlafaxine and bupropion are available as extended-release (XR and XL, respectively) preparations that delay time to maximum plasma concentration, reduce their peak concentration, and extend their elimination half-life relative to the immediate-release preparations, making them suitable for once-daily dosing. A meta-analysis found no difference in efficacy between extended-release and immediate-release formulations 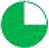.

The availability of generic versions of antidepressants increases access to effective antidepressant treatments at a reduced purchase cost. It should be noted that purchase cost is only a small part of the overall cost of treatment, which includes both direct and indirect costs of care. Generic medications are approved based on demonstrating “bioequivalency” to the reference brand-name product. Differences in bioavailability between generic and brand-name medication are reported, especially for extended-release formulations. However, there are no meta-analyses or clinical trials comparing the efficacy and safety of generic and brand-name antidepressants. Loss of efficacy with a switch between brand-name and generic preparations has been reported, but not systematically studied.

CANMAT recommends using either generic or brand-name antidepressant medications in immediate- or extended-release formulations. When a treatment is effective, the same formulation and/or brand should be continued, and changes should be minimized 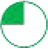.

### Q.3.j. What are the Safety Concerns and Drug Interactions With Antidepressants?

Most first-line antidepressants have a well-established safety record and can be combined with other commonly prescribed medications without significant risk of interactions (summarized in Table 3.4). Here, we highlight exceptions to this rule and cases where caution is warranted.

SSRIs are associated with a modest increased risk of fractures and falls, particularly in older adults. Most SSRIs have few clinically relevant effects on CYP isoenzymes and have low risk for drug interactions. Exceptions are fluoxetine and paroxetine, which are potent inhibitors of CYP2D6, and fluvoxamine, which is a potent inhibitor of CYP1A2, CYP2C19, and CYP3A4. These antidepressants can increase blood levels of concurrent medications that are substrates of these CYP450 isoenzymes. Combining SSRIs with nonsteroidal anti-inflammatory drugs (NSAIDs; e.g., ibuprofen) is associated with an increased risk of gastrointestinal bleeding, which is mitigated with the use of proton pump inhibitors. In addition, concurrent use of SSRIs with diuretics (e.g., hydrochlorothiazide) is associated with an increased risk of hyponatremia, especially in older adults. Mianserin and agomelatine (both not available in Canada or the USA) are the only antidepressants not associated with hyponatremia 7. SNRIs can cause increases in blood pressure and share the risk of interactions with NSAIDs and diuretics noted for SSRIs.

A rare but serious adverse effect of several antidepressants is drug-induced liver injury, which can occur up to 6 months after initiating the medication. Agomelatine, bupropion, duloxetine, and nefazodone are associated with a higher risk of adverse liver effects, while citalopram and escitalopram have a lower risk. Mirtazapine has an excellent short-term safety profile but is associated with significant increases in appetite, weight gain, and long-term metabolic risks.

The safety considerations of antidepressants are partly age-dependent. While SSRIs and SNRIs are generally safe in adults, the extremes of the adult age range are linked to an increased risk of some adverse events. For example, SSRIs and SNRIs are associated with an increased risk of akathisia, agitation, and aggression in young individuals under age 25 years 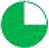. The risks of gastrointestinal bleeding, hyponatremia, liver damage, and QTc prolongation are most prominent in adults over age 65 years 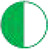.

TCAs are at higher risk of life-threatening effects in overdose, as they can lead to altered mental status, cardiac toxicity, and seizures. SSRIs should be preferentially considered for individuals with a significant risk of overdosing 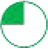. Monoamine oxidase inhibitors (MAOIs) have the potential to cause life-threatening interactions (serotonin syndrome and hypertensive crisis) when combined with medications affecting monoamine metabolism (e.g., dextromethorphan-containing cough syrups, dextromethorphan-bupropion combination, and serotonin reuptake inhibitors), herbal remedies (St John's wort) or food items containing high levels of tyramine (cured meats, mature cheeses, and fermented products such as miso, kimchi, and marmite). Because of these potential interactions, MAOIs are reserved as a third-line option and should be prescribed only after providing detailed guidance on avoiding interacting medications and foods 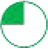.

### Q.3.k. Can Pharmacogenetic Testing Inform Medication Selection?

Several commercially available pharmacogenetic tests (also called pharmacogenomic tests) provide recommendations to inform the selection and dosing of antidepressants to reduce the likelihood of adverse reactions and increase the likelihood of positive responses. Currently, available tests are based on variants in genes that code for enzymes responsible for the metabolism of psychotropic medication, with some tests combining variants in genes coding for serotonin transporter and receptors. Meta-analyses show higher response and remission rates with pharmacogenetic testing compared to usual care, albeit with modest effect sizes, especially in absolute risk reductions (e.g., for response rates, ranging from 4.6 to 7.2 percentage points difference in favour of pharmacogenetic testing) 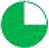. Moreover, the RCTs included in the meta-analyses have significant risks of bias, primarily with a lack of blinding. Hence, CANMAT does not recommend the routine use of pharmacogenetic tests, because the clinical benefits are too modest and inconsistent to justify the delay in treatment associated with obtaining the test results 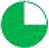. Similarly, routine monitoring of plasma levels of antidepressants is not recommended (except perhaps for TCAs, see Box 7.1) because the relationship between plasma levels and clinical outcomes (response and adverse effects) is weak 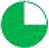.

However, pharmacogenetic testing or plasma level monitoring may be useful in some clinical situations, e.g., when there are severe or unusual adverse effects with low doses of medications, or when there is poor response to therapeutic doses. In these situations, the tests may help identify the small proportion (although this can vary in certain populations) of individuals with atypical metabolizer status and/or unexpected plasma levels 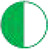. If pharmacogenetic tests are done for these reasons, or if test results are available when done for another medical condition, the Sequence2Script website may facilitate the interpretation of results according to guidelines from the Clinical Pharmacogenetics Implementation Consortium 7.

### Q.3.l. Can Other Biomarkers Inform Treatment Selection?

A biomarker is an objective measure of any substance, structure, or process in the body or its products that may influence or predict a disease, prognosis, or treatment outcome. Biomarkers such as plasma levels of specific proteins, imaging or electrophysiological measures of brain function have been examined as potential indicators to predict which treatment may work for which person. Inflammatory markers in the peripheral blood and electrophysiological measures of brain function have shown consistent associations with antidepressant treatment outcomes. For example, increased levels of interleukin 8 and the C-reactive protein have been associated with worse responses to SSRIs. Because of the small effect size of these associations, CANMAT does not recommend measuring these biomarkers for routine antidepressant selection 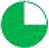.

Other biomarkers with evidence of a reproducible predictive effect include EEG measures of brain activity. For example, stronger loudness dependence of the auditory evoked potentials is associated with a better response to SSRI. Given the early state of evidence, modest effect size, lack of evidence of better response with alternative treatments, and the need for specialized equipment, CANMAT does not recommend routine use of EEG for selecting initial treatment for depression 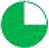.

### Q.3.m. What Complementary and Alternative Medicine Treatments are Effective?

CAM treatments are increasingly used to manage depression. Here we provide recommendations for using the CAM treatments that have the best quality evidence, focusing on new data since the CANMAT 2016 guidelines (see Sarris et al., 2022, for practical guidance/dosage information). To date, no CAM treatment has reached the level of evidence that would make it comparable to a first-line psychotherapy or pharmacotherapy treatment for moderate to severe depression. There is also inconsistency in the therapeutic dose ranges of most CAM treatments. CANMAT therefore recommends the consideration of CAM treatments alone only for MDE of mild severity (see Q.3.a), whereas CAM treatments may be used as adjuncts to standard treatments in moderate severity illness.

Herbal compounds include St. John's wort, which has evidence for efficacy in mild depression 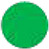. Caution is warranted when combining St. John's wort with serotonergic medications (e.g., SSRIs and SNRIs), as there is an uncertain risk of serotonin syndrome. In addition, St. John's wort is a potent CYP 3A4 inducer with risk for drug interactions, which is an important consideration since it is available as an over-the-counter medication. Although maintenance studies and efficacy data in more severe illnesses are lacking, St. John's wort is recommended as a first-line treatment for MDE of mild severity 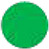 and as a second-line treatment for moderate severity MDE 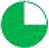 (Table 3.7).

**Table 3.7.**
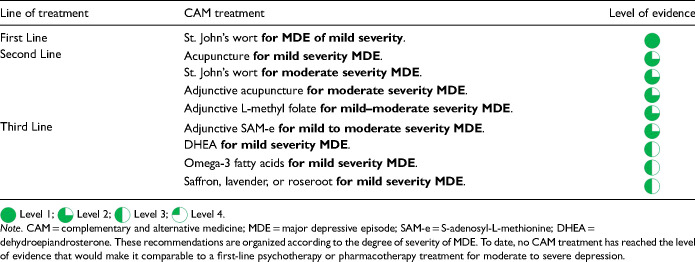
Summary Recommendations for (CAM) Treatments.

Other herbs such as *Crocus sativus* (saffron), *Lavandula* (lavender), and *Rhodiola* (roseroot) have only modest evidence for benefit in MDE. A meta-analysis found the efficacy of saffron for reducing depressive symptoms when compared to placebo and as an adjunct to antidepressants. Another meta-analysis on the efficacy of lavender reported a reduction in depressive symptoms compared to control conditions. However, given the modest benefit and the major limitations of the RCTs, saffron, lavender, and roseroot continue to be recommended as third-line treatments in mild illness 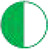 (Table 3.7).

Nutraceuticals are unregulated products which include substances derived from natural sources, including dietary supplements and food additives. L-methyl folate (folic acid) has meta-analytic evidence for efficacy in mild–moderate MDE, but the small effect sizes make it a second-line recommendation 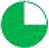 (Table 3.7). S-adenosyl-L-methionine (SAM-e) was previously recommended as a second-line adjunctive treatment for mild to moderate MDE. Owing to mixed findings in recent RCTs, SAM-e is now downgraded to a third-line treatment 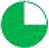. Similarly, omega-3 fatty acids were recommended previously as a second-line intervention for mild-severity episodes. A Cochrane review concluded there was insufficient high-quality evidence to evaluate its effects accurately, hence, omega-3 fatty acids are now downgraded to a third-line treatment 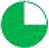. There is modest evidence for the benefit of dehydroepiandrosterone (DHEA) for MDD in a recent meta-analysis, hence DHEA remains recommended as a third-line CAM treatment.

Acupuncture has also been studied in depression, with several meta-analyses showing the efficacy of acupuncture, as monotherapy and as an adjunct to antidepressants, versus all control conditions. However, a Cochrane review noted that, in comparisons of acupuncture with control/sham acupuncture conditions, the effect size was smaller than clinically relevant thresholds. There is insufficient evidence for its use in more severe illness. Hence, acupuncture is recommended as a second-line treatment for mild-severity MDD, and as a second-line adjunctive treatment for moderate-severity MDD 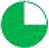 (Table 3.7).

### Q.3.n. How are Comorbid Conditions Managed?

Comorbidity with psychiatric and other medical conditions is common in MDD. For example, high rates of psychiatric comorbidity (10% to 30% for many disorders, e.g., attention-deficit hyperactivity [ADHD] disorder, substance use disorders, and personality disorders; and 40% to 60% for anxiety disorders) are reported in patients with MDD. Similarly, rates of comorbid MDD are high (10% to 30%) in patients with nonpsychiatric medical conditions, e.g., diabetes mellitus, cardiovascular disease, cancer, and chronic pain, among others. The bidirectional nature of the relationships between depression and many medical conditions underscores the importance of recognizing and treating comorbidities with MDD.

A review of treatment for specific comorbid conditions is beyond the scope of these guidelines. Generally, the presence of a comorbid condition makes MDD more difficult to treat, and depressive symptoms may be conflated with symptoms of the other condition. Similarly, it may be difficult to distinguish the side effects of depression treatment from the side effects of concomitant medications. It is important to minimize polypharmacy and check for drug interactions with concomitant medications to avoid iatrogenic adverse effects. The choice of pharmacotherapy should also consider medication side effect profiles that do not worsen comorbid conditions.

For pharmacological treatments, patients with psychiatric and other medical comorbidities have a worse treatment response to antidepressants than those without 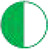. However, antidepressants remain indicated for treating MDD with comorbidities. An umbrella systematic review and meta-analysis of RCTs for MDD comorbid with 27 other medical conditions reported that antidepressants were more efficacious than placebo across a range of medical comorbidities, including myocardial infarction, coronary artery disease, stroke, inflammatory bowel disease, diabetes mellitus, and cancer among others 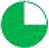.

For psychological treatments, patients with comorbid other medical conditions benefit from psychological treatments similarly to those without 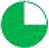. Despite the limitations of heterogeneity in studies, recent meta-analyses support the efficacy of psychological treatments, such as CBT and BA, for specific medical comorbidities, including coronary heart disease and neurological conditions 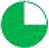.

The CANMAT 2016 guidelines cited evidence that the presence of any personality disorder with MDD reduced the benefit of psychotherapy. Recent evidence from higher-quality studies, focusing on CBT, no longer supports this finding. Hence, CBT (16 to 20 sessions) is recommended for patients with depression and concurrent personality disorder 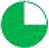. Evidence continues to support the use of CBT for MDD with concurrent anxiety, substance use, or ADHD 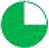. CBT of greater complexity (i.e., including therapeutic components of BA and cognitive restructuring) should be considered with psychiatric comorbidities.

### Q.3.o. How are Cultural and Religious Practices Integrated Into Treatment?

Most research supporting evidence-based psychological treatment for depression relies on samples from dominant cultural groups such as White North Americans and Europeans. However, evidence in support of these treatments in other countries and cultural groups is growing, e.g., CBT for Arabic-speaking adults and mindfulness and acceptance-based interventions for Black Americans 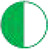. Cultural adaptations may require therapy modifications (e.g., use of culturally appropriate or conceptually equivalent idioms and metaphors), therapist adaptations (e.g., therapist matching, training, and style), and other features such as integration of religion and spirituality, and local remedies and practices. CANMAT recommends cultural and religious adaptations of evidence-based psychotherapies where available and appropriate, based on their demonstrated efficacy across various cultural and religious groups 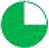.

## Question 4. What is the Role of DHIs?

### Q.4.a. What are DHIs?

Digital health technologies refer broadly to the use of computers, smartphones, and tablet devices for a variety of purposes, including disease screening, monitoring, treatment, and preventing recurrence. The term also encompasses wearables such as activity trackers, digital phenotyping, text or SMS messaging, and videoconferencing for virtual care. For this guideline, we use the term, DHIs, to denote internet programs or mobile apps that deliver depression treatment.

Many DHIs provide tools to promote depression self-management, drawing on evidence-based psychotherapies such as CBT, BA, and mindfulness. Some DHIs specifically address MDD and are designed to supplement clinical care while others aim to be a substitute for in-person care. Other DHIs target nonspecific depression/anxiety symptoms, stress, or burnout, which may indirectly benefit MDD. DHIs are potentially useful for individuals with mild to moderate severity of depressive symptoms; however, symptoms of severe depression, such as poor motivation and cognitive dysfunction, may interfere with an individual's ability to use DHIs.

The potential advantages of DHIs include access to treatment at times and places that are convenient for the user, provision of feedback to the user in real-time, and customization of interventions for specific populations and in different languages, e.g., children and youth, people with nonpsychiatric medical conditions, postpartum users, ethnocultural groups, etc. Hence, DHIs may offer a resource that can complement and build a more engaged and accessible mental health care system.

However, the clinical use of DHIs also poses many challenges. Few DHIs are rigorously evaluated for efficacy and safety. Privacy and security issues are often inadequately safeguarded. User engagement with DHIs decreases over time, e.g., less than one-third of individuals with depression are still using a DHI after 6 weeks. Access to DHIs may also be impeded by the “digital divide” or digital inequity factors, e.g., low digital literacy, inability to purchase a computer or mobile device, and lack of internet access. Hence, addressing the digital divide is important for people with MDD. Mental health clinicians also need training in digital literacy and how to assess, recommend, and use DHIs with their patients.

### Q.4.b. What are Best Practices for Evaluating DHIs?

Many individuals with depression are interested in using DHIs to support their mental health care but they often face challenges when looking for evidence-informed, engaging, and safe DHIs. Commercially available DHIs for depression are often promoted by internet or app store search algorithms, which prioritize popularity and often fail to disclose criteria for efficacy, safety, privacy, and security of personal health information. Positive user reviews may contain anecdotal evidence of utility, but they do not necessarily correlate with expert evaluation. While DHIs developed by academic researchers may be free of commercial bias and have a more robust evidence base, they are seldom publicly available.

Without guidance from clinicians, patients may self-select potentially inappropriate or unsafe DHIs. Table 4.1 summarizes important questions to help clinicians evaluate DHIs for clinical use with their patients. Efficacy is the most important consideration. However, of the tens of thousands of DHIs that are commercially available, few are developed with an evidence-based framework and even fewer have undergone rigorous trials in the targeted population. In the absence of RCTs, other evidence for effectiveness may be informative, e.g., observational studies, studies in nonclinical populations, developer identity (commercial vs. health care), and alignment of features and content with evidence-based therapies.

**Table 4.1.** Considerations for Evaluating Digital Health Interventions (DHIs).

Category	Evaluation questions
Efficacy	Does the DHI use evidence-based techniques? Has the DHI been evaluated in clinical trials in the same patient population? Are there real-world evaluations? Has the DHI been reviewed by credible sources?
Privacy and security	Is there a clear privacy and security policy? Is password protection or authentication included? What personal information does the DHI collect? Can personal data be deleted upon request? Will any personal data be shared or sold to a third party?
Risks	Will using the DHI delay proper assessment and treatment? Is harmful, inaccurate, or inconsistent advice given? For guided DHIs, what is the training and supervision policy for therapists/coaches? Will users feel worse if they are not able to complete the DHI? What is the procedure if users experience a crisis?
Access	Are there upfront or ongoing costs? Are there digital divide challenges? Are there accessibility issues (e.g., language, visual or typing barriers)? How much time does it take to use the DHI? Can reports or summaries be shared with the clinician?

Data privacy and security are paramount for DHIs, but many have unclear or inaccurate privacy/security policies, and data are often shared with or sold to third parties. DHIs are more likely to be trustworthy if they have a clear privacy policy, guarantee to keep data anonymous, offer data security measures including password protection, explicitly state they will not sell data to third parties, and allow users to delete or opt out of data collection entirely. Other privacy risks should also be considered, e.g., risk of unwanted disclosure if a mobile app has a diagnosis in its title. Clinicians should also consult their regulatory bodies for up-to-date guidance on the evaluation and use of DHIs.

There may also be potential harms from the use of mental health DHIs, especially in vulnerable individuals. For example, daily mood monitoring and reminders for depression management may exacerbate depressive symptoms and heighten their salience in individuals with self-stigma or unresolved grief about their diagnosis. DHIs should include clear procedures to follow when user responses indicate severe distress, such as information on crisis lines. DHIs offering peer support may carry risks, such as the exchange of inaccurate or harmful information, interpersonal conflict, and unwanted disclosure of personal information. Some of these risks can be mitigated by features including therapist moderation of support groups, peer-developed content that can be reviewed for accuracy, and the ability to participate anonymously if desired.

Access, cost, and ease of use are also important aspects, particularly for users with disabilities or language/literacy issues. While up-front costs for many DHIs may be low, some have additional costs such as subscriptions for extra features or therapist guidance. Disengagement is commonly experienced during a depressive episode, hence, normalizing and expecting this can reduce shame and allow conversations with patients about compensatory strategies. Clinicians can also ask patients about the current use of other apps to identify features which may support DHI use (e.g., gamification, notifications, and social networking integration). Sharing data with a health care provider or a trusted support may also help the user make sense of changes in mood states over time, as well as detect any adverse events.

Clinicians and patients can also utilize standardized frameworks for DHI evaluation. The nonprofit website Mindtools.io offers star-rating app reviews using the Enlight scale, which considers content and user engagement. The Division of Digital Psychiatry at the Beth Israel Deaconess Medical Center created the M-Health Index and Navigation Database (MIND), a database which allows users to evaluate apps providing specific features for their needs.

### Q.4.c. What is the Difference Between Guided and Unguided DHIs?

Unguided (i.e., self-directed) DHIs are programs in which the user engages independently, without help or contact with another person, whereas guided (or facilitated) DHIs are those where an individual supports the user to guide them through the program. Guides may be clinically trained (e.g., a CBT therapist) or equipped with technical knowledge of the DHI to encourage the user to complete the program (e.g., a lay coach or peer support person). Guidance can be synchronous (advice is given by the guide in real-time while the participant is using the DHI) or asynchronous (advice is provided when it is convenient for the guide to respond, e.g., by email or text). In some cases, guidance involves specific feedback on a user's responses to homework (e.g., a thought record) generated by the DHI. Guides are often helpful to motivate individuals to use and complete the program, thereby enhancing efficacy.

In studies that compare self-directed and guided DHIs, all types of interventions are commonly grouped within a single guidance category of “guided” or “unguided.” This limitation conflates the efficacy of guided versus unguided DHIs with potential differences in efficacy within each group. Nonetheless, in these studies, guided DHIs are generally more effective than self-directed DHIs 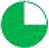.

### Q.4.d. What are Examples of Guided (Facilitated) DHIs?

Guided DHIs offer varying levels of person-facilitated support, ranging from 15  to 60 min per week, by trained therapists, peer supporters, or lay coaches. The format of guidance also varies, from telephone calls to email or web-based messaging. Table 4.2 shows some illustrative examples of facilitated DHIs, based on criteria including availability, popularity, and having at least 1 published evaluation study.

**Table 4.2.** Examples of DHIs for Depression.*

DHI	Description	Comments
**Guided (facilitated) DHIs**		
Good days ahead*	Program designed for use by a CBT therapist to provide additional CBT instruction outside of the session.	Two large RCTs showed this program reduced the need for face-to-face CBT by half, with equivalent clinical outcomes.
BounceBack	Free online CBT program with various formats: self-directed version, or with telephone-delivered lay coaching.	No RCT but a large prospective study was positive in MDD; available free in several Canadian provinces, managed by the Canadian Mental Health Association.
Deprexis*^#^	Online CBT program with tailored guidance provided by an AI conversational agent; includes BA and mindfulness techniques.	Structured 12-week program designed for 90-day use; available in many countries in several languages; tested in multiple clinical trials, with a positive meta-analysis. Available in Europe and the USA.
MoodBeacon	Online guided CBT program; guidance provided by trained therapists.	Although there is no published study, this DHI was reviewed and approved for use by the Province of Ontario in 2021.
Pacifica/Sanvello*^#^	Mobile- and web-based apps intended to assist users in relieving stress, anxiety, and depression. Free; offers in-app purchases.	Uses blend of CBT, mindfulness, and relaxation tools; suitable for adults and adolescents; guidance is through peer discussion in chat groups; has positive results from an RCT. Available in the USA.
**Unguided (self-directed) DHIs**		
Catch It	Free CBT-based mobile app, primarily uses automatic thought records and reflection exercises to address anxiety and depressive symptoms.	Developed jointly by the Universities of Liverpool and Manchester; includes a mood tracker and diary; and has an observational trial of users showing benefits.
Headspace	Popular mobile app with self-guided meditation and mindfulness techniques and some CBT approaches.	Designed for a wide variety of stress, depression and anxiety symptoms; extensively studied with good outcomes in many conditions (of mild severity), but not specifically in MDD.
MoodGYM*	Internet-based CBT self-help program, with very structured modules.	Most widely used platform; initially developed by Australian National University; extensively studied with good outcomes in mild depression.
MoodKit* ^#^	CBT-based toolkit app with detailed instructions on how to use thought records and BA.	Teaches multiple aspects of CBT and is well-integrated with mood monitoring and optional journaling. Available in the USA.
Spark Direct* ^#^	A mobile app providing core elements of CBT and BA in a 5-week program.	Designed for youth depression; uses interactive tasks and rewards; tested in an RCT with positive findings. Available in the USA.

*Note*. AI = artificial intelligence; BA = behavioural activation; CBT = cognitive-behavioural therapy; DHI = digital health intervention; MDD = major depressive disorder; RCT = randomized controlled trial. These DHIs are illustrative examples only and are not officially endorsed by CANMAT. They were selected based on criteria including availability, popularity, and having a published evaluation study. Given the rapid changes in DHIs, they must be individually evaluated prior to clinical application, especially to ensure adequate privacy and security safeguards (see Q.4.b). DHIs available without cost are noted in the description.

*DHI has at least 1 positive RCT in patients with MDD. ^#^ Not available in Canada.

The most-studied guided DHI approach is internet CBT (iCBT). These programs offer guidance from a therapist or are intended for use by clinicians working with their patients. A recent umbrella review of iCBT described medium to large effect sizes in 4 meta-analyses 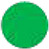. The Good Days Ahead program, with therapist or telephone support, was evaluated in 2 large RCTs with evidence for efficacy. Some iCBT programs (e.g., Deprexis) also incorporate chatbot agents for conversational interaction with users. Hence, guided iCBT is recommended as a first-line monotherapy for mild depression 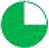, and as a first-line adjunctive treatment for moderate severity MDD 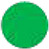 (Table 4.3).

**Table 4.3.**
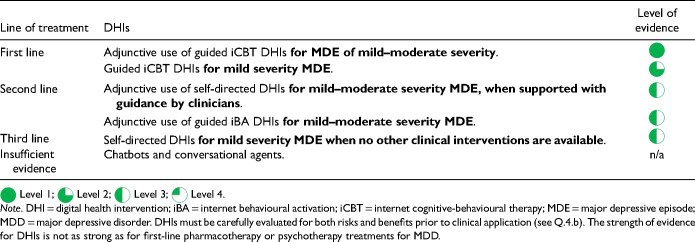
Summary Recommendations for DHIs.

There is also evidence for the efficacy of guided internet BA (iBA). A meta-analysis found that iBA for depression was superior to psychoeducation or treatment as usual, and noninferior to other behavioural therapy and mindfulness formats 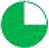. However, 1 RCT of guided email support found no differences compared to a waitlist control condition. Because of this inconsistency, guided iBA is recommended as a second-line adjunctive treatment for mild–moderate MDD when supported by clinicians. Although other guided DHIs based on ACT, dialectical behavioural therapy, and mindfulness are being developed, there is insufficient evidence to recommend them.

### Q.4.e. What are Examples of Unguided (Self-Directed) DHIs?

Prior to the advent of digital interventions, certain self-help books were prescribed in RCTs to have efficacy for MDD. Early DHIs adapted these into self-guided computer- and internet-delivered programs, which were essentially electronic self-help books providing basic psychoeducation and introducing key elements of CBT or BA. An example of this approach is MoodGym, which began in 2001 as a self-directed online course and has subsequently added numerous interactive exercises and questionnaires to engage the user with the learning material.

There is limited evidence of efficacy for most self-directed DHIs. A meta-analysis of 18 RCTs found a moderate positive overall effect on depressive symptoms for smartphone-based DHIs versus control conditions, but there was no significant effect in the 2 RCTs involving participants with a diagnosis of MDD. Given the low level of evidence, CANMAT does not recommend routine use of self-directed DHIs as monotherapy for MDD, except in cases of mild severity when other resources are not available 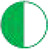 (Table 4.3). Self-directed DHIs may be more useful when clinicians actively encourage patients to use and complete the program, hence, they are recommended as second-line adjunctive tools to usual clinical care when clinicians provide support and guidance 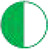. A pragmatic technique for the clinician acting as a guide is to periodically ask their patient if they have utilized the self-directed DHI, and if so, what they are learning.

More recently, some self-directed DHIs have introduced artificial intelligence (AI) to provide interactive feedback and simulate human conversation. These conversational agents, known as chatbots, can be programmed with either predefined responses, usually given by text but sometimes by voice, or free-range responses that are generated by AI algorithms. Chatbots potentially can provide personalized and tailored components of interventions, such as CBT, through interactive conversations while offering a sense of empathy, understanding, and nonjudgmental support. However, the clinical application of chatbots is still at an early stage and little is known about potential risks (such as inappropriate responses) from these AI systems. A recent meta-analysis summarized 4 trials in adults with depressive and anxiety symptoms; while chatbots had significant benefits compared to control conditions, only 1 trial included the diagnosis of MDD and the results were graded as low quality because of the small sample size and high-risk of bias 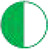. Hence, there is currently insufficient evidence to recommend chatbots and conversational agents for the treatment of MDD.

## Question 5. How is Treatment Monitored?

### Q.5.a. What is Measurement-Based Care?

MBC is an evidence-based practice that uses routine outcome measurement and feedback to guide clinical decisions. Practically, MBC in psychiatry comprises 3 components: (a) regularly using validated outcome scales during patient encounters; (b) reviewing the scale scores with patients; and (c) using the scale scores alongside clinical assessment to support collaborative decision-making.

Several high-quality trials and systematic reviews show that MBC improves medication adherence and outcomes, especially in the pharmacological treatment of MDD 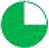. MBC is also useful with psychotherapy, although many of the supporting studies involve mixed-diagnosis outpatient samples and use more general outcome measures, such as the outcome questionnaire. In these studies, MBC was associated with enhanced patient engagement, higher intervention accuracy, and shorter treatment duration 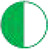. Importantly, MBC can identify nonresponders who might need additional therapeutic techniques or sessions, or who would benefit from alternative treatments. Across treatment modalities, MBC can enhance the therapeutic alliance and facilitate SDM. Patients become active participants in monitoring their treatment, which helps them relate collaboratively with their care providers. For these reasons, the use of MBC is recommended in the management of MDD (Table 5.1).

**Table 5.1.**
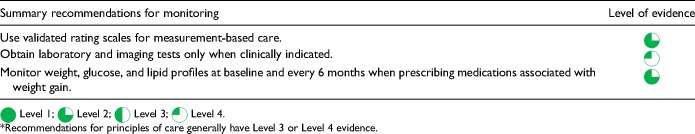
Summary Recommendations for Monitoring Treatment.*

Many validated scales can be utilized for MBC to track various outcomes, e.g., symptoms, side effects, functioning, and quality of life (Table 5.2). For busy clinical settings, patient-rated scales (also called patient-rated outcome measures (PROMs)) are preferred because they are well-correlated with clinician-rated scales but take less time to administer. These PROMs can be supplemented by simple clinician-rated scales. Many are general scales that provide an overall severity measure, such as a total score. More detailed scales can be used to assess specific dimensions of depression, such as anhedonia, sleep, and cognition, when relevant. Some validated scales focus specifically on suicidal ideation and intent, with both clinician-rated (e.g., the Columbia Suicide Severity Rating Scale [C-SSRS]) and patient-rated (e.g., the Suicide Scale) scales available. Some patients find it easier to endorse suicidal thoughts on a questionnaire than face to face with a health practitioner. However, while scales can help assess suicidal ideation, they cannot reliably predict suicide attempts or behaviours. When scales for suicide risk are used, the results should be promptly reviewed and followed up with a clinical assessment if scores indicate risk.

**Table 5.2.** Examples of Validated Rating Scales for Measurement-Based Care.

Outcome	Clinician-rated scales	Patient-rated scales
Symptoms/severity	Hamilton Depression Rating Scale (HAM-D, HAM-7) Montgomery Åsberg Depression Rating Scale (MADRS) Inventory for Depressive Symptomatology (IDS) Columbia Suicide Severity Rating Scale (C-SSRS) Dimensional Anhedonia Rating Scale (DARS)	Beck Depression Inventory-II (BDI-II)* Clinically Useful Depression Outcome Scale (CUDOS) Patient Health Questionnaire (PHQ-9) Patient Rated Outcome Measurement Information System (PROMIS) Quick Inventory for Depressive Symptomatology, Self-Rated (QIDS-SR) Suicidality Scale
Functioning	Multidimensional Scale of Independent Functioning (MSIF) Social and Occupational Functioning Assessment Scale (SOFAS) WHO Disability Assessment Scale (WHO-DAS)	Lam Employment Absence and Productivity Scale (LEAPS) Sheehan Disability Scale (SDS)* WHO-DAS, self-rated Work and Social Adjustment Scale (WSAS)*
Quality of life	Quality of Life Interview (QOLI)*	EuroQoL-5D (EQ-5D) Quality of Life, Enjoyment and Satisfaction Questionnaire (QLESQ)*
Side effects	UKU Side Effect Rating Scale Toronto Side Effects Scale	Frequency, Intensity and Burden of Side Effects Rating (FIBSER)

*Note*. These scales are examples for illustrative purposes only and are not officially endorsed by CANMAT. References for the scales are available in the Online Supplemental Materials.

* Copyright may require a fee for clinical use.

The use of scales for MBC is similar to the use of laboratory tests in that the results must be considered within the context of clinical assessment and care. There are many reasons why scale scores may not reflect a patient's clinical state. For example, the negative cognitive bias associated with MDD may interfere with accurate self-report, or some personality traits may contribute to chronic overreporting or underreporting on a symptom scale. Patients may also be concerned about over-reliance on a number and not their more complex issues.

While MBC is largely intended to increase clinical objectivity, it can also provide insight into patients’ subjective experiences. For example, when clinical evaluation varies substantially from patients’ symptom reporting, clinicians can use the discrepancies to explore individual factors that can colour patients’ perceptions about symptoms. These discussions are likely to inform about patients’ cognitive schemas and can enhance therapeutic rapport. Hence, scales should complement, not replace, the clinical interview and the clinician's comprehensive understanding of the patient.

MBC is also an integral component of collaborative care, where it serves as an essential means to communicate about a patient's clinical status and progress within the treatment team. MBC provides assessment continuity in settings where different providers manage the patient's care, e.g., when patients see different physicians at a clinic or where different providers manage medications and psychotherapy.

### Q.5.b. What are Operational Definitions for Improvement, Response, and Remission?

Incorporating scales into clinical management can help detect early improvement, clinically significant change, and symptom remission. Furthermore, MBC can identify cases when patients are not responding adequately to treatment. Studies show that clinicians and patients may not always recognize a lack of response or remission, highlighting the limitations of relying on general impressions.

Scales can be used to operationalize the definitions for improvement, response, and remission (discussed in Q.2). Early improvement is often defined with a symptom scale as a reduction in score of 20% or greater from baseline (within 2 to 4 weeks after treatment initiation), whereas response is defined as 50% or greater improvement from baseline. Symptom remission is indicated by a specific threshold score for each scale, e.g., a score of 7 or less on the Hamilton Depression Rating Scale (HAM-D), 10 or less on the Montgomery-Åsberg Depression Rating Scale (MADRS), and 4 or less on the PHQ-9. Some definitions of remission also specify a minimum duration, e.g., the DSM-5 defines remission as the absence or near absence of symptoms for at least 2 months.

MBC can be integrated into medication algorithms, with scale scores supporting clinical assessment and judgement for treatment decisions. For instance, lack of early improvement (i.e., less than 20% reduction in symptom scores at 2 to 4 weeks) after initiating an antidepressant is strongly associated with nonresponse and nonremission at later time points. Hence, a lack of early improvement by 4 weeks would trigger consideration of increasing the dose or switching the medication 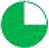.

While the total score on a scale is a useful proxy for overall severity, individual item scores may also be informative. For example, a total score of 4 on the PHQ-9 may indicate symptom remission, but a rating of 3 on the insomnia item suggests that residual sleep issues should be addressed. By examining both total and individual item scores, clinicians can comprehensively assess symptom profiles and tailor treatments accordingly.

The concept of recovery extends beyond symptom relief to include functional outcomes and quality of life. While there are few consensus definitions of response or remission in terms of overall functioning or quality of life, many validated scales measure changes in these important domains with defined mean scores for the general population (Table 5.2).

### Q.5.c. How is Measurement-Based Care Implemented?

Although recommended by quality agencies and clinical guidelines, many barriers still exist to limit the widespread adoption of MBC in clinical practice. Clinicians often feel that using MBC takes too much time and that discussing scales with patients takes time away from the clinical encounter. Clinicians also may perceive that scales do not capture important aspects of a clinical situation or that the act of completing scales may interfere with the therapeutic alliance. Other barriers to MBC implementation include insufficient training on scale usage and workflows, misbeliefs about the feasibility and efficacy of MBC, challenges with language and cultural barriers to using scales, and implementation and opportunity costs associated with changing workflows.

While acknowledging these concerns, many of these perceived barriers can be addressed. Practical tips for incorporating MBC in clinical settings include using simple PROMs that are free of charge, emailing scales to patients to complete prior to their appointment, and having patients complete scales in the waiting room before the appointment. Mobile and internet apps are also available that allow patients to complete validated scales and track their scores on their own time. For example, Apple has integrated the PHQ-9 and GAD-7 into its Health mobile app, so that scores can be tracked alongside other health indicators.

Scales for MBC can be helpful when administered every 2 to 4 weeks during the acute phase of treatment when active treatment decisions are being made. During the maintenance phase or chronic states of MDD, scales can be used less frequently so that patients do not become frustrated with repeated testing and scores that may not change. A key component of MBC involves longitudinally tracking scores from scales and questionnaires. The simplest approach is to document the total scores in the progress notes during each visit. Electronic medical records (EMRs) can be modified to track scores over time in the same way as laboratory results. Internet-based and mobile solutions for MBC are also available for clinical settings that do not have EMRs or where EMRs cannot be easily reconfigured.

### Q.5.d. What Laboratory Tests Should be Monitored During Treatment?

Routine laboratory tests are not necessary to monitor antidepressant treatment for MDD (Table 5.1), although some blood tests are indicated in specific clinical situations 7. For instance, liver function tests should be done at baseline and every 6 to 12 months in patients with pre-existing liver diseases, and serum electrolyte monitoring should be considered for older patients (age 60 years and older) as they are more susceptible to hyponatremia with antidepressants 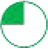. Moreover, when adjunctive agents (e.g., lithium, atypical antipsychotics, and ketamine) are used, lab tests tailored to the specific medication should be monitored. For example, patients taking lithium should have blood tests (lithium levels, electrolytes, calcium, creatinine, eGFR, and TSH) checked at baseline and every 6 to 12 months thereafter, or whenever the clinical status or lithium dose changes. For those on longer-term ketamine/esketamine treatment, periodic urinalysis is recommended to check for cystitis, a potential long-term side effect of ketamine use.

Weight gain is associated with MDD both as a symptom of illness and as a medication side effect. Therefore, regular monitoring of weight, glucose, and lipid profiles is recommended in patients taking medications associated with weight gain, according to obesity management guidelines 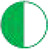.

## Question 6. What Should be Done When a Patient is Better?

### Q.6.a. How is Remission Maintained?

Maintaining remission is an important goal, as the risk of recurrence increases with each subsequent depressive episode. For psychological treatments, booster sessions may be helpful to retain and encourage strategies to maintain remission. Similarly, optimizing pharmacotherapy and treatment adherence will help individuals stay well. This can be done by maintaining the lowest effective dose used to achieve remission and minimize side effects, and regularly monitoring for emerging symptoms and side effects using MBC.

Attention to modifiable lifestyle factors, such as exercising regularly, avoiding substance misuse, adhering to a healthy diet, and having quality sleep, plays an important role in the prevention of depression (see Q.2.f). Finally, peer support, defined as giving and receiving help from individuals with lived experiences of mental illness, is a central element in recovery-oriented practices and can be delivered in various ways (e.g., individual, group, online, etc.). Alongside standard treatments, peer support interventions have a small but positive effect on personal recovery (living a satisfying and hopeful life) 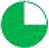.

### Q.6.b. How is Recurrence Prevented?

Once patients achieve symptom remission, maintenance pharmacotherapy and psychotherapy are both effective strategies for preventing a depressive recurrence (Table 6.1). Maintenance medication treatment can reduce relapse rates by 50% compared to placebo, and flexible dose adjustments are more effective than fixed doses. In recent, large-sample RCTs examining maintaining or discontinuing antidepressants in stably treated patients, medication maintenance was associated with lower rates of recurrence during 1-year and 3-year follow-up, whether or not patients had CBT during acute treatment 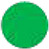.

**Table 6.1.**
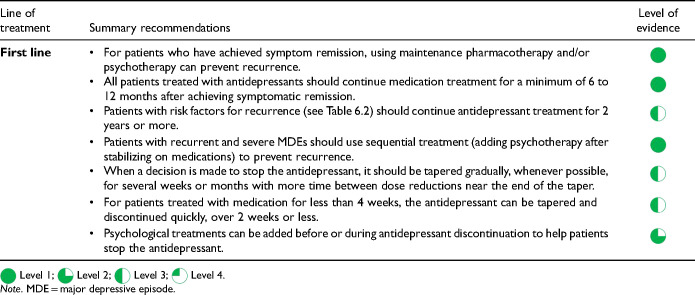
Summary Recommendations for Maintenance Antidepressant Treatment.

The 2016 CANMAT guidelines recommended that patients maintain treatment with antidepressants for 6 to 9 months after achieving symptomatic remission. New meta-regression evidence suggests that extending maintenance treatment for 6 to 12 months after remission adds benefit compared to stopping before 6 months 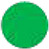. Hence, the recommendation has been adjusted to maintain antidepressants for 6 to 12 months after remission (Table 6.1).

Maintenance psychotherapy can also prevent relapse and recurrence. Several meta-analyses and a network meta-analysis show that various psychological treatments (CBT, MBCT, IPT, CBASP, and PST) are superior to control conditions at preventing depressive relapse, with comparable effects among the different types of psychotherapy 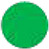. Evidence also supports the use of booster sessions at regular intervals (e.g., 4 sessions over 12 months) as an effective strategy for relapse prevention with MBCT. Psychotherapy appears to be as effective as antidepressants for preventing recurrence and may confer longer-lasting benefits by helping patients acquire more adaptive coping strategies over time. As a result, psychological interventions should be considered as first-choice options for mild to moderate severity MDD.

The sequential treatment model consists of adding or switching to psychotherapy in the maintenance phase, after a response to acute phase pharmacotherapy. Sequential treatment also has meta-analytic evidence for the prevention of recurrence, even when antidepressants are stopped. Adding psychotherapy (with CBT and its variations the best studied) also offers the benefits of treating residual symptoms and increasing psychological well-being and resilience. Pharmacotherapy and sequential treatment with psychotherapy are recommended especially for individuals with recurrent and severe MDEs, who carry a high risk of recurrence with monotherapy. For these cases, pharmacotherapy should be continued during psychotherapy treatment 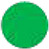 (Table 6.1).

### Q.6.c. Who Needs Longer-Term Antidepressant Treatment?

While 6 to 12 months of maintenance antidepressant treatment is recommended for all patients, some will require longer-term maintenance because they have risk factors for recurrence and chronicity (Table 6.2). Most risk factors are longitudinal and part of a continuum of exposure to depression and other psychological stressors throughout the lifespan. A systematic meta-review identified residual symptoms and a history of maltreatment or abuse during childhood as robust risk factors for recurrence. Other risk factors, including longer episode duration and greater severity of depressive episodes, have less evidence to support a higher risk of recurrence. Some evidence suggests that prior depressive episodes and residual symptoms could indicate a kindling effect, in which subsequent depressive episodes are triggered by milder stressors, and whereby certain individual characteristics such as early childhood adversity and neuroticism may contribute to a heightened sensitivity to life stressors over time.

**Table 6.2.** Risk Factors for Recurrence of Depressive Episodes.

Persistent residual symptoms* (e.g., anhedonia, sleep problems, and cognitive dysfunction) History of childhood maltreatment* Greater severity of depressive episodes Chronic depressive episodes Presence of medical comorbidities (psychiatric or nonpsychiatric) Greater number of previous episodes Poor social support Persistent stressful life events

* These have robust evidence as risk factors for recurrence.

For patients with risk factors for recurrence, antidepressant treatment should be continued for 2 years or more 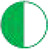 (Table 6.1). Support tools using machine learning algorithms have been developed to estimate the risk of recurrence based on an individual's risk profile, to assist clinicians and patients in making informed decisions about maintenance treatments. These risk-prediction models look promising, but further validation is required before they can be recommended for clinical use.

### Q.6.d. How Should Antidepressant Treatment be Discontinued?

Up to 50% of patients may experience discontinuation symptoms when stopping long-term use of antidepressants, especially with abrupt stopping. Discontinuation symptoms may include flu-like symptoms, insomnia, nausea, imbalance, sensory disturbances, and hyperarousal (the FINISH mnemonic). These emergent symptoms generally occur within a few days after a decrease in dose or stopping the antidepressant, are mild to moderate in severity, and resolve within a few weeks. However, later onset, greater severity, and longer duration of symptoms lasting several months or longer have been reported (see Box 6.1). Antidepressants with a shorter half-life, such as paroxetine and venlafaxine, are associated with a greater incidence/severity and a quicker onset of discontinuation symptoms. Table 6.3 shows the risk of discontinuation symptoms for various antidepressants.

Box 6.1. Protracted Discontinuation Symptoms and Hyperbolic Tapering Schedules.

Persistent discontinuation syndromes, with severe, potentially irreversible symptoms persisting beyond 6 weeks, have been described after stopping long-term antidepressant treatment. However, differing rates in the literature suggest that these discontinuation syndromes are heterogeneous, often occur with overlapping conditions, and require individual attention and assessment. In addition, since protracted discontinuation symptoms have mostly been reported in case reports, user surveys and internet forums, more rigorous studies are needed to establish their frequency, severity, and risk.

It has been suggested that, at the lower end of the therapeutic dose range, linear dose reduction may still result in a disproportionately large reduction in serotonin transporter inhibition, which may contribute to severe withdrawal symptoms. As a result, instead of decreasing the dose by a fixed amount (e.g., 10 mg), a “hyperbolic dose reduction” approach has been proposed, which targets a fixed percentage (e.g., 10%) reduction in serotonin receptor occupancy with each dose decrease. However, this approach is extrapolated from receptor studies using positron emission tomography (PET) in small numbers of participants and has not been evaluated in RCTs. The dosing schedule also presents a challenge in clinical practice, as it relies on compounding pharmacies to prepare liquid formulations or encapsulated nonstandard doses of antidepressants to achieve the required low doses. Hence, there is insufficient evidence to recommend hyperbolic tapering schedules.

**Table 6.3.** Risk of Antidepressant Discontinuation Symptoms.*

Risk of discontinuation symptoms	Antidepressant
High risk	Paroxetine Venlafaxine
Moderate risk	Citalopram Desvenlafaxine Duloxetine Escitalopram Fluvoxamine Levomilnacipran Milnacipran** Sertraline Vilazodone Tricyclic antidepressants Monoamine oxidase inhibitors
Low or minimal risk	Agomelatine** Bupropion Fluoxetine Mirtazapine Vortioxetine

*The risk categories are based on clinical studies, but the risk and severity of discontinuation symptoms may vary for individual patients and specific medications.

**Not available in Canada.

It is important to differentiate discontinuation symptoms from early symptoms of recurrence since misdiagnosing these emergent symptoms can prolong antidepressant use unnecessarily. Discontinuation symptoms generally have an earlier onset (within days rather than weeks), are often characterized by somatic symptoms (e.g., nausea, dizziness, and shock-like sensations) that differ from those of the initial depressive episode, and improve rapidly if the dose is restored to its previous level. Although gradual tapering of antidepressants may not prevent discontinuation symptoms, they typically occur less frequently compared to abrupt stopping and are less severe. Psychological support may also help with antidepressant discontinuation. Adding psychotherapy may allow patients to stop medication without increasing the risk of relapse and may help mitigate discontinuation symptoms while tapering the antidepressant.

However, there is no consensus on the best strategy for stopping antidepressants. Few relapse prevention studies have systematically differentiated discontinuation symptoms from symptoms of recurrence, and fewer have investigated strategies for stopping antidepressants. A 2021 Cochrane review examined the effectiveness of different approaches to discontinuing long-term antidepressants, including abrupt discontinuation, and tapering the antidepressant with or without psychological support. There was no clear evidence to support abrupt or tapering schedules. There is low-quality evidence that providing psychological support (primarily CBT or MBCT) is beneficial while tapering. All these results had low certainty because of the methodological limitations of the included studies. A hyperbolic tapering schedule has been suggested but there is insufficient evidence for its efficacy (see Box 6.1).

Given the uncertainty about discontinuation strategies, CANMAT recommends a pragmatic approach. Unless there are clinical reasons requiring rapid discontinuation (e.g., serious side effects, serotonin syndrome, etc.), antidepressants should be tapered gradually over several weeks or months (except for fluoxetine, which does not need tapering because of its long half-life), extending the time between dose reductions towards the end of the taper 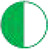. In situations where antidepressants have been used for less than 4 weeks (e.g., a decision to stop the medication because of side effects), a fast-tapering schedule over 2 weeks or less can be used 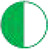. Psychological treatments may also be used during or preceding antidepressant discontinuation, if available and acceptable to the patient, to reduce or mitigate discontinuation effects 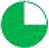. If discontinuation symptoms are severe when the dose is decreased, patients can return to the previous higher dose, with a slower tapering schedule used subsequently. Alternatively, SSRI and SNRI antidepressants can be switched to a long-acting medication such as fluoxetine, which can then be tapered. During antidepressant tapering, patients should be monitored using a validated measure such as the Discontinuation-Emergent Signs and Symptoms (DESS) Scale 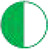.

## Question 7. What Should be Done When a Patient is not Better?

### Q.7.a. What Contributes to a Poor Response to Treatment?

In real-world populations, approximately half of patients with MDD achieve response (>50% reduction in symptom severity) after 8 weeks of antidepressant monotherapy, while about a third attain full symptom remission; only a quarter of nonremitters who receive a second pharmacological treatment will reach remission. For first-treatment patients, the remission rates are higher, but almost half will not achieve remission with antidepressants after 6 months of treatment. Hence, inadequate response to treatment is a common clinical challenge.

Several clinical, comorbidity, and medication factors may contribute to insufficient treatment response (Table 7.1). Comorbid psychiatric conditions associated with poor treatment response include anxiety disorders, ADHD, post-traumatic stress disorder, substance use disorders, and personality disorders. Comorbid nonpsychiatric medical conditions may also be associated with poor response to antidepressants. For example, undiagnosed sleep apnea may be an important contributor to persistent residual symptoms of insomnia, fatigue, and low motivation. Clinicians should also be aware that comorbid conditions do not remain static over time. Hence, regular reassessment is important for patients with persistent symptoms.

**Table 7.1.** Factors Contributing to Poor Response to Initial Treatment.

Clinical factors	Treatment factors
Incorrect diagnosis (e.g., bipolar disorder) Demographic and illness characteristics (e.g., older age, female sex, younger age of onset, higher severity, increased number/duration of episodes, and trauma history) Psychiatric medical comorbidities (e.g., anxiety disorders, personality disorders, attention-deficit hyperactivity disorder, substance use disorders, etc.) Nonpsychiatric medical comorbidities (e.g., anaemia, obesity, sleep apnea, thyroid disease, etc.) Acute or chronic stressors	Inadequate dose of treatment Inadequate duration of treatment Side effects masking as symptoms Poor adherence to treatment Pharmacogenetic variability (e.g., rapid or slow metabolism of drugs)

### Q.7.b. What is the Difference Between Treatment-Resistant Depression and Difficult-to-Treat Depression?

“Treatment-resistant depression (TRD)” is a term used to describe a lack of response to initial treatments. There is no universal consensus on a definition for TRD, but the one most frequently used is failure to respond to 2 or more antidepressant trials at a therapeutic dose and adequate duration. This definition has been criticized because it neglects psychological and neurostimulation treatments, it assumes that switching antidepressants is the preferred initial strategy and neglects add-on strategies. Furthermore, “failure” is often not defined or defined differently from study to study, and it does not account for partial response or residual symptoms. Additionally, the term “resistant” has a negative connotation which suggests futility and may discourage patients and clinicians from further therapeutic interventions.

DTD is a term proposed to extend the TRD concept and to better characterize the collaborative journey of patients and their clinicians when standard treatments have not been effective. In the DTD model, the therapeutic focus shifts away from symptom remission towards symptom management, to achieve the best possible improvement in patient-prioritized outcomes such as functioning and quality of life. In the academic literature, DTD is used to describe persistent depression that has failed numerous standard treatments (in contrast to the minimum of 2 antidepressants defined by TRD) and is further along the treatment trajectory. In addition, compared to the term TRD, patients find DTD to be a more supportive, hopeful, and collaborative term. Hence, in these guidelines, we will use DTD as a holistic framework when discussing unsatisfactory responses to standard treatments and typically defined TRD when discussing medication options or studies that use the term.

### Q.7.c. How are Strategies Sequenced When There is a Poor Response to an Initial Antidepressant Treatment? 

The first steps in the assessment of poor response involve re-evaluating the diagnosis (e.g., a missed diagnosis of bipolar disorder), comorbidities, and adherence to treatment. In addition to clinical reassessment, laboratory investigations should be considered to rule out potential medical factors that can contribute to persistent symptoms and that require additional interventions. Pharmacogenetic testing may be helpful to identify pharmacokinetic reasons for poor response or unusual severity of adverse effects. Systematic, sequential, and MBC may enhance outcomes for MDD when initial treatments are not fully effective 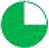.

**Figure 7.1.**
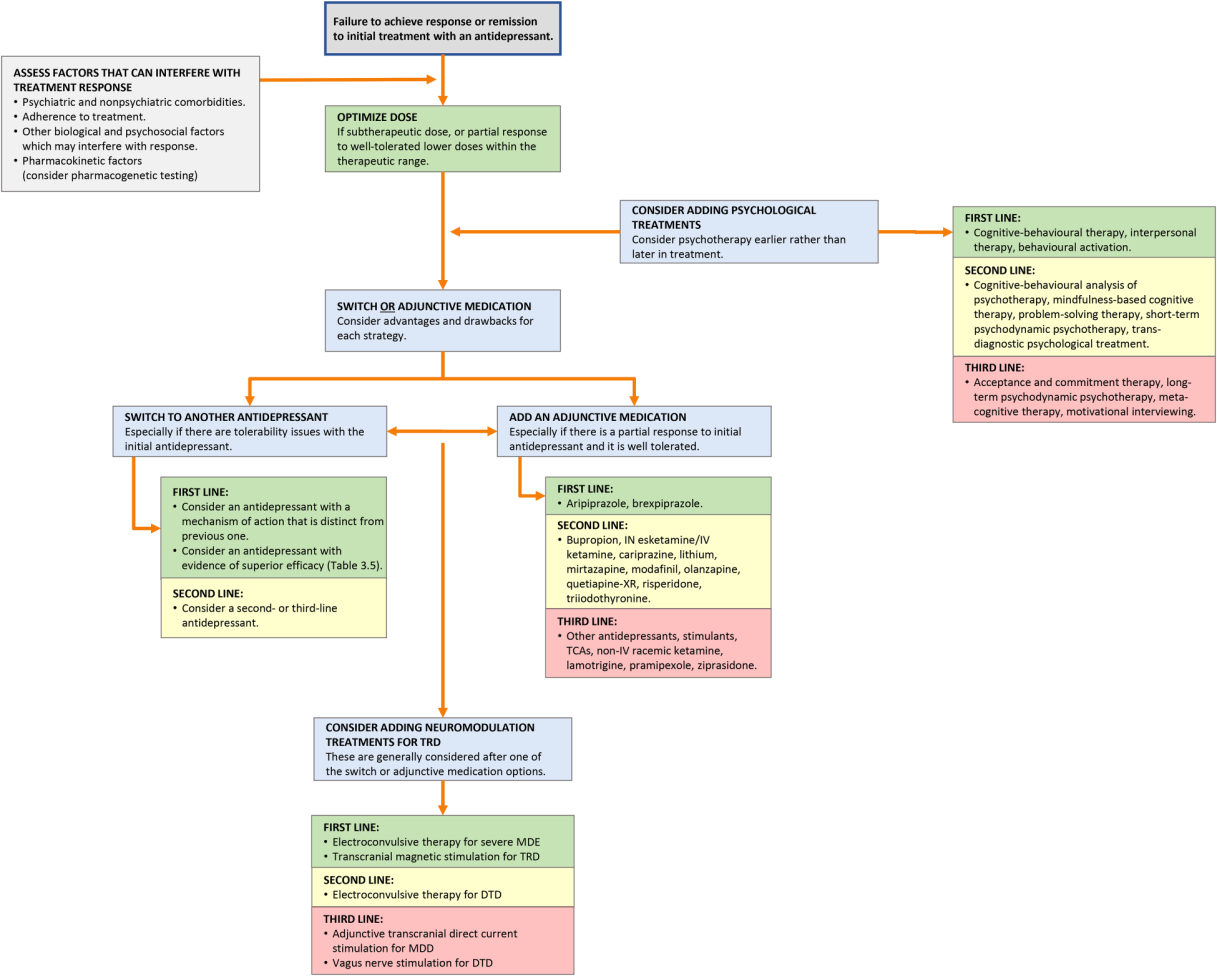
Algorithm for sequential treatment after suboptimal response to initial antidepressant medication.

*Note.* This algorithm refers primarily to medication treatments. Psychological treatments (see Table 3.2) should also be considered when patients have suboptimal responses to an initial medication. Neuromodulation treatments (see Table 8.3) are generally considered after a switch or adjunctive medication strategy has failed (i.e., for treatment-resistant depression). DTD = difficult-to-treat depression; MDD = major depressive disorder; TCA = tricyclic antidepressant; IN = intranasal; IV = intravenous; TRD = treatment-resistant depression; XR = extended release.

Strategies for poor response to an initial antidepressant include optimizing the dose, switching to another antidepressant, adding an adjunctive medication, and incorporating psychological and/or neuromodulation treatments. Given the limited evidence, CANMAT recommends that the sequencing of treatments should be based on a collaborative approach that integrates prior treatment history, the strength of the evidence for efficacy, potential for adverse events, and patient preference 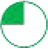 (Figure 7.1). Based on efficacy and low potential for side effects, first-line psychological treatments should be considered early in the treatment course 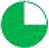. Neuromodulation treatments, while having good evidence for efficacy in TRD and DTD, are less likely to be considered early in the treatment algorithm because of limited accessibility and patient burden (see Q.8.d).

Optimizing the antidepressant dose is an important first step. Higher than minimal therapeutic doses are more effective (to a plateau around the equivalent of 50 mg of fluoxetine) but less well-tolerated 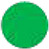. Hence, increasing the dose for nonresponse must be balanced against increasing side effect burden and poorer adherence.

After optimizing the dose, the main medication options involve switching to another antidepressant or adding an adjunctive medication. The evidence for the efficacy of switching antidepressants is inconsistent. Meta-analyses show that switching to another antidepressant is not superior to continuing the same one 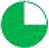 and there appears to be diminishing response rates for antidepressant monotherapy beyond the first switch.

Adjunctive strategies, especially with atypical antipsychotic agents (serotonin and dopamine activity modulators), have greater evidence for efficacy and shorter time to response or remission, but they generally have a greater side effect burden than antidepressant monotherapy 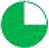. Moreover, RCTs directly comparing switching to adjunctive strategies have shown that adjunctive strategies have superior outcomes and often similar tolerability. Hence, while balancing individual risks, benefits, and patient preference, CANMAT recommends that adjunctive strategies be considered earlier in the treatment algorithm, after limited response to the first or second antidepressant trial 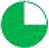.

For these medication strategies, a comprehensive review of previous medication trials, major side effects experienced, and whether there is a partial response, can inform the decision. Switching should be considered first when there is no response to the initial antidepressant or when the first medication has troublesome side effects. Adjunctive strategies should be considered first when there is a partial response to the initial antidepressant and there are minimal or no tolerability issues with the first medication.

### Q.7.d. When and how Should Antidepressants be Switched? 

Early improvement, often defined as a 20% or greater reduction in scores on a symptom rating scale within the first 4 weeks after starting an antidepressant, predicts a later response. Notably, if early improvement is not seen by 4 weeks, there is only a low likelihood of response or remission at 8 to 12 weeks. At that point, the decision should be to either increase the dose or to switch the antidepressant if there are tolerability concerns.

When switching, selecting a second antidepressant should consider factors such as side effect profiles, mechanism of action, and comparative efficacy (see Q.3.d). Several studies have shown that there is no difference in outcome between switching within a medication class and switching to a different class. Hence, when switching to another antidepressant, CANMAT recommends first selecting a first-line antidepressant with evidence for superior efficacy and a favourable tolerability profile 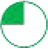 (Table 3.5). Of note is that TCA and MAOI agents may be useful in the treatment algorithm if there has been a poor response to first and/or second-line antidepressants (Box 7.1).

Box 7.1. Tricyclic Antidepressants and Monoamine Oxidase Inhibitors.

Tricyclic antidepressants (TCAs) and irreversible monoamine oxidase inhibitors (MAOIs) are efficacious treatments for MDD, but they are downgraded to second-line and third-line medications (Table 3.3), respectively, because of less favourable side effect and safety profiles compared to first-line agents, and/or the need for dietary and drug restrictions (Q.3.j). Some TCAs have a more favourable side effect profile than others. For example, nortriptyline has the fewest anticholinergic and hypotensive adverse effects; it has a good safety and efficacy profile in elderly and post-stroke depression populations.

These older medications continue to have an important role in managing treatment-resistant depression (TRD) and difficult-to-treat depression (DTD). TCAs have evidence for superior efficacy in TRD and may have specificity for subpopulations of patients with MDD, e.g., amitriptyline for comorbid painful conditions and clomipramine for comorbid obsessive-compulsive disorder. Another advantage of TCAs is that, in contrast to newer antidepressants, serum levels can be informative to adjust dosing. MAOIs are also efficacious in TRD and have a different mechanism of action compared to first-line and second-line agents. Hence, TCAs and MAOIs should be considered as therapeutic options when first-line and second-line medication treatments are not effective, with patients monitored carefully for side effects and potential for drug interactions.

The procedure for switching medications should be influenced by the likelihood of discontinuation effects, urgency of switching, and potential drug interactions. For most situations, given the low risk of drug interactions and side effects, the crossover “X” switch method can be used, i.e., slowly tapering the first medication while slowly titrating up the second 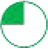. If there is less urgency for a switch, or a history of problems with discontinuation symptoms (see Q.6), or to avoid conflating discontinuation symptoms from new side effects, the washout “V” method can be used, i.e., tapering and discontinuing the first medication before starting the second 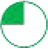. Switching to MAOIs requires at least 2 weeks’ washout from other serotonergic medications (5 weeks if switching from fluoxetine). Online tools are also available for advice on titration and switching schedules (e.g., SwitchRx.com).

### Q.7.e. What are the Benefits and Drawbacks of Adding an Adjunctive Medication? 

Adding an adjunctive agent retains partial treatment gains from the initial antidepressant, avoids discontinuation symptoms from stopping the first medication, and potentially adds complementary mechanisms of action that have a faster onset of response. These benefits are especially helpful when there has been a partial response to the first antidepressant, and it is well tolerated. Adding another medication may also target specific residual symptoms or side effects from the initial antidepressant.

The drawbacks of adjunctive treatments include the possibility of additive side effects, increased cost of treatment, and potential for drug–drug interactions 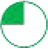. Furthermore, using multiple medications may contribute to decreased adherence to treatment 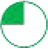. It should be noted, however, that adding an adjunctive agent at a low dose may accrue fewer side effects than increasing a single medication to higher doses 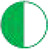. Finally, there is little evidence available for maintenance treatment with adjunctive agents, so it is unclear how long adjunctive medications should be continued.

### Q.7.f. How is an Adjunctive Medication Selected?

Selecting an adjunctive medication involves consideration of efficacy and tolerability, for both the adjunctive agent (Table 7.2) and the continued antidepressant. The various adjunctive agents have very different side effect profiles and potential drug–drug interactions should also be considered. If pharmacogenetic test results are available, these may help in treatment selection, especially in DTD 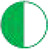. An often-used approach is to select an adjunctive medication which can address specific residual symptoms and/or side effects experienced by the patient (e.g., using a sedating medication when insomnia is present); there is, however, little evidence available to support this strategy. An important tip when considering adjunctive treatments is to minimize polypharmacy as much as possible by reassessing concurrent medications and discontinuing those with unclear benefits.

**Table 7.2.**
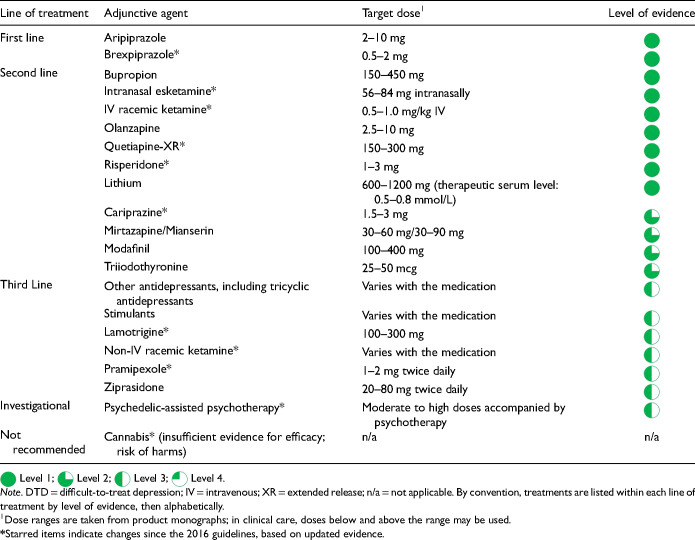
Summary Recommendations for Adjunctive Medications for (DTD).

### Q.7.g. What are the Risks and Benefits of Specific Adjunctive Medications?

#### Serotonin-Dopamine Activity Modulators (Low-Dose Atypical Antipsychotics)

Adjunctive treatment with serotonin-dopamine activity modulators (also known as atypical antipsychotic agents) has the most consistent evidence for efficacy in DTD (Table 7.2). Typically, lower doses of these medications are used for adjunctive treatment in MDD compared to other conditions such as bipolar disorder or schizophrenia. Most of the listed medications have evidence for efficacy, with network meta-analyses showing that they are more efficacious than placebo 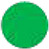. Aripiprazole and brexpiprazole are listed as first-line agents because of their efficacy and tolerability profile. Cariprazine is recommended as the second-line because there are fewer studies compared to the other agents. Ziprasidone is included as a third-line recommendation because of mixed efficacy results in RCTs and network meta-analyses.

Serotonin-dopamine modulating agents have very different receptor and side effect profiles. Common side effects with aripiprazole and brexpiprazole include akathisia and weight gain. Some agents with Level 1 evidence for efficacy are recommended as second-line treatments because of the increased risk of certain side effects, including olanzapine (weight gain and metabolic side effects), quetiapine-XR (sedation and metabolic side effects), and risperidone (hyperprolactinemia, sexual side effects, and extrapyramidal symptoms).

There are also potential long-term risks with these medications, such as tardive dyskinesia, although the risk is lower compared to older antipsychotics and is mitigated by the lower dosing used for MDD. In studies of conditions other than MDD, long-term use of these agents in older age may be associated with increased mortality. Inconsistent reports also suggest a slightly increased risk of breast cancer, although the latter may also be mitigated by lower dosing and using prolactin-sparing agents.

#### Adjunctive Use of a Second Antidepressant

Compared to serotonin-dopamine activity-modulating agents, antidepressants generally have a more favourable side effect profile when used as adjunctive treatment, but the evidence for efficacy is less robust. Meta-analyses find that adding a second antidepressant is associated with better treatment outcomes compared to switching, but there is significant heterogeneity across reports which limits confidence in the results 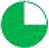. Adding mirtazapine/mianserin (antagonists of presynaptic α2-adrenoceptors) is superior to other combinations. Large RCTs have shown evidence for the effectiveness of adjunctive bupropion but the meta-analyses are inconclusive. Hence, these are recommended as second-line treatments for DTD. Adjunctive use of other antidepressants, including TCAs, continues to be recommended as a third line due to limited evidence and safety/tolerability issues among older agents.

#### Glutamate Modulators

Newer adjunctive agents target the glutamate system, which is implicated in the pathophysiology of MDD and has the potential for rapid onset of antidepressant effects. A CANMAT Task Force Report summarized the extensive evidence for rapid antidepressant effects of a single dose of intravenous (IV) racemic ketamine, a glutamate modulator acting as an NMDA receptor antagonist 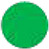, with recent studies also showing effectiveness and safety of repeated infusions 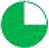. Similarly, several meta-analyses showed efficacy for intranasal esketamine 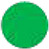, which is approved by several regulatory agencies as an add-on treatment to an SSRI/SNRI or other antidepressant, for patients failing at least 2 adequate antidepressant trials.

Intravenous racemic ketamine and intranasal esketamine both have shown mood-independent rapid reductions in suicidal ideation, with the anti-suicidal effects of IV ketamine extending as long as 1 week after a single infusion 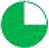. There is also growing evidence for relapse-prevention efficacy for both medications, with maintenance doses administered every 1 to 4 weeks 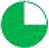. However, side effect potential and feasibility issues (i.e., the required monitoring of blood pressure and dissociative side effects) have downgraded adjunctive ketamine and intranasal esketamine to second-line recommendation.

Several meta-analyses also suggest evidence for efficacy of alternate routes of administration of racemic ketamine, namely, oral, nasal, and intramuscular. However, the reported treatment protocols (dosage, frequency, formulation, etc.) are highly variable and results are mixed. Given these issues, alternate routes of administration of racemic ketamine are downgraded to third-line recommendation 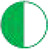.

Lamotrigine is another glutamate modulator more frequently used for bipolar depression. A meta-analysis in unipolar TRD found higher response rates with adjunctive lamotrigine compared with antidepressant monotherapy, although the trials were of low-quality 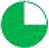. Adjunctive lamotrigine was well tolerated in these studies, but prescribers must be aware of the risk of Stevens–Johnson Syndrome, a severe and potentially fatal allergic skin reaction, and the need to initiate treatment at low doses and titrate up gradually. Given these issues, lamotrigine is recommended as a third-line adjunctive agent.

#### Stimulants

Methylphenidate and other stimulants have been studied for the treatment of MDD with mixed results. Outcomes differ widely across stimulants when used as monotherapy or adjunctive treatment and a network meta-analysis found mixed efficacy results. Side effects of stimulants include anxiety, irritability, jitteriness and tremors, headaches, insomnia, and appetite/weight loss. Additionally, tolerance may develop so that early improvements (especially in domains of energy, motivation, and cognition) may quickly wane over time. Consequently, CANMAT recommends stimulants as third-line adjunctive treatments 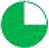.

Compared to amphetamine-based agents, adjunctive treatment with modafinil has more consistent evidence for efficacy, albeit with small effect sizes 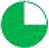. One systematic review evaluated modafinil for potential pro-cognitive effects in MDD and noted improvement in executive functioning 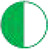. Taken together, CANMAT maintains the second-line recommendation for adjunctive modafinil.

#### Other Medications

Lithium and triiodothyronine were listed as second-line adjunctive agents in the CANMAT 2016 guidelines. Both medications were included in a network meta-analysis evaluating all adjunctive options. While both lithium and triiodothyronine had evidence for efficacy, the included RCTs predated 2003, had small sample sizes and involved adjunctive use with TCAs only. Lithium also requires serum level monitoring and triiodothyronine requires monitoring of thyroid levels. Hence, lithium and triiodothyronine continue to be second-line recommendations.

Adjunctive use of pramipexole, a dopamine agonist, was shown to have both acute and longer-term antidepressant effects in a meta-analysis of MDD and bipolar depression. In small-sample RCTs, patients treated with pramipexole had a superior response rate compared with placebo and a similar response to SSRIs 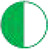. Acceptability and tolerability were good, with nausea being the most frequent side effect. As such, pramipexole has been added as a third-line adjunctive recommendation.

There are no RCTs of cannabis and related compounds such as cannabinoids for MDD, but there is evidence that cannabis use worsens depression outcomes. Hence, cannabis is not recommended as a treatment for MDD 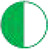.

### Q.7.h. What Psychological Treatments are Effective After Poor Response to the Initial Antidepressant? 

There are few studies of psychotherapy for patients showing poor response to antidepressants. A Cochrane review of patients with poor response to an initial antidepressant found moderate-quality evidence supporting psychotherapy (primarily from 1 large trial of CBT) added to usual care (including antidepressants) for reducing depressive symptoms and increasing response and remission rates 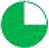. Hence, CBT is recommended as a second-line adjunctive treatment, with medications, for DTD.

Psychedelic-assisted psychotherapy was recently reviewed in a CANMAT Task Force Report. Several RCTs show efficacy for single-dose psilocybin-assisted psychotherapy in patients with TRD 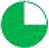. Although these results are promising, they are limited by ongoing methodological issues (e.g., lack of blinding, inadequate assessment of expectancy bias, heterogeneity of psychological support, and lack of long-term safety data). Hence, psilocybin- and other psychedelic-assisted psychotherapies are considered investigational treatments (Table 7.2).

## Question 8. When Should Neuromodulation Treatments be Used?

### Q.8.a. What are Neuromodulation Treatments?

Neuromodulation (also referred to as neurostimulation) refers to treatments that alter central nervous system activity through the application of electrical or magnetic stimulation of the brain. These treatments are generally used when initial therapies have not been successful. Some neuromodulation treatments are noninvasive (e.g., ECT, magnetic seizure therapy [MST], repetitive transcranial magnetic stimulation [rTMS], and transcranial direct current stimulation [tDCS], while others involve surgical placement of electrodes (e.g., vagus nerve stimulation [VNS] and deep brain stimulation [DBS]).

### Q.8.b. What Noninvasive Neuromodulation Treatments are Available?

#### Electroconvulsive Therapy

ECT involves the delivery of an electrical stimulus via electrodes placed on the scalp, resulting in the induction of a brief generalized seizure. ECT is delivered under general anaesthesia and after a muscle relaxant has been administered to minimize the physical manifestations of the seizure and its potential complications. ECT remains one of the most effective treatment options for patients with TRD. However, the clinical use of ECT is often hindered by stigma, the need for general anaesthesia, concern about cognitive adverse effects, and high rates of relapse after acute treatment.

ECT has demonstrated evidence of efficacy and tolerability in the treatment of depressive episodes, with response rates ranging from 65% to 75% 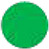. ECT may be especially effective in older patients, those with psychotic or catatonic features, and more severely depressed patients. Importantly, results of retrospective cohort analyses conclude that the benefits of ECT outweigh the risks among hospitalized MDD patients, with no evidence of increased risk of serious medical events. Furthermore, ECT significantly reduced the risk of suicide in the year after discharge from hospital.

An acute course of ECT usually requires 6 to 12 sessions, with no difference in response outcomes between sessions given twice or thrice weekly 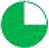. Refinements in the delivery of ECT have focused on electrode placement and stimulus energy, based on the waveform of electrical stimulus and seizure threshold (the minimum amount of current required to produce a measurable seizure) (Table 8.1). Ultrabrief pulse (∼0.3 ms) waveforms with right unilateral electrode placement are slightly less effective than brief pulse (∼1.0 ms) but appear to have fewer cognitive adverse effects (see below). In contrast, bitemporal brief pulse ECT may have greater efficacy than other protocols but carries a higher cognitive burden. Furthermore, large-scale studies have demonstrated superior quality of life outcomes for right unilateral positioning compared to either bitemporal or bifrontal positioning. Hence, both efficacy and tolerability should be considered when choosing an ECT protocol for a particular patient.

**Table 8.1.**
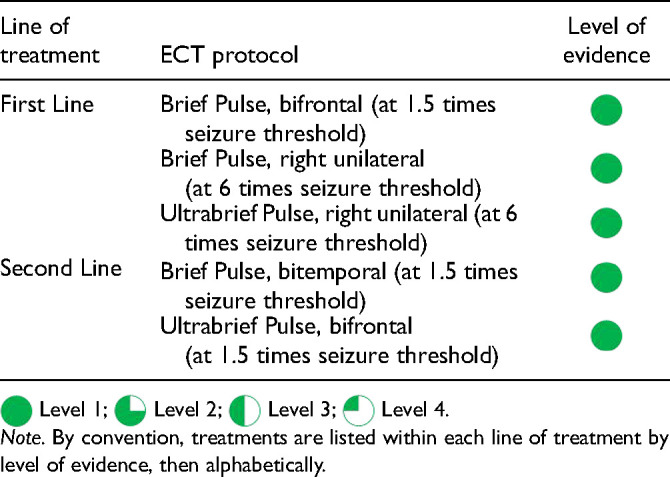
Recommendations for Electroconvulsive Therapy (ECT) Protocols.

Antidepressants and other medications can usually be continued during ECT treatment 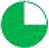. A meta-analysis found that concurrent use of antidepressants during a course of ECT improved outcomes, but this was low-quality evidence based on older trials. Ketamine has not been shown to improve outcomes with ECT, whether as an anaesthetic agent or with single-dose IV infusions given during a course of ECT. Some concomitant medications may interfere with ECT efficacy (e.g., benzodiazepines and anticonvulsant medications) or worsen cognitive side effects (lithium and cannabis), and should be discontinued or held before ECT.

ECT is generally safe and well tolerated. Rates of cardiac events and mortality associated with ECT are very low and similar to the risks for general anaesthesia. Adverse effects on cognition are of most concern, although modern ECT techniques, including unilateral electrode placement and refined electrical dosing, have reduced both the incidence and severity of cognitive side effects. Cognitive effects experienced immediately following an ECT session include transient disorientation, confusion, and memory lapses, particularly anterograde amnesia for events surrounding the treatment period. These effects typically resolve within days to weeks. While there is potential for long-term cognitive effects, particularly retrograde amnesia for events remote to ECT, recent meta-analyses of neuropsychological assessments demonstrate that cognitive performance is unchanged or improved at least 1 month after a course of ECT. Nevertheless, some patients report significant and distressing gaps in autobiographical memory long after ECT, and the discrepancy between cognitive performance and subjective memory dysfunction remains controversial. Of note is that subjective cognitive dysfunction is commonly experienced during an acute episode of MDD and is associated with persisting depressive symptoms.

Although ECT has high response rates during acute treatment, recurrence occurs in 60% to 80% of patients at 6-month follow-up. Given the high recurrence rate, a maintenance strategy should be implemented following ECT, with either pharmacotherapy or continuation ECT. Unfortunately, there is only limited data to guide this treatment choice. Continuation ECT (administered at increasing intervals from once a week to once a month) shows better efficacy in preventing recurrence after ECT, compared to maintenance pharmacotherapy 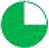. However, few medications have been systematically studied for maintenance pharmacotherapy. Lithium, when combined with either nortriptyline or venlafaxine-XR, is shown to be superior for relapse prevention compared to either antidepressant alone 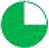. There are no differences in cognitive outcomes between maintenance ECT and pharmacotherapy. Given this limited evidence, the choice for maintenance treatment following successful ECT must consider factors such as previous ECT responses, episode severity and degree of treatment resistance, consequences of recurrence, adverse effects experienced during ECT, and patient preference.

#### Transcranial Magnetic Stimulation

rTMS involves stimulation of cortical neurons using powerful, focused magnetic field pulses that are externally applied over the scalp using a magnetic coil. Several coil shapes have been designed to improve targeting. Unlike ECT, rTMS does not require anaesthesia and has no cognitive side effects. Depending on the protocol, conventional rTMS is delivered for 20 to 40 min each session, with daily sessions 5 days a week for 4 to 6 weeks. Parameters for rTMS include the type of coil, location of the scalp where the transcranial magnetic stimulation (TMS) coil is placed (to direct the magnetic pulses to a specific cortical brain region), frequency of stimulation, and number and duration of magnetic pulses (trains).

Repetitive TMS shows efficacy for DTD, with response rates of 40% to 50% 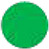 (Table 8.2), although the strength of evidence is dependent on treatment targets and protocols. High-frequency (5 Hz or higher) rTMS delivered over the left dorsolateral prefrontal cortex (DLPFC) has the largest evidence base and is recommended as a first-line protocol, along with low-frequency (1 Hz or lower) rTMS delivered over the right DLPFC. Bilateral rTMS, targeting each DLPFC sequentially, is recommended as a second-line protocol but may be considered first-line for late-life depression. Clinical features associated with lower remission rates after rTMS include higher baseline severity of depressive and anxiety symptoms and a greater number of previous antidepressant trial failures.

**Table 8.2.**
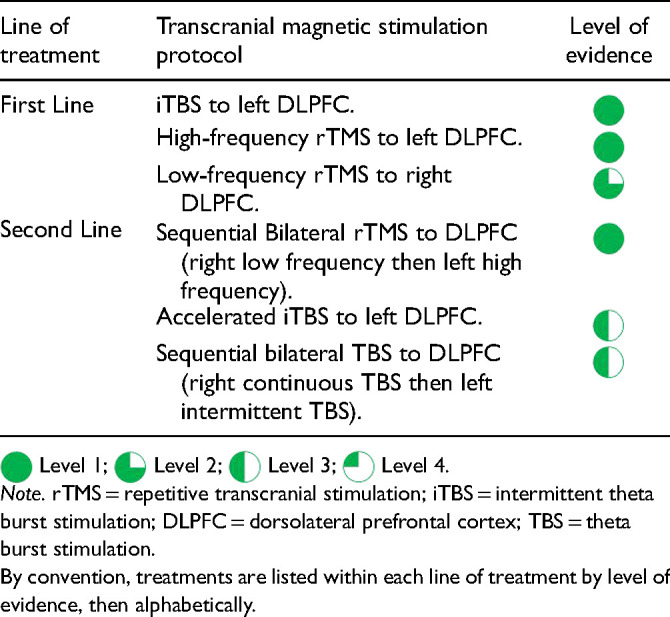
Summary Recommendations for Repetitive Transcranial Stimulation (rTMS) Protocols.

Unlike conventional rTMS, which delivers single pulses or trains of stimulation at a frequency of up to 20 Hz, theta burst stimulation (TBS) delivers bursts (trains) of 3 pulses at a very high frequency (50 Hz). Intermittent TBS (iTBS) consists of 2 s trains of TBS repeated every 10 s for a total of 190 s (just over 3 min), which is thought to increase cortical excitability, while continuous TBS (cTBS) consists of a continuous 40 s train, which is believed to decrease cortical excitability. TBS is recommended as a first-line protocol 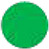 (Table 8.2). A noninferiority study found that iTBS was as effective and tolerated as high-frequency rTMS but with the advantage of significantly shorter daily treatment sessions. Newer TBS protocols use accelerated delivery, with and without individualized functional MRI targeting, with multiple treatment sessions given each day to reduce the overall duration of a treatment course to as short as 5 days. Accelerated iTBS to the left DLPFC has evidence for efficacy 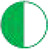 and a large RCT found no significant differences between accelerated bilateral TBS and high frequency rTMS.

In most trials, rTMS is delivered concurrently with pharmacotherapy, while requesting participants not to change their medications or dosing. Specific medications, such as benzodiazepines, may negatively affect response rates according to retrospective reports, while concurrent antidepressant use may augment response rates.

#### Transcranial Direct Current Stimulation

tDCS modulates cortical excitability through the delivery of a constant, weak electrical current via surface scalp electrodes. The advantages of tDCS include inexpensive equipment and relative ease of use such that tDCS can be delivered at home with few side effects or safety concerns. Meta-analyses have shown that active tDCS is more effective than sham in the treatment of mild to moderate depression, especially when combined with an antidepressant 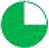. However, the quality of evidence is significantly limited by heterogeneity in stimulation parameters and patient populations, high sham responses, and negative results from recent large RCTs. Current evidence also does not support the use of tDCS for those with severe depression or DTD 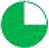.

#### Magnetic Seizure Therapy

MST was developed in part to mitigate the cognitive side effects of ECT. Instead of direct electrical current, MST uses vertex or frontal placement of single or paired circular coils to deliver TMS. The high intensity and frequency of TMS induces an electrical field that is strong enough to elicit a generalized seizure. MST has shown promising results in small-sample RCTs, with similar efficacy to right unilateral, ultrabrief ECT, but fewer adverse effects. However, without more definitive studies, MST is still considered an investigational treatment 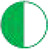.

### Q.8.c. What Surgical Neuromodulation Treatments are Available?

Surgical neuromodulation treatments offer the capability of “always-on” electrical stimulation to change the function of neural targets specifically and selectively (e.g., nodes in the neural circuit) that are inaccessible by noninvasive methods. Stimulus parameters can also be individually and continuously adjusted to either modulate the node (i.e., change the node function) or to ablate it (i.e., remove the node from the circuit).

#### Vagus Nerve Stimulation

VNS involves surgically implanting an electrode around the left vagus nerve in the mid-cervical region of the neck, connected to a pulse generator implanted subcutaneously in the chest wall. Low-level intermittent electrical stimulation of the left vagus nerve is believed to stimulate the nucleus tractus solitarius and its cortical and subcortical connections to achieve antidepressant effects. The VNS procedure was first approved by regulatory agencies for the treatment of epilepsy, and subsequently for TRD in Canada (2001) and the United States (2005).

To date, there are very few RCTs involving VNS, and these studies have failed to demonstrate the efficacy of VNS compared to sham control conditions in the short term. However, systematic reviews of open-label studies in patients with DTD suggest that longer-term treatment (2 to 5 years) with VNS results in superior response and remission rates compared to treatment-as-usual cohorts 6. VNS is generally safe and well tolerated, with the most common side effects involving pain related to device implantation, voice hoarseness or alteration (due to intermittent stimulation of the larynx), coughing, headache, sore throat, and neck pain.

#### Deep Brain Stimulation

DBS involves neurosurgical implantation of electrodes in specific brain regions, connected to a subclavicular-implanted pulse generator delivering constant or intermittent electrical stimulation. The most frequently studied target for DBS in DTD is the subcallosal cingulate cortex, while other studied targets include the ventral capsule/striatum, the nucleus accumbens, the anterior limb of the internal capsule, and the medial forebrain bundle.

Given that DBS involves a neurosurgical procedure, most studies in MDD have small sample sizes and involve patients with highly refractory illnesses, including ECT nonresponders. Open-label clinical studies of DBS demonstrate high response rates in participants with DTD across several targets. However, a sham-controlled RCT of DBS targeting the subcallosal cingulate cortex did not show efficacy after 6 months of blinded treatment. Longer-term observational studies suggest that, like VNS, the therapeutic effects of DBS increase over time 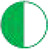. In addition to transient side effects associated with stimulation, several studies have reported serious adverse events related to neurosurgery implantation.

### Q.8.d. When Should Neuromodulation Treatments be Selected?

Generally, noninvasive neuromodulation treatments are safe and well tolerated. Notably, ECT is more efficacious for DTD compared to other noninvasive neuromodulation treatments, although it carries a greater side effect burden. However, rTMS is typically recommended for patients with TRD (and ECT for DTD) after the failure of first-line psychotherapy and medication treatments (Table 8.3) because of feasibility issues, including limited availability (especially in the public health sector), patient burden (e.g., requiring daily clinic visits for rTMS), and the need for specialized personnel (e.g., anaesthesiologists for ECT). Although tDCS has evidence of efficacy, given the limited information on treatment parameters and the negative results from recent large RCTs, it has been downgraded to a third-line recommendation for MDD 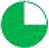 (Table 8.3).

**Table 8.3.**
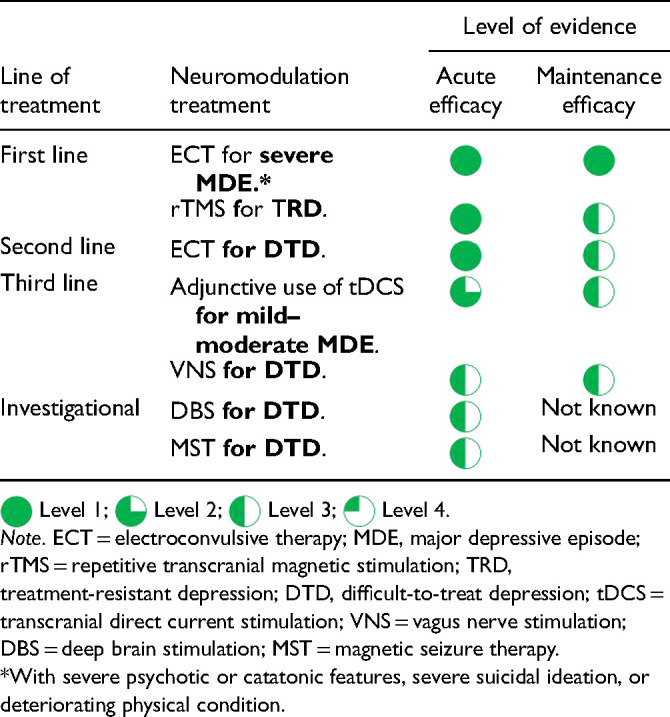
Summary Recommendations for Neuromodulation Treatments.

In some clinical situations, noninvasive neuromodulation treatments can be considered as first-choice treatments for MDD. For example, ECT can be used as first-line treatment in severe illness (e.g., MDE with psychotic or catatonic features, severe suicidal ideation, and deteriorating physical condition) or for patients who had a prior good response to ECT. Similarly, rTMS can be recommended as a first-choice treatment if there are tolerability concerns with medication options or if it was effective in a previous episode. Finally, except in emergent situations as mentioned above, rTMS should generally be considered before ECT, given its less invasive nature and that individuals may continue to work or attend school while undergoing rTMS. Although some studies show that prior poor response to ECT is associated with poor response to rTMS, other studies do not.

Given the lower level of evidence for efficacy and the greater associated risks, surgical neuromodulation treatments should generally be considered after noninvasive neuromodulation treatments. Currently, VNS is the only surgical neuromodulation treatment approved for TRD by regulatory agencies and is recommended as a third-line option (Table 8.3). DBS and MST are currently considered investigational due to limited available evidence.

## Conclusions

The 2023 update of the CANMAT depression guidelines provides a comprehensive and evidence-informed framework for managing MDD in adults. Healthcare providers can use the new and updated recommendations to collaboratively guide individuals with MDD through their care journey from assessment to evidence-based treatments, with the overall aim of optimizing their personal, functional, and quality of life outcomes.

## Supplemental Material

sj-docx-1-cpa-10.1177_07067437241245384 - Supplemental material for Canadian Network for Mood and Anxiety Treatments (CANMAT) 2023 Update on Clinical Guidelines for Management of Major Depressive Disorder in Adults: Réseau canadien pour les traitements de l'humeur et de l'anxiété (CANMAT) 2023 : Mise à jour des lignes directrices cliniques pour la prise en charge du trouble dépressif majeur chez les adultes Supplemental material, sj-docx-1-cpa-10.1177_07067437241245384 for Canadian Network for Mood and Anxiety Treatments (CANMAT) 2023 Update on Clinical Guidelines for Management of Major Depressive Disorder in Adults: Réseau canadien pour les traitements de l'humeur et de l'anxiété (CANMAT) 2023 : Mise à jour des lignes directrices cliniques pour la prise en charge du trouble dépressif majeur chez les adultes by Raymond W. Lam, Sidney H. Kennedy, Camelia Adams, Anees Bahji, Serge Beaulieu, Venkat Bhat, Pierre Blier, Daniel M. Blumberger, Elisa Brietzke, Trisha Chakrabarty, André Do, Benicio N. Frey, Peter Giacobbe, David Gratzer, Sophie Grigoriadis, Jeffrey Habert, M. Ishrat Husain, Zahinoor Ismail, Alexander McGirr, Roger S. McIntyre, Erin E. Michalak, Daniel J. Müller, Sagar V. Parikh, Lena S. Quilty, Arun V. Ravindran, Nisha Ravindran, Johanne Renaud, Joshua D. Rosenblat, Zainab Samaan, Gayatri Saraf, Kathryn Schade, Ayal Schaffer, Mark Sinyor, Claudio N. Soares, Jennifer Swainson, Valerie H. Taylor, Smadar V. Tourjman, Rudolf Uher, Michael van Ameringen, Gustavo Vazquez, Simone Vigod, Daphne Voineskos, Lakshmi N. Yatham and Roumen V. Milev in The Canadian Journal of Psychiatry

## References

[bibr141-] Balshem H Helfand M Schünemann M , et al. GRADE guidelines: 3. Rating the quality of evidence. J Clin Epidemiol 2011; 64:401–6. 21208779 10.1016/j.jclinepi.2010.07.015

[bibr142-] Herrman H Patel V Kieling C , et al. Time for united action on depression: a Lancet-World Psychiatric Association Commission. Lancet. 2022; 399:957–1022. 35180424 10.1016/S0140-6736(21)02141-3

[bibr143-] Lam RW Kennedy SH Parikh SV , et al. Canadian Network for Mood and Anxiety Treatments (CANMAT) 2016 clinical guidelines for the management of adults with major depressive disorder: Introduction and methods. Can J Psychiatry. 2016; 61:506–9. 27486152 10.1177/0706743716659061 PMC4994787

[bibr144-] Luo Y Chaimani A Furukawa TA , et al. Visualizing the evolution of evidence: Cumulative network meta-analyses of new generation antidepressants in the last 40 years. Res Synth Methods. 2021; 12:74–85. 32352639 10.1002/jrsm.1413 PMC7818396

[bibr145-] Parikh SV, Kcomt A, Fonseka TM, et al (Eds). The CHOICE–D Patient and Family Guide to Depression Treatment. Toronto, Mood Disorders Association of Ontario, 2018.

[bibr146-] Thornicroft G Chatterji S Evans-Lacko S , et al. Undertreatment of people with major depressive disorder in 21 countries. Br J Psychiatry. 2017; 210:119–124. 27908899 10.1192/bjp.bp.116.188078 PMC5288082

[bibr147-] GBD 2019 Mental Disorders Collaborators. Global, regional, and national burden of 12 mental disorders in 204 countries and territories, 1990-2019: A systematic analysis for the Global Burden of Disease Study 2019. Lancet Psychiatry 2022; 9:137-50. 10.1016/S2215-0366(21)00395-3 PMC8776563 35026139

[bibr148-] Fekadu A Demissie M Birhane R , et al. Under detection of depression in primary care settings in low and middle-income countries: a systematic review and meta-analysis. Syst Rev. 2022; 11:21. 10.1186/s13643-022-01893-9 PMC8818168 35123556

[bibr149-] Habtamu K Birhane R Demissie M , et al. Interventions to improve the detection of depression in primary healthcare: systematic review. Syst Rev. 2023; 12:25. 10.1186/s13643-023-02177-6 PMC9951508 36829262

[bibr150-] Hu J Wu T Damodaran S , et al. The effectiveness of collaborative care on depression outcomes for racial/ethnic minority populations in primary care: A systematic review. Psychosomatics 2020; 61:632–44. 32381258 10.1016/j.psym.2020.03.007 PMC7541409

[bibr151-] Joffres M Jaramillo A Dickinson J , et al. Recommendations on screening for depression in adults. CMAJ. 2013; 185:775–82. Erratum in: CMAJ. 2013; 185:1067. 23670157 10.1503/cmaj.130403 PMC3680556

[bibr152-] Köhler CA Evangelou E Stubbs B , et al. Mapping risk factors for depression across the lifespan: An umbrella review of evidence from meta-analyses and Mendelian randomization studies. J Psychiatr Res. 2018; 103:189–207. 29886003 10.1016/j.jpsychires.2018.05.020

[bibr153-] Lai HM Cleary M Sitharthan T , et al. Prevalence of comorbid substance use, anxiety and depressive disorders in epidemiological surveys, 1990-2014: A systematic review and meta-analysis. Drug Alcohol Depend. 2015; 154:1-13. 10.1016/j.drugalcdep.2015.05.031 26072219

[bibr154-] Levis B Sun Y He C , et al. Accuracy of the phq-2 alone and in combination with the phq-9 for screening to detect major depression: Systematic review and meta-analysis. JAMA 2020; 323:2290–300. 32515813 10.1001/jama.2020.6504 PMC7284301

[bibr155-] McKay MT Cannon M Chambers D , et al. Childhood trauma and adult mental disorder: A systematic review and meta-analysis of longitudinal cohort studies. Acta Psychiatr Scand 2021; 143:189–205. 33315268 10.1111/acps.13268

[bibr156-] Lee-Tauler SY Eun J Corbett D , A systematic review of interventions to improve initiation of mental health care among racial-ethnic minority groups. Psychiatric Services 2018; 69:628–47. 29716446 10.1176/appi.ps.201700382

[bibr157-] Patten SB Williams JV Lavorato DH , et al. Major depression in Canada: What has changed over the past 10 Years? Can J Psychiatry. 2016; 61:80–5. 27253698 10.1177/0706743715625940 PMC4784240

[bibr158-] Salk RH Hyde JS Abramson LY . Gender differences in depression in representative national samples: Meta-analyses of diagnoses and symptoms. Psychol Bull. 2017; 143:783–822. 28447828 10.1037/bul0000102 PMC5532074

[bibr159-] Thom R Silbersweig DA Boland RJ . Major depressive disorder in medical illness: A review of assessment, prevalence, and treatment options. Psychosom Med 2019; 81:246–55. 30720699 10.1097/PSY.0000000000000678

[bibr160-] US Preventive Services Task Force; Barry MJ, Nicholson WK, et al. Screening for depression and suicide risk in adults: US Preventive Services Task Force recommendation statement. JAMA. 2023; 329:2057-2067. 10.1001/jama.2023.9297 37338872

[bibr161-] Delgadillo J Ali S Fleck K , et al. Stratified care vs stepped care for depression: A cluster randomized clinical trial. JAMA Psychiatry 2022; 79:101–8. 34878526 10.1001/jamapsychiatry.2021.3539 PMC8655665

[bibr162-] Dong M Zeng LN Lu L , et al. Prevalence of suicide attempt in individuals with major depressive disorder: a meta-analysis of observational surveys. Psychol Med. 2019; 49:1691–1704. 30178722 10.1017/S0033291718002301

[bibr163-] Hawton K Lascelles K Pitman A , et al. Assessment of suicide risk in mental health practice: shifting from prediction to therapeutic assessment, formulation, and risk management. Lancet Psychiatry. 2022; 9:922–928. 35952701 10.1016/S2215-0366(22)00232-2

[bibr164-] Heissel A Heinen D Brokmeier LL , et al. Exercise as medicine for depressive symptoms? A systematic review and meta-analysis with meta-regression. Br J Sports Med. 2023; 57:1049–1057. 36731907 10.1136/bjsports-2022-106282 PMC10423472

[bibr165-] Hu J Wu T Damodaran S , et al. The effectiveness of collaborative care on depression outcomes for racial/ethnic minority populations in primary care: A systematic review. Psychosomatics 2020; 61:632–44. 32381258 10.1016/j.psym.2020.03.007 PMC7541409

[bibr166-] Kruizinga J Liemburg E Burger H , et al. Pharmacological treatment for psychotic depression. Cochrane Database Syst Rev. 2021; 12:CD004044. 10.1002/14651858.CD004044.pub5 PMC8651069 34875106

[bibr167-] Levis B Sun Y He C , et al. Accuracy of the PHQ-2 alone and in combination with the PHQ-9 for screening to detect major depression: Systematic review and meta-analysis. JAMA. 2020; 323:2290–2300. 32515813 10.1001/jama.2020.6504 PMC7284301

[bibr168-] Marshall T Stellick C Abba-Aji A , et al. The impact of shared decision-making on the treatment of anxiety and depressive disorders: a systematic review. BJPsych Open 2021; 7:e189, 1–12. 10.1192/bjo.2021.1050 PMC8612021 34784989

[bibr169-] Matison AP Mather KA Flood VM , et al. Associations between nutrition and the incidence of depression in middle-aged and older adults: A systematic review and meta-analysis of prospective observational population-based studies. Ageing Res Rev. 2021; 70:101403. 10.1016/j.arr.2021.101403 34246793

[bibr170-] Parikh SV, Kcomt A, Fonseka TM, et al (Eds). The CHOICE–D Patient and Family Guide to Depression Treatment. Toronto, Mood Disorders Association of Ontario, 2018.

[bibr171-] Statistics Canada. Table 13-10-0392-01. Deaths and age-specific mortality rates, by selected grouped causes 2018-2022. DOI: 10.25318/1310039201-eng, Accessed December 18, 2023.

[bibr172-] Tao L Jiang R Zhang K , et al. Light therapy in non-seasonal depression: An update meta-analysis. Psychiatry Res. 2020; 291:113247. 10.1016/j.psychres.2020.113247 32622169

[bibr173-] van Straten A Hill J Richards DA , et al. Stepped care treatment delivery for depression: a systematic review and meta-analysis. Psychol Med. 2015; 45:231–46. 25065653 10.1017/S0033291714000701

[bibr174-] Anik E West RM Cardno AG , et al. Culturally adapted psychotherapies for depressed adults: a systematic review and meta-analysis. Journal of affective disorders. 2021; 278:296–310. 32979561 10.1016/j.jad.2020.09.051

[bibr175-] Banyard H Behn AJ Delgadillo J . Personality disorders and their relation to treatment outcomes in cognitive behavioural therapy for depression: A systematic review and meta-analysis. Cogn Ther Res. 2021; 45:561–76.

[bibr176-] Boschloo L Bekhuis E Weitz ES , et al. The symptom-specific efficacy of antidepressant medication vs. cognitive behavioral therapy in the treatment of depression: results from an individual patient data meta-analysis. World Psychiatry. 2019; 18:183–191. 31059603 10.1002/wps.20630 PMC6502416

[bibr177-] Bunka M Wong G Kim D , et al. Evaluating treatment outcomes in pharmacogenomic-guided care for major depression: A rapid review and meta-analysis. Psychiatry Res. 2023; 321:115102. 10.1016/j.psychres.2023.115102 36780865

[bibr178-] Carlucci L Saggino A Balsamo M . On the efficacy of the unified protocol for transdiagnostic treatment of emotional disorders: a systematic review and meta-analysis. Clinical Psychology Review. 2021; 87:101999. 10.1016/j.cpr.2021.101999 34098412

[bibr179-] Cipriani A Furukawa TA Salanti G , et al. Comparative efficacy and acceptability of 21 antidepressant drugs for the acute treatment of adults with major depressive disorder: a systematic review and network meta-analysis. Lancet. 2018; 391:1357–1366. 29477251 10.1016/S0140-6736(17)32802-7 PMC5889788

[bibr180-] Coupland C Hill T Morriss R , et al. Antidepressant use and risk of suicide and attempted suicide or self harm in people aged 20 to 64: cohort study using a primary care database. BMJ. 2015; 350:h517. 10.1136/bmj.h517 PMC4353276 25693810

[bibr181-] Cuijpers P de Wit L Kleiboer A , et al. Problem-solving therapy for adult depression: an updated meta-analysis. Eur Psychiatry. 2018; 48:27–37. 29331596 10.1016/j.eurpsy.2017.11.006

[bibr182-] Cuijpers P Karyotaki E de Wit L , et al. The effects of fifteen evidence-supported therapies for adult depression: a meta-analytic review. Psychother Res. 2020; 30:279–93. 31394976 10.1080/10503307.2019.1649732

[bibr183-] Cuijpers P Noma H Karyotaki E , et al. A network meta-analysis of the effects of psychotherapies, pharmacotherapies and their combination in the treatment of adult depression. World Psychiatry 2020; 19:92–107. 31922679 10.1002/wps.20701 PMC6953550

[bibr184-] Cuijpers P Quero S Noma H , et al. Psychotherapies for depression: a network meta-analysis covering efficacy, acceptability and long-term outcomes of all main treatment types. World Psychiatry. 2021; 20:283–293. 34002502 10.1002/wps.20860 PMC8129869

[bibr185-] Driessen E Dekker JJM Peen J , et al. The efficacy of adding short-term psychodynamic psychotherapy to antidepressants in the treatment of depression: A systematic review and meta-analysis of individual participant data. Clin Psychol Rev 2020; 80101886. 10.1016/j.cpr.2020.101886 32650213

[bibr186-] Goldberg SB Tucker RP Greene PA , et al. Mindfulness-based cognitive therapy for the treatment of current depressive symptoms: a meta-analysis. Cogn Behav Ther. 2019; 48:445–62. 30732534 10.1080/16506073.2018.1556330 PMC6687569

[bibr187-] Guidi J Fava GA . Sequential combination of pharmacotherapy and psychotherapy in major depressive disorder: A systematic review and meta-analysis. JAMA Psychiatry 2021; 78:261–269. 33237285 10.1001/jamapsychiatry.2020.3650 PMC7689568

[bibr188-] Marques A Ihle A Souza A , et al. Religious-based interventions for depression: A systematic review and meta-analysis of experimental studies. J Affect Disord. 2022; 309:289–296. 35500682 10.1016/j.jad.2022.04.126

[bibr189-] Negt P Brakemeier EL Michalak J , et al. The treatment of chronic depression with cognitive behavioural analysis system of psychotherapy: A systematic review and meta-analysis of randomized-controlled clinical trials. Brain Behav. 2016; 6:e00486. 10.1002/brb3.486 PMC4864084 27247856

[bibr190-] Philipp R Kriston L Lanio J , et al. Effectiveness of metacognitive interventions for mental disorders in adults – A systematic review and meta-analysis (METACOG). Clin Psychol Psychother. 2019; 26:227–40. 30456821 10.1002/cpp.2345

[bibr191-] Sarris J Marx W Ashton MM , et al. Plant-based nedicines (phytoceuticals) in the treatment of psychiatric disorders: A meta-review of meta-analyses of randomized controlled trials. Can J Psychiatry. 2021; 66:849–862. 33596697 10.1177/0706743720979917 PMC8573706

[bibr192-] Uphoff E Ekers D Robertson L , et al. Behavioural activation therapy for depression in adults. Cochrane Database System Rev. 2020; 7:25–44. 10.1002/14651858.CD013305.pub2 PMC7390059 32628293

[bibr193-] Wagner G Schultes M-T Titscher V , et al. Efficacy and safety of levomilnacipran, vilazodone and vortioxetine compared with other second-generation antidepressants for major depressive disorder in adults: A systematic review and network meta-analysis. J Affect Disord 2018; 228:1–12. 29197738 10.1016/j.jad.2017.11.056

[bibr194-] Wang YY Li XH Zheng W , et al. Mindfulness-based interventions for major depressive disorder: a comprehensive meta-analysis of randomized controlled trials. J Affect Disord 2018; 229:429–36. 29331704 10.1016/j.jad.2017.12.093

[bibr195-] Abd-Alrazaq AA Rababeh A Alajlani M , et al. Effectiveness and safety of using chatbots to improve mental health: Systematic review and meta-analysis. J Med Internet Res. 20200; 22:e16021. 10.2196/16021 PMC7385637 32673216

[bibr196-] Ahern E Kinsella S Semkovska M . Clinical efficacy and economic evaluation of online cognitive behavioral therapy for major depressive disorder: a systematic review and meta-analysis. Expert Rev Pharmacoecon Outcomes Res. 2018; 18:25–41. 29145746 10.1080/14737167.2018.1407245

[bibr197-] Bhuyan SS Kim h Isehunwa OO , et al. Privacy and security issues in mobile health: Current research and future directions. Health Policy Technol 2017; 6: 188–191.

[bibr198-] Borghouts J Eikey E Mark G , et al. Barriers to and facilitators of user engagement with digital mental health interventions: Systematic review. J Med Internet Res. 2021 4; 23:e24387. 10.2196/24387 PMC8074985 33759801

[bibr199-] Cuijpers P Noma H Karyotaki E , et al. Effectiveness and acceptability of cognitive behavior therapy delivery formats in adults with depression: A network meta-analysis. JAMA Psychiatry. 2019; 76:700–707. 30994877 10.1001/jamapsychiatry.2019.0268 PMC6583673

[bibr200-] Firth J Torous J Nicholas J , et al. The efficacy of smartphone-based mental health interventions for depressive symptoms: a meta-analysis of randomized controlled trials. World Psychiatry. 2017; 16:287–298. 28941113 10.1002/wps.20472 PMC5608852

[bibr201-] Gratzer D Torous J Lam RW , et al. Our digital moment: Innovations and opportunities in digital mental health care. Can J Psychiatry. 2021; 66:5–8. 32603188 10.1177/0706743720937833 PMC7890581

[bibr202-] Huguet A Miller A Kisely S , et al. A systematic review and meta-analysis on the efficacy of Internet-delivered behavioral activation. J Affect Disord. 2018; 235:27–38. 29649708 10.1016/j.jad.2018.02.073

[bibr203-] Josephine K Josefine L Philipp D , et al. Internet- and mobile-based depression interventions for people with diagnosed depression: A systematic review and meta-analysis. J Affect Disord. 2017; 223:28–40. 28715726 10.1016/j.jad.2017.07.021

[bibr204-] Linardon J Fuller-Tyszkiewicz M . Attrition and adherence in smartphone-delivered interventions for mental health problems: A systematic and meta-analytic review. J Consult Clin Psychol. 2020; 88:1–13. 31697093 10.1037/ccp0000459

[bibr205-] O’Loughlin K Neary M Adkins EC , et al. Reviewing the data security and privacy policies of mobile apps for depression. Internet Interv. 2018; 15:110–115. 30792962 10.1016/j.invent.2018.12.001 PMC6371412

[bibr206-] Ramos G Ponting C Labao JP , et al. Considerations of diversity, equity, and inclusion in mental health apps: A scoping review of evaluation frameworks. Behav Res Ther. 2021; 147:103990. 10.1016/j.brat.2021.103990 34715396

[bibr207-] Thase ME Wright JH Eells TD , et al. Improving the efficiency of psychotherapy for depression: Computer-assisted versus standard CBT. Am J Psychiatry. 2018; 175:242–250. 28969439 10.1176/appi.ajp.2017.17010089 PMC5848497

[bibr208-] Torous J Bucci S Bell IH , et al. The growing field of digital psychiatry: current evidence and the future of apps, social media, chatbots, and virtual reality. World Psychiatry. 2021; 20:318–335. 34505369 10.1002/wps.20883 PMC8429349

[bibr209-] Zelmer J van Hoof K Notarianni M , et al. An assessment framework for e-mental health apps in Canada: Results of a modified Delphi process. JMIR Mhealth Uhealth. 2018; 6:e10016. 10.2196/10016 PMC6056739 29986846

[bibr210-] Carter G Milner A McGill K , et al. Predicting suicidal behaviours using clinical instruments: systematic review and meta-analysis of positive predictive values for risk scales. Br J Psychiatry 2017; 210:387–95. 28302700 10.1192/bjp.bp.116.182717

[bibr211-] Connors EH Douglas S Jensen-Doss A , et al. What gets measured gets done: How mental health agencies can leverage measurement-based care for better patient care, clinician supports, and organizational Goals. Adm Policy Ment Health. 2021; 48:250–65. 32656631 10.1007/s10488-020-01063-w PMC7854781

[bibr212-] Hong RH Murphy JK Michalak EE , et al. Implementing measurement-based care for depression: Practical solutions for psychiatrists and primary care physician. Neuropsychiatr Dis Treat 2021;1779–90. 33469295 10.2147/NDT.S283731 PMC7813452

[bibr213-] Kendrick T El-Gohary M Stuart B , et al. Routine use of patient reported outcome measures (PROMs) for improving treatment of common mental health disorders in adults. Cochrane Database Syst Rev. 2016;7:CD011119. 10.1002/14651858.CD011119.pub2 PMC6472430 27409972

[bibr214-] Lambert MJ Whipple JL Kleinstäuber M . Collecting and delivering progress feedback: A meta-analysis of routine outcome monitoring. Psychotherapy 2018; 55:520–37. 30335463 10.1037/pst0000167

[bibr215-] Lewis CC Boyd M Puspitasari A , et al. Implementing measurement-based care in behavioral health: A review. JAMA Psychiatry 2019; 76:324–35. 30566197 10.1001/jamapsychiatry.2018.3329 PMC6584602

[bibr216-] Metz MJ Veerbeek MA Franx GC , et al. A National Quality Improvement Collaborative for the clinical use of outcome measurement in specialised mental healthcare: results from a parallel group design and a nested cluster randomised controlled trial. BJPsych Open. 2017; 3:106–112. 28507769 10.1192/bjpo.bp.116.004366 PMC5410407

[bibr217-] Shimokawa K Lambert MJ Smart DW . Enhancing treatment outcome of patients at risk of treatment failure: Meta-analytic and mega-analytic review of a psychotherapy quality assurance system. J Consult Clin Psychol. 2010; 78:298–311. 20515206 10.1037/a0019247

[bibr218-] Taylor VH Sockalingam S Hawa R Hahn M . Canadian Adult Obesity Clinical Practice Guidelines: The Role of Mental Health in Obesity Management. Available from: https://obesitycanada.ca/guidelines/mentalhealth , accessed December 13, 2023.

[bibr219-] Treviño-Alvarez AM Sánchez-Ruiz JA Barrera FJ , et al. Weight changes in adults with major depressive disorder: A systematic review and meta-analysis of prospective studies. J Affect Disord 2023; 332:1–8. 36963517 10.1016/j.jad.2023.03.050

[bibr220-] Wilde E Kim H Schulz P , et al. Laboratory testing and neuroimaging studies in psychiatry. In: The American Psychiatric Association Publishing Textbook of Psychiatry, Seventh Edition. American Psychiatric Association Publishing; Washington, DC, 2019.

[bibr221-] Zhu M Hong RH Yang T , et al. The efficacy of measurement-based care for depressive disorders: Systematic review and meta-analysis of randomized controlled trials. J Clin Psychiatry 2021; 82:21r14034. 10.4088/JCP.21r14034 34587377

[bibr222-] Berwian IM Walter H Seifritz E , et al. Predicting relapse after antidepressant withdrawal - a systematic review. Psychol Med. 2017; 47:426–437. 27786144 10.1017/S0033291716002580 PMC5244448

[bibr223-] Breedvelt JJF Warren FC Segal Z , et al. Continuation of antidepressants vs sequential psychological interventions to prevent relapse in depression: An individual participant data meta-analysis. JAMA Psychiatry. 2021; 78:868–875. 34009273 10.1001/jamapsychiatry.2021.0823 PMC8135055

[bibr224-] Buckman JEJ Underwood A Clarke K , et al. Risk factors for relapse and recurrence of depression in adults and how they operate: A four-phase systematic review and meta-synthesis. Clin Psychol Rev. 2018; 64:13–38. 30075313 10.1016/j.cpr.2018.07.005 PMC6237833

[bibr225-] DeRubeis RJ Zajecka J Shelton RC , et al. Prevention of recurrence after recovery from a major depressive episode with antidepressant medication alone or in combination with cognitive behavioral therapy: Phase 2 of a 2-phase randomized clinical trial. JAMA Psychiatry 2022; 77:237–45. 10.1001/jamapsychiatry.2019.3900 PMC6902236 31799993

[bibr226-] Guidi J Fava GA . Sequential combination of pharmacotherapy and psychotherapy in major depressive disorder: A systematic review and meta-analysis. JAMA Psychiatry. 2021; 78:261–269. 33237285 10.1001/jamapsychiatry.2020.3650 PMC7689568

[bibr227-] Kato M Hori H Inoue T , et al. Discontinuation of antidepressants after remission with antidepressant medication in major depressive disorder: a systematic review and meta-analysis. Mol Psychiatry. 2021; 26:118–133. 32704061 10.1038/s41380-020-0843-0 PMC7815511

[bibr228-] Kishi T Sakuma K Hatano M , et al. Relapse and its modifiers in major depressive disorder after antidepressant discontinuation: meta-analysis and meta-regression. Mol Psychiatry. 2023; 28:974–976. 36564488 10.1038/s41380-022-01920-0 PMC10005929

[bibr229-] Lewis G Marston L Duffy L , et al. Maintenance or discontinuation of antidepressants in primary care. New Engl J Med, 2021; 385:1257–67. 34587384 10.1056/NEJMoa2106356

[bibr230-] Maund E Stuart B Moore M , et al. Managing antidepressant discontinuation: A systematic review. Ann Fam Med. 2019; 17:52–60. 30670397 10.1370/afm.2336 PMC6342590

[bibr231-] Van Leeuwen E van Driel ML Horowitz MA , et al. Approaches for discontinuation versus continuation of long-term antidepressant use for depressive and anxiety disorders in adults. Cochrane Database Syst Rev. 2021; 4:CD013495. 10.1002/14651858.CD013495.pub2 PMC8092632 33886130

[bibr232-] Wojnarowski C Firth N Finegan M , et al. Predictors of depression relapse and recurrence after cognitive behavioural therapy: a systematic review and meta-analysis. Behav Cogn Psychother. 2019; 47:514–529. 30894231 10.1017/S1352465819000080

[bibr233-] Zhou D Zhou X Lin Q , et al. Nonpharmacological interventions for relapse prevention in unipolar depression: A network meta-analysis. J Affect Disord. 2021; 282:1255–1262. 33601704 10.1016/j.jad.2021.01.025

[bibr234-] Bahji A Mesbah-Oskui L . Comparative efficacy and safety of stimulant-type medications for depression: A systematic review and network meta-analysis. J Affect Disord. 2021; 292:416–423. 34144366 10.1016/j.jad.2021.05.119

[bibr235-] Black N Stockings E Campbell G , et al. Cannabinoids for the treatment of mental disorders and symptoms of mental disorders: a systematic review and meta-analysis. Lancet Psychiatry. 2019; 6:995–1010. 31672337 10.1016/S2215-0366(19)30401-8 PMC6949116

[bibr236-] Bschor T Kern H Henssler J , et al. Switching the antidepressant after nonresponse in adults with major depression: A systematic literature search and meta-analysis. J Clin Psychiatry. 2018; 79:16r 10749. 10.4088/JCP.16r10749 27929611

[bibr237-] Davies P Ijaz S Williams CJ , et al. Pharmacological interventions for treatment-resistant depression in adults. Cochrane Database Syst Rev. 2019; 12:CD010557. 10.1002/14651858.CD010557.pub2 PMC6916711 31846068

[bibr238-] Goh KK Chen CH Chiu YH , et al. Lamotrigine augmentation in treatment-resistant unipolar depression: A comprehensive meta-analysis of efficacy and safety. J Psychopharmacol. 2019; 33:700–713. 31081449 10.1177/0269881119844199

[bibr239-] Henssler J Alexander D Schwarzer G Bschor T Baethge C . Combining antidepressants vs antidepressant monotherapy for treatment of patients with acute Depression: A systematic review and meta-analysis. JAMA Psychiatry. 2022; 79:300–312. 35171215 10.1001/jamapsychiatry.2021.4313 PMC8851370

[bibr240-] Ijaz S Davies P Williams CJ , et al. Psychological therapies for treatment-resistant depression in adults. Cochrane Database Syst Rev. 2018; 5:CD010558. 10.1002/14651858.CD010558.pub2 PMC6494651 29761488

[bibr241-] Lima TM Visacri MB , et al. Use of ketamine and esketamine for depression: an overview of systematic reviews with meta-analysès. Eur J Clin Pharmacol. 2022; 78:311–338. 34705064 10.1007/s00228-021-03216-8

[bibr242-] McAllister-Williams RH Arango C Blier P , et al. The identification, assessment and management of difficult-to-treat depression: An international consensus statement. J Affect Disord. 2020; 267:264–282. 32217227 10.1016/j.jad.2020.02.023

[bibr243-] Nuñez NA Joseph B Pahwa M , et al. Augmentation strategies for treatment resistant major depression: A systematic review and network meta-analysis. J Affect Disord. 2022; 302:385–400. 34986373 10.1016/j.jad.2021.12.134 PMC9328668

[bibr244-] Rosenblat JD Husain MI Lee Y , et al. The Canadian Network for Mood and Anxiety Treatments (CANMAT) Task Force report: Serotonergic psychedelic treatments for major depressive disorder. Can J Psychiatry. 2023; 68:5–21. 35975555 10.1177/07067437221111371 PMC9720483

[bibr245-] Smith-Apeldoorn SY Veraart JK Spijker J , et al. Maintenance ketamine treatment for depression: a systematic review of efficacy, safety, and tolerability. Lancet Psychiatry. 2022; 9:907–921. 36244360 10.1016/S2215-0366(22)00317-0

[bibr246-] Swainson J McGirr A Blier P , et al. The Canadian Network for Mood and Anxiety Treatments (CANMAT) Task Force recommendations for the use of racemic ketamine in adults with major depressive disorder. Can J Psychiatry. 2021; 66:113–125. 33174760 10.1177/0706743720970860 PMC7918868

[bibr247-] Tourjman SV Buck G Jutras-Aswad D , et al. Canadian Network for Mood and Anxiety Treatments (CANMAT) Task Force report: A systematic review and recommendations of cannabis use in bipolar disorder and major depressive disorder. Can J Psychiatry 2023; 68:299–311. 35711159 10.1177/07067437221099769 PMC10192829

[bibr248-] Xiong J Lipsitz O Chen-Li D , et al. The acute antisuicidal effects of single-dose intravenous ketamine and intranasal esketamine in individuals with major depression and bipolar disorders: A systematic review and meta-analysis. J Psychiatr Res. 2021; 134:57–68. 33360864 10.1016/j.jpsychires.2020.12.038

[bibr249-] Yan Y Yang X Wang M , et al. Efficacy and acceptability of second-generation antipsychotics with antidepressants in unipolar depression augmentation: a systematic review and network meta-analysis. Psychol Med. 2022; 52:2224–2231. 35993319 10.1017/S0033291722001246

[bibr250-] Blumberger DM Vila-Rodriguez F Thorpe KE , et al. Effectiveness of theta burst versus high-frequency repetitive transcranial magnetic stimulation in patients with depression (Three-D): A randomised non-inferiority trial. Lancet 2018 8; 391:1683–92. 10.1016/S0140-6736(18)30295-2 29726344

[bibr251-] Bottomley JM LeReun C Diamantopoulos A , et al. Vagus nerve stimulation (VNS) therapy in patients with treatment resistant depression: A systematic review and meta-analysis. Compr Psychiatry 2019; 98:152156. 10.1016/j.comppsych.2019.152156 31978785

[bibr252-] Burkhardt G Kumpf U Crispin A , et al. Transcranial direct current stimulation as an additional treatment to selective serotonin reuptake inhibitors in adults with major depressive disorder in Germany (DepressionDC): A triple-blind, randomised, sham-controlled, multicentre trial. Lancet 2023; 402:545–54. 37414064 10.1016/S0140-6736(23)00640-2

[bibr253-] Cao X Deng C Su X , et al. Response and remission rates following high-frequency vs. Low-frequency repetitive transcranial magnetic stimulation (rTMS) over right DLPFC for treating major depressive disorder (MDD): A meta-analysis of randomized, double-blind trials. Front Psychiatry 2018; 9:413. 10.3389/fpsyt.2018.00413 PMC6137236 30245641

[bibr254-] Chen M Yang X Liu C , et al. Comparative efficacy and cognitive function of magnetic seizure therapy vs. electroconvulsive therapy for major depressive disorder: A systematic review and meta-analysis. Transl Psychiatry 2021; 11:437. 10.1038/s41398-021-01560-y PMC8380249 34420033

[bibr255-] Chu HT Cheng CM Liang CS , et al. Efficacy and tolerability of theta-burst stimulation for major depression: A systematic review and meta-analysis. Prog Neuropsychopharmacol Biol Psychiatry 2021; 106:110168. 10.1016/j.pnpbp.2020.110168 33166668

[bibr256-] Duma A Maleczek M Panjikaran B , et al. Major adverse cardiac events and mortality associated with electroconvulsive therapy: A systematic review and meta-analysis. Anesthesiology 2019; 130:83–91. 30557212 10.1097/ALN.0000000000002488 PMC6300062

[bibr257-] Kisely S Li A Warren N Siskind D . A systematic review and meta-analysis of deep brain stimulation for depression. Depress Anxiety 2018; 35:468–80. 29697875 10.1002/da.22746

[bibr258-] Landry M Moreno A Patry S , et al. Current practices of electroconvulsive therapy in mental disorders: A systematic review and meta-analysis of short and long-term cognitive effects. J ECT 2021; 37:119–27. 33009218 10.1097/YCT.0000000000000723

[bibr260-] Mutz J Edgcumbe DR Brunoni AR , et al. Efficacy and acceptability of non-invasive brain stimulation for the treatment of adult unipolar and bipolar depression: A systematic review and meta-analysis of randomised sham-controlled trials. Neurosci Biobehav Rev 2018; 92:291–303. 29763711 10.1016/j.neubiorev.2018.05.015

[bibr261-] Mutz J Vipulananthan V Carter B , et al. Comparative efficacy and acceptability of non-surgical brain stimulation for the acute treatment of major depressive episodes in adults: Systematic review and network meta-analysis. BMJ 2019; 364:l1079. 10.1136/bmj.l1079 PMC6435996 30917990

[bibr262-] Rowland T Mann R Azeem S . The efficacy and tolerability of continuation and maintenance electroconvulsive therapy for depression: A systematic review of randomized and observational studies. J ECT 2023; 39:141–50. 36961277 10.1097/YCT.0000000000000914

[bibr263-] Semkovska M Knittle H Leahy J , et al. Subjective cognitive complaints and subjective cognition following electroconvulsive therapy for depression: A systematic review and meta-analysis. Aust N Z J Psychiatry 2023; 57:21–33. 35362328 10.1177/00048674221089231

[bibr264-] Wang J Luo H Schulke R , et al. Is transcranial direct current stimulation, alone or in combination with antidepressant medications or psychotherapies, effective in treating major depressive disorder? A systematic review and meta-analysis. BMC Med 2021; 19:319. 10.1186/s12916-021-02181-4 PMC8680114 34915885

