# Supplementary material for: Canadian Network for Mood and Anxiety Treatments (CANMAT) 2023 Update on Clinical Guidelines for Management of Major Depressive Disorder in Adults: Réseau canadien pour les traitements de l'humeur et de l'anxiété (CANMAT) 2023 : Mise à jour des lignes directrices cliniques pour la prise en charge du trouble dépressif majeur chez les adultes
Source: Can J Psychiatry. 2024 May 6;69(9):641–87. doi: 10.1177/07067437241245384 (PMC11351064; doi:10.1177/07067437241245384)

**Supplementary Materials**

**Supplementary Figure e1. PRISMA diagram for literature search, January 1, 2015 to May 31, 2023.**


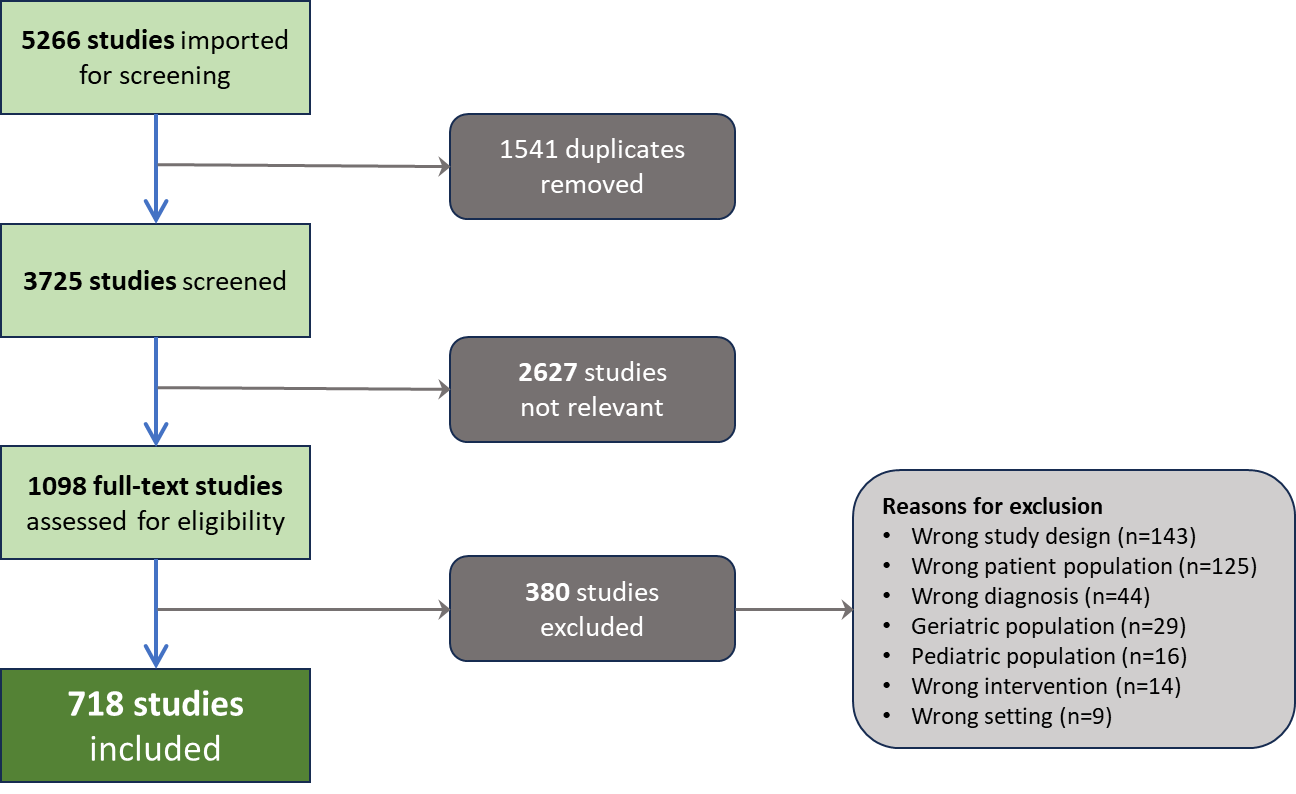

Supplement: sj-docx-1-cpa-10.1177_07067437241245384 - Supplemental material for Canadian Network for Mood and Anxiety Treatments (CANMAT) 2023 Update on Clinical Guidelines for Management of Major Depressive Disorder in Adults: Réseau canadien pour les traitements de l'humeur et de l'anxiété (CANMAT) 2023 : Mis [file sj-docx-1-cpa-10.1177_07067437241245384.docx]
